# Genotype-Specific Activation of Autophagy during Heat Wave in Wheat

**DOI:** 10.3390/cells13141226

**Published:** 2024-07-20

**Authors:** Kathleen Hickey, Yunus Şahin, Glenn Turner, Taras Nazarov, Vadim Jitkov, Mike Pumphrey, Andrei Smertenko

**Affiliations:** 1 Institute of Biological Chemistry, Washington State University, Pullman, WA 99163, USAyunus.sahin@wsu.edu (Y.Ş.); gturner@wsu.edu (G.T.); taras.nazarov@wsu.edu (T.N.); 2 Department of Crop and Soil Sciences, Washington State University, Pullman, WA 99163, USA; vjitkov@wsu.edu (V.J.); m.pumphrey@wsu.edu (M.P.)

**Keywords:** autophagy, heat, drought, tolerance, yield, ATG8, ATG7, NBR1

## Abstract

Recycling of unnecessary or dysfunctional cellular structures through autophagy plays a critical role in cellular homeostasis and environmental resilience. Therefore, the autophagy trait may have been unintentionally selected in wheat breeding programs for higher yields in arid climates. This hypothesis was tested by measuring the response of three common autophagy markers, ATG7, ATG8, and NBR1, to a heat wave under reduced soil moisture content in 16 genetically diverse spring wheat landraces originating from different geographical locations. We observed in the greenhouse trials that ATG8 and NBR1 exhibited genotype-specific responses to a 1 h, 40 °C heat wave, while ATG7 did not show a consistent response. Three genotypes from Uruguay, Mozambique, and Afghanistan showed a pattern consistent with higher autophagic activity: decreased or stable abundance of both ATG8 and NBR1 proteins, coupled with increased transcription of *ATG8* and *NBR1*. In contrast, three genotypes from Pakistan, Ethiopia, and Egypt exhibited elevated ATG8 protein levels alongside reduced or unaltered *ATG8* transcript levels, indicating a potential suppression or no change in autophagic activity. Principal component analysis demonstrated a correlation between lower abundance of ATG8 and NBR1 proteins and higher yield in the field trials. We found that (i) the combination of heat and drought activated autophagy only in several genotypes, suggesting that despite being a resilience mechanism, autophagy is a heat-sensitive process; (ii) higher autophagic activity correlates positively with greater yield; (iii) the lack of autophagic activity in some high-yielding genotypes suggests contribution of alternative stress-resilient mechanisms; and (iv) enhanced autophagic activity in response to heat and drought was independently selected by wheat breeding programs in different geographic locations.

## 1. Introduction

Bread wheat, *Triticum aestivum*, is commonly cultivated by dryland farming, which relies solely on rainfall to provide water during the growing season [1]. The latter makes wheat production vulnerable to drought. High ambient temperature occurring concurrently with drought compounds the yield losses [2]. Global climate change is predicted to increase the frequency of the episodes of extreme weather, leading to a reduction in wheat production worldwide [3], and our dependence on wheat means that sustaining food security relies on breeding resilient varieties [4]. Breeding efficiency could be advanced through the genomic selection of parents for markers associated with physiological mechanisms conferring tolerance to environmental stress [1,5].

Although marker-assisted selection facilitates the breeding of varieties with desired characteristics, drought and heat stress resilience are quantitative traits with numerous minor contributing loci strongly affected by environmental interactions [6,7]. As the behavior of individual quantitative loci could be inconsistent across different environments, identification of robust genetic markers requires mapping populations with high genetic diversity, such as wild relatives and landraces [8,9]. Advances in genomic sequencing, Quantitative Trait Loci (QTL) mapping, genome-wide associations, and global databases facilitate the mining of tolerance traits [10,11]. The use of nested-associated mapping (NAM) populations aids in breeding efforts by combining the linkage analysis and GWAS to discover novel QTLs and genetic markers that could be used as targets for introgression into elite cultivars [12,13]. Successful identification of the markers relies on sensitive phenotyping tools for the stress tolerance traits. The development of such tools requires information about plant responses to stress at the molecular, cellular, organ, and whole-plant levels.

Drought stress causes a decrease in plant growth, reduction in leaf area, closure of stomata, inhibition of photosynthesis, and an increase in photorespiration [14]. Heat stress decreases the stability of proteins, membranes, and other cellular components and compromises the activity of enzymes essential to plant metabolism (reviewed in [14,15,16]). The combination of drought and heat stress compounds the molecular and physiological effects by intensifying the production of reactive oxygen species (ROS) that can cause oxidative damage to proteins, lipids, and nucleic acids. Excessive oxidative damage leads to cell death and, ultimately, to loss of plant productivity and yield [14,17,18,19,20,21,22].

Cells can ameliorate the effects of oxidative damage by recycling the damaged cellular components through a highly conserved mechanism called macroautophagy (hereafter referred to as autophagy). Autophagy occurs via the formation of a double membrane vesicle on the endoplasmic reticulum, called the autophagosome, which encloses cytoplasmic components and then delivers them to the vacuole [23]. The autophagy pathway and machinery were first characterized in yeast [24], followed by the identification of almost all core orthologs of *AuTophaGy* (*ATG*) genes in plants. There are over 40 autophagy-related genes in *Arabidopsis thaliana* and 108 putative genes in bread wheat [25]. Components of the autophagy machinery could be classified into clusters based on their functions: (1) the ATG1-ATG13 kinase complex initiates the formation of autophagosome precursor, phagophore; (2) ATG9, ATG2, and ATG18 mediate lipids delivery for expanding the phagophore; (3) the phosphatidylinositol 3-kinase complex drives vesicle nucleation, autophagosome formation, and trafficking to vacuole; (4) conjugation of ATG8 to phosphatidylethanolamine (PE) contributes to phagophore membrane expansion and cargo recruitment; (5) ATG5-ATG12-ATG16 complex together with ATG4, ATG7, and ATG3 catalyze conjugation of ATG8 to phosphatidylethanolamine; and (6) ATG10 and ATG7 contribute to assembly of ATG5-ATG12-ATG16 complex (reviewed by [23,26,27]).

Autophagy plays a role in tolerance to many stresses, including oxidative stress induced by the application of H_2_O_2_ or methyl viologen [28,29]; nutrient deficiencies and starvation [30,31]; salinity [29,32]; hypoxia [33]; drought [32,34,35,36,37,38]; and heat [39,40,41]. Overexpression of *ATG8* increases tolerance to nitrogen starvation in *A. thaliana* [42]. Overexpression of *ATG5* and *ATG7* was shown to increase ATG8 lipidation and autophagic activity, leading to greater resistance to pathogens and oxidative stress in *A. thaliana* [43]. Hence, the autophagic pathway has potential as an important breeding target for advancing stress resilience in plants. ATG8 is frequently used as a marker for assessing the overall activity and progression of the autophagic pathway [44]. ATG8 provides a selective docking platform for autophagic receptors and adapters. Such adapters have been identified for plastids [45,46] and 26S proteasomes [47], and evidence for selective autophagy in plants has been shown for ER [48] and peroxisomes [49,50].

NBR1 (NEIGHBOR OF BRCA1; also known as *Joka2* in tobacco) [51] was the first selective autophagy receptor identified in plants through domain organization and homology of two mammalian autophagic adapters p62/SQSTM1 and NBR1 [52]. NBR1 plays a role in clearing stress-induced protein aggregates (aggrephagy) and is implicated in responses to multiple stresses, including heat, drought, and salinity [53,54,55]. Additionally, *A. thaliana nbr1* mutants have been shown to accumulate toxic protein aggregates and to be hypersensitive to heat, drought, oxidative, and salt stress [54]. In addition to the aggrephagy, NBR1 contributes to stress tolerance by erasing the heat stress memory through clearance of heat shock-related chaperones [53,54,56]. Recently, *A. thaliana* NBR1 was found to function as a microautophagy receptor for photodamaged chloroplasts exposed to high light independently of the ATG7-ATG8 pathway [57]. Consistent with the role in stress, transcription of wheat *TaNBR1* was upregulated by drought [58]. Thus, ATG8 and NBR1 abundance can inform on autophagic responses to stress.

This work aims to develop phenotyping tools for assessing the activity of autophagy of wheat in response to heat wave conditions simulated by exposure to 40 °C under reduced soil moisture content. We measured protein and transcript abundance for three markers of autophagic activity: ATG8, ATG7, and NBR1 in a panel of 16 phylogenetically distant wheat genotypes adapted to different geographical regions. The stress was applied under greenhouse conditions. The markers were correlated with yield collected in the field trials under heat and drought stress. Changes in ATG8 and NBR1 protein abundance correlated negatively with yield, and changes in *ATG8* gene transcription correlated positively with yield. We conclude that ATG8, ATG7, and NBR1 inform on the heat and drought stress resiliency and can be exploited to develop varieties for arid and hot environments.

## 2. Materials and Methods

### 2.1. Genotypes

A spring wheat NAM population was produced using the semi-dwarf variety “Berkut” and 29 landraces from different geographical zones adapted to a wide range of climates (Table 1) [11]. All progeny and the founder lines were genotyped using the Illumina 90 K iSelect assay and subjected to genotyping by sequencing to produce a genetic map of ~23,000 markers [11]. Each founder line has been re-sequenced using the 107 Mb whole exome capture assay to generate a haplotype map. The common SNP markers shared among the genetically mapped SNPs in the NAM population and a haplotype map were used to project SNPs from the haplotype map to each NAM RIL, creating a dense sequence-based SNP map.

### 2.2. Field Trials

Field trials were performed at WSU Irrigated Agricultural Research and Extension Center, Othello, WA, USA, and WSU Lind Dryland Research Station, Lind, WA, USA, where the average annual precipitation level is below 0.28 m and the maximum day temperature in June can reach above 40 °C. Each genotype was planted in a randomized complete block pattern in 3 replications. Supplemental irrigation was not applied. Plot size was 5.6 m^2^ (1.55 × 3.6 m plot). The Othello location was planted on 24 April 2023 and harvested with a combine on 24 August 2023. The Lind site was planted on 16 March 2023 and harvested on 2 August 2023. One-way ANOVA was used to assess the statistical significance of yield differences between varieties.

### 2.3. Greenhouse Procedures

The plants were grown in growth chambers in a 16/8 h light/dark cycle, 22 °C during the day and 18 °C at night, 60% humidity, and artificial illumination ~400–500 µmol/m^2^/s. Fifteen seedlings were planted in a 1-gallon pot filled with Sun Gro Sunshine Mix #1 soil (Sun Gro Horticulture Distribution Inc., Agawam, MA, USA) supplemented with 3.5 kg/m^3^ Osmocote Plus slow-release fertilizer (The Scotts Co., Marysville, OH, USA). Two pots were set up for each watered control and heat and drought treatment. When plants were 3 weeks old, drought stress was induced by withholding the watering. The volumetric water content (VWC) was measured using a ProCheck Soil Moisture Probe with a 5TC probe (METER Environment, Pullman, WA, USA). Once soil moisture reached 1%, plants were moved to a growth chamber set at 40 °C for 1 h. Immediately after the heat stress, leaf material was collected, frozen in liquid nitrogen, and stored at −80 °C.

### 2.4. Preparation of Antibodies

Polyclonal antibodies were produced according to our published procedure [59,60,61]. A fragment of ATG8 (GenBank AK457482.1; Supplemental Table S1) corresponding to amino acid residues 1 to 116 was amplified by PCR using forward and reverse primers containing Nhe I and Xho I restriction sites, respectively. The PCR fragment was cloned in pGEM-T Easy (Promega, Madison, WI, USA) and verified by sequencing. The fragment was cut from the pGEM-T Easy by digesting with Nhe I and Xho I and cloned into expression vector pET28a cut with NdeI and XhoI. The recombinant protein with the N-terminal His-Tag was produced in *Escherichia coli* strain Rosetta II (Millipore-Sigma, Burlington, MA, USA).

A fragment of ATG7 (GenBank AGW81787.1; Supplemental Table S1) corresponding to amino acid residues 31 to 960 was amplified by PCR and cloned into pDONR207 (Thermo Fisher Scientific, Waltham, MA USA) entry vector using the GateWay system. The fragment was verified by sequencing and cloned into the pGAT4 destination vector. The recombinant ATG7 protein was produced with N-terminal His-Tag fusion in *E. coli* strain Rosetta II.

A fragment of NBR1 (GenBank DQ211935.1; Supplemental Table S1) corresponding to amino acid residues 20 to 462 was amplified by PCR and cloned into pDONR207 (Invitrogen, Waltham, MA, USA) entry vector using GateWay system. The fragment was verified by sequencing and cloned into the pGAT4 destination vector. The recombinant NBR1 protein was produced with N-terminal His-Tag fusion in *E. coli* strain Rosetta II.

Total bacterial protein was extracted using 8 M urea, 50 mM HEPES, 300 mM NaCl, 20 mM Imidazole, and 5 mM Mercaptoethanol, pH 7.0 buffer. Recombinant proteins were purified on a nickel–nitrilotriacetic acid agarose column (Qiagen, Venlo, The Netherlands) and eluted off the column in a 2 M Urea, 50 mM HEPES, 300 mM NaCl, and 250 mM Imidazole, pH 7.0 buffer. Purified protein was dialyzed against PBS supplemented with 20% glycerol overnight at 28 °C.

For the production of ATG7 and NBR1 antibodies in mice, the antigen concentration was adjusted to 1 mg/mL with PBS supplemented with 20% glycerol. A total of 4 boosts were administered over 2 months, each containing 50 μg of recombinant ATG7 or NBR1. The first injection was a 1:1 mixture (by volume) of antigen with Freund’s complete adjuvant (Millipore-Sigma, Burlington, MA, USA), and the subsequent boosts were a 1:1 mixture with Freund’s incomplete adjuvant (Millipore-Sigma, Burlington, MA, USA). Antiserum was collected 10 days after the final boost and tested by immunoblotting against the recombinant antigen protein and total wheat protein extract.

The ATG8 antibody was produced in rabbits. The protein concentration was adjusted to 1 mg/mL, 500 μg of recombinant ATG8 was used for each boost, and a total of 7 boosts occurred over 9 months. For the first injection, recombinant protein was mixed 1:1 with Freund’s complete adjuvant. A total of 25 days after the 1st boost, ATG8 mixed 1:1 with Freund’s incomplete adjuvant was administered, and antiserum was collected 10 days after. The subsequent 5 boosts were mixed 1:1 with Freund’s incomplete adjuvant, and antiserum was collected in the same manner. Altogether, seven bleeds were collected. Each antiserum was tested by immunoblotting against the recombinant ATG8 and total protein extract.

### 2.5. Affinity Purification of Antibody

ATG8 antibody was affinity-purified using ATG8 bound to Pierce™ NHS-Activated Agarose Resin (Thermo Fisher Scientific, Waltham, MA, USA). Recombinant ATG8 protein was expressed and purified as above and concentrated at 2 mg/mL with a spin protein concentrator (Thermo Fisher Scientific, Waltham, MA, USA). ATG8 protein was further purified using a size exclusion column Cytiva™ Superdex 200 Increase 10/300GL column (Millipore-Sigma, Burlington, MA, USA) on an AKTA-FPLC in 4 M Guanidine-Hydrochloride buffered by phosphate, pH 7.2. Fractions containing pure ATG8 were pooled together and concentrated to 1 mg/mL. A total of 300 mg of NHS-activated agarose dry resin was rehydrated with 0.1 M sodium phosphate and 0.15 M sodium chloride, pH 7.2 buffer, leading to a column of ca. 2 mL. A total of 5 mL of ATG8 protein (1 mg/mL) was then added to the resin and incubated at 30 °C for 2 h. The resin was then quenched using 1 M Tris, pH 7.4, and washed in 100 mM Tris and 500 mM NaCl, pH 7.2.

The ATG8 antisera were pooled together, supplemented with 100 μM PMSF, 25 μM Leupeptin, 100 μM Pepstatin A, 50 mM Tris, and 150 mM NaCl, pH 7.4, and then incubated with the ATG8-NHS resin column for 1 h at 4 °C. The resin was washed 3× with 50 mM Tris and 150 mM NaCl, pH 7.4, and bound anti-ATG8 was eluted from the column in 100 mM Glycine-HCl, pH 2.5, supplemented with pH indicator phenol red at 0.5% (*w*/*v*). The eluate was immediately mixed with 1 M Tris, pH 9.0, to adjust the pH to 7.5. Then, bovine serum albumin and sodium azide were added to the final concentration of 5% and 0.05%, respectively. Purified Anti-ATG8 serum was concentrated with 10 kD spin concentrators, aliquoted, flash-frozen in liquid nitrogen, and stored at −80 °C.

### 2.6. Western Blotting

Total protein was extracted from the leaf by grinding the tissue in liquid nitrogen using a mortar and pestle, followed by homogenizing in an extraction buffer (50 mM Tris, pH 7.2, 10 mM EDTA, 10 mM Mercaptoethanol, and proteinase inhibitors 100 μM PMSF, 25 μM Leupeptin, 100 μM Pepstatin A, 1 μM E10, and 1 μM MG132). The debris was removed by centrifugation at 13,000× *g* for 5 min at +4°. The supernatant was collected, mixed 1:1 with 2× SDS-PAGE buffer, and boiled for 3 min. The extracts were separated on a 15% SDS-PAGE gel and transferred onto a Polyvinylidene difluoride (PVDF) membrane (Millipore-Sigma, Burlington, MA, USA).

To prepare the immuno-depleted anti-ATG8, recombinant ATG8 at a final concentration 10 μg/mL was incubated with the primary antibody diluted 1:100 in 1× TBST supplemented with 5% (*w*/*v*) fat-free milk powder at room temperature for 30 min. Then, a PVDF membrane with recombinant ATG8 was cut into strips and washed for 20 min in the milk-TBST. One strip was incubated with primary antibody diluted 1:100 in the same buffer, and another strip was incubated with the depleted primary antibody for 1 h at room temperature. Both strips were washed 3 times for 10 min in TBST and incubated with secondary anti-rabbit horseradish peroxidase conjugates (Jackson ImmunoResearch Laboratories, Inc., West Grove, PA, USA) diluted to 1:2000 for 35 min. Unbound secondary antibodies were washed off in TBST three times for 10 min each. The membranes were developed by ECL reagent (Millipore-Sigma, Burlington, MA, USA), and images were captured using the G:BOX Chemi XT4 Gel Imaging System (Syngene, Frederick, MA, USA).

The same procedure was used to generate immuno-depleted anti-ATG7 and anti-NBR1. ATG7 or NBR1 recombinant proteins were used, and the corresponding antiserum was tested at 1:500 dilution. Anti-mouse horseradish peroxidase conjugate (Jackson ImmunoResearch Laboratories, Inc., West Grove, PA, USA) diluted to 1:2000 was used as the secondary antibody.

Total protein from leaves was extracted as described above. After removing debris by centrifugation, protein concentration in the extracts was measured using Bradford reagent (Bio-Rad, Hercules, CA, USA) and adjusted to 1 mg/mL. Samples were then mixed 1:1 with a 2× SDS-PAGE sample buffer and boiled for 3 min. Each gel well was loaded with 25 μg of total protein. The extracts were run on a 15% SDS-PAGE gel and transferred onto PVDF membranes. The membranes were washed with 1× TBST supplemented with 5% (*w*/*v*) fat-free milk powder for 20 min. The membranes were incubated with the primary antibody, either anti-ATG8 diluted 1:100, anti-ATG7, or anti-NBR1 diluted 1:500 in TBST-milk for 1 h. The membrane was washed and then incubated with the secondary antibodies (either rabbit-HPR or mouse-HRP) at 1:2000 dilution and imaged as described above.

The membranes were washed 3 times for 10 min with deionized water, and then the total protein was stained with colloidal silver or amido black. Total protein abundance on the membranes stained with colloidal silver or amido black and luminescence on the Western blotting images were measured using Fiji ImageJ [62]. The luminescence intensity values were normalized by the protein content on the membrane. We performed two independent stress experiments. The total protein was extracted from two plants of each genotype per experiment. Three membranes were prepared per extract and were treated as technical replicates. The technical replicates were averaged, and the ratio of signal intensity in the extracts from stressed plants to the control was calculated. Four data points were generated per each genotype and treatment. Statistical significance of the difference between the intensity of bands in the control and stress-treated samples was analyzed using Student’s *t*-test relative to the Berkut.

### 2.7. Microsomal Fractionation and Delipidation

Leaves were ground under liquid nitrogen using a mortar and pestle and homogenized with TNIP buffer (50 mM Tris-HCl, pH 8.0, 150 mM NaCl, 1 mM PMSF, and 10 mM Iodoacetamide). The homogenate was filtered through cheesecloth and centrifuged at 2000× *g* for 5 min. The supernatant was collected and centrifuged at 100,000× *g* for 1 h. The pellet was resuspended in the TNIP buffer or a 2× SDS-PAGE buffer.

For the delipidation reaction, the microsomal fraction was resuspended in 0.5% triton X-100 in the TNIP buffer. Total protein was extracted using the above procedure. A 100 μL of total protein extract was incubated with 250 unit/mL^−1^ of *Streptomyces chromofuscus* phospholipase D at 37 °C for 1 h. Reactions were then mixed with a 2× SDS-PAGE sample buffer, ran on a 15% SDS-PAGE gel, and transferred to a PVDF membrane. Membranes were probed with anti-ATG8 as described above.

### 2.8. Immunoprecipitation and Proteomics

Affinity-purified anti-ATG8 was incubated with protein A agarose (Pierce) for 1.5 h on ice, with shaking. The agarose resin was washed with NET-buffer (50 mM Tris-HCl, pH 7.5, 150 mM NaCl, 1 mM EDTA, 0.1% Nonidet P-40, and 0.02% sodium azide) and incubated with total protein extract from leaves prepared as described above, for 1.5 h on ice. The resin was washed with NET-buffer, and the bound protein was eluted with 100 mM Glycine-HCl, pH 2.7. Then, the pH of the eluate was immediately adjusted to pH 7.5 with 1 M Tris, pH 9. The eluate was concentrated down to 100 μL, mixed 1:1 with 2× concentrated SDS-PAGE sample buffer, and boiled for 3 min. The extracts were run on a 15% SDS-PAGE gel, transferred onto a PVDF membrane, and with anti-ATG8 as above. For protein identification, the extracts were separated on 15% SDS-PAGE gel, and bands corresponding to specific molecular weight were excised. Mass spectrometry analysis was performed by the Southern Alberta Mass Spectrometry Center. Peptides were identified using the *T. aestivum* protein database. Predicted proteins were then analyzed using Scaffold 5 software (Proteome Software, Inc., Portland, OR, USA). Resin without antibodies was used as a negative control.

### 2.9. Analysis of Gene Expression by RT-qPCR

Homoeologs for *ATG8* genes [25] were aligned using Clustal Omega [63]. The alignment was used to design RT-qPCR primers to target individual orthologs or conserved regions of several genes (Supplemental Dataset S1; Supplemental Table S1). Total RNA was extracted using the RNeasy plant kit (Qiagen) from leaf material. cDNA was synthesized using a Maxima H Minus First Strand cDNA Synthesis Kit (Thermo Fisher Scientific). RT-qPCR reactions were performed using Fast SYBR™ GreenMaster Mix (Thermo Fisher Scientific) in 96-well plates in a CFX96 thermocycler (Bio-Rad). The genes of interest were normalized to a housekeeping gene ADP-ribosylation factor 2, Genbank: XM_044502292.1 [64].

### 2.10. Phylogenetic Analysis and Structure Prediction

Homoeologs for ATG8 were identified using BLAST with the wheat genome database IWGSC RefSeq v2.1 and clustered based on relative homology scores. Amino acid sequences were downloaded and aligned using the ClustalX version 2.1 software (Supplemental Dataset S2) [65]. The phylodendrograms were constructed in PAUP using the Jackknife method. Bootstrap values were calculated from 1000 iterations. *Saccharomyces cerevisiae* ATG8 was used as the outgroup.

Predicted structures of wheat ATG8 were constructed in the AlphaFold Protein Structure Database [66]. Amino acid sequences for ATG8c-2A (uniport: Q7XY24), ATG8-6A (uniport: A0A3B6NXU7), ATG8l-6B (uniport: A0A3B6PL31), and ATG8m-6D (uniport: A0A3B6QLC9) were blasted in Uniport and imported in AlphaFold. Protein structures were visualized and superimposed using UCSF ChimeraX version 1.7.1 software [67].

### 2.11. RNA-Seq Analysis

RNA-seq data were downloaded from the Gene Expression Omnibus (GEO, http://www.ncbi.nlm.nih.gov/geo/ accessed in 1 August 2021). The database was searched using the keywords “drought stress” AND “species name” [organism]. The datasets were selected according to the following criteria: (1) more than one replicate per treatment; (2) each experiment includes control and drought stress treatments; (3) RNA was extracted from the above-ground organs; (4) drought stress was performed in soil by withholding watering; and (5) the plants were of the wild type (Supplemental Table S2).

The pipeline for RNA-seq data handling followed our established procedure [59]. Briefly, the quality of reads was assessed by FastQC software ver. 0.11.9 (available online: https://www.bioinformatics.babraham.ac.uk/projects/fastqc/, accessed on 20 July 2020). The adapter sequence, low-quality bases (<Q30), and short reads were filtered out using Trimmomatic ver. 0.39 [68]. Hisat2 ver. 2.2.1 aligner was used to map the reads to the reference genomes. *A. thaliana* reads were mapped to the TAIR10 genome (www.arabidopsis.org accessed in 1 August 2021); *Zea mays* reads were mapped the B73v4 genome (https://www.maizegdb.org accessed in 1 August 2021); *Oryza sativa* reads was mapped to the MSU7 genome (http://rice.plantbiology.msu.edu accessed in 1 August 2021); *Sorghum bicolor* reads were mapped to the RTx430 or Sbv3 depending on the experiment (https://phytozome.jgi.doe.gov accessed in 1 August 2021). *Solanum tuberosum* and *Solanum lycopersicum* reads were mapped to the reference genome downloaded from (https://phytozome.jgi.doe.gov accessed in 1 August 2021). Reads were counted by featureCounts software (ver. 1.6.4), part of the Subread package 1.6.2 with default parameters [69].

Deseq2 ver. 3.10 (R package) was used to determine differentially expressed genes between two groups of samples [70]. Genes with at least a |log2-foldchange| > 0 in expression and Benjamini–Hochberg adjusted *p*-value (q-value) < 0.05 were considered as differentially expressed genes (DEGs). Heatmaps were generated with the pheatmap() function (NMF ver. 0.17.6) using the variance stabilizing transformation (VST) values with z-score transformation.

Principal component analysis (PCA) was performed using the prcomp() function. Sample-to-sample distances were calculated based on the VST matrix using the as.dist() function with the Pearson method. Fviz_eig() function built-in factoextra package was used to visualize PC1, PC2, and cos2 values.

The reference sequences of autophagy-related genes were downloaded from Phytozome (https://phytozome-next.jgi.doe.gov/ accessed in 1 August 2021) and Uniport (https://www.uniprot.org/ accessed in 1 August 2021) databases for *S. cerevisiae*, *A. thaliana*, and *O. sativa* using the keyword “Autophagy”. Then, OrthoFinder was used to identify autophagy-related genes in our datasets (Supplemental Dataset S3) [71]. The “hmmbuild” tool was used to construct an HMM profile for each ATG gene. The “hmmsearch” tool was further used to search for the ATGs with 1 × 10^−5^ as the threshold in the *T. aestivum* genome. Putative autophagy-related gene candidates were validated on the interpro database (https://www.ebi.ac.uk/interpro/ accessed in 1 October 2021).

### 2.12. Microscopy

Microscopy and immunocytochemistry were performed following our published procedure [72]. Plants were grown, and the heat stress was applied as above. The flag leaf was cut into 5 mm long segments and immediately frozen in liquid nitrogen. Frozen samples were embedded in ice and then cryo-sectioned at 20 to 50 µm with a Leica CM1860 cryostat (Leica Biosystems, Deer Park, IL, USA). The frozen sections were immediately transferred to an ice-cold fixative consisting of 4% (*w*/*v*) formaldehyde in 50 mM PIPES buffer, pH 6.8. The sections were rinsed three times for 5 min each with 50 mM PIPES, pH 6.8, followed by three rinses of 5 min each in tris-buffered saline tween 20 (TBST) (20 mM Tris, 200 mM NaCl, and 0.1% (*w*/*v*) Tween-20, pH 7.5). Then, the samples were treated with a blocking solution for 1 h at room temperature that consisted of 1% BSA (IgG-free and protease-free; Jackson ImmunoResearch Laboratories, Inc. West Grove, PA, USA) and 1% normal goat serum (Jackson ImmunoResearch Laboratories) in TBST containing 0.02% sodium azide. The sections were treated for one hour at room temperature with anti-AtG8 diluted to 1:100 in the blocking solution. The negative control samples were kept in the blocking solution for an additional hour. Then, all samples were rinsed three times with TBST and incubated for 1 h at room temperature in a solution of goat anti-rabbit IgG secondary antibody conjugated to Cy2 (Jackson ImmunoResearch Laboratories) diluted 1:200 in the blocking solution. The sections were then rinsed in TBST, mounted on glass slides in VECTASHIELD^®^ Antifade Mounting Medium (Vector Laboratories, Newark, CA, USA), and viewed with a Leica SP8 confocal laser scanning microscope equipped with a 40× NA1.3 oil immersion objective. Cy2 was excited at 488 nm, and the emission light was collected between 495 and 540 nm. Each image represents a single optical section of 1 μm collected at the pinhole size 1.

## 3. Results

### 3.1. Characterization of ATG8 Protein Heterogeneity

Probing the total leaf extract from the spring wheat variety Berkut grown under normal conditions with polyclonal anti-ATG8 resulted in the detection of bands of molecular weight 12 and 14 kD (Supplemental Figure S1A). In addition, multiple bands of higher molecular weight were observed with seven bleeds from two rabbits. We performed an immuno-depletion assay to examine the nature of these bands. The ATG8 antigen was incubated with the antibody prior to probing the membrane containing the total protein extract. Although the immuno-depleted serum failed to recognize the lower-molecular-weight bands, the higher-molecular-weight bands persisted (Supplemental Figure S1A). This outcome demonstrates that the antiserum recognizes other proteins in addition to ATG8.

To eliminate the non-specific reactivity of the antiserum, we isolated anti-ATG8 by immuno-affinity chromatography. First, ATG8 recombinant protein was purified using nickel affinity chromatography, followed by a second purification step on a gel-filtration column (Supplemental Figure S1B–D). Purified ATG8 protein was covalently bound to the NHS-agarose resin, and the resin was used for isolation of anti-ATG8. The purified antibody cross-reacted with the lower-molecular-weight bands and still recognized several higher-molecular-weight bands. We tested the specificity of the purified antibody with the immuno-depletion assay. This time, all bands disappeared following the immuno-depletion (Supplemental Figure S1E). Thus, all bands detected with anti-ATG8 contain ATG8 antigen. This outcome demonstrates the heterogeneity of ATG8 proteins under normal growth conditions.

**Figure 1:**
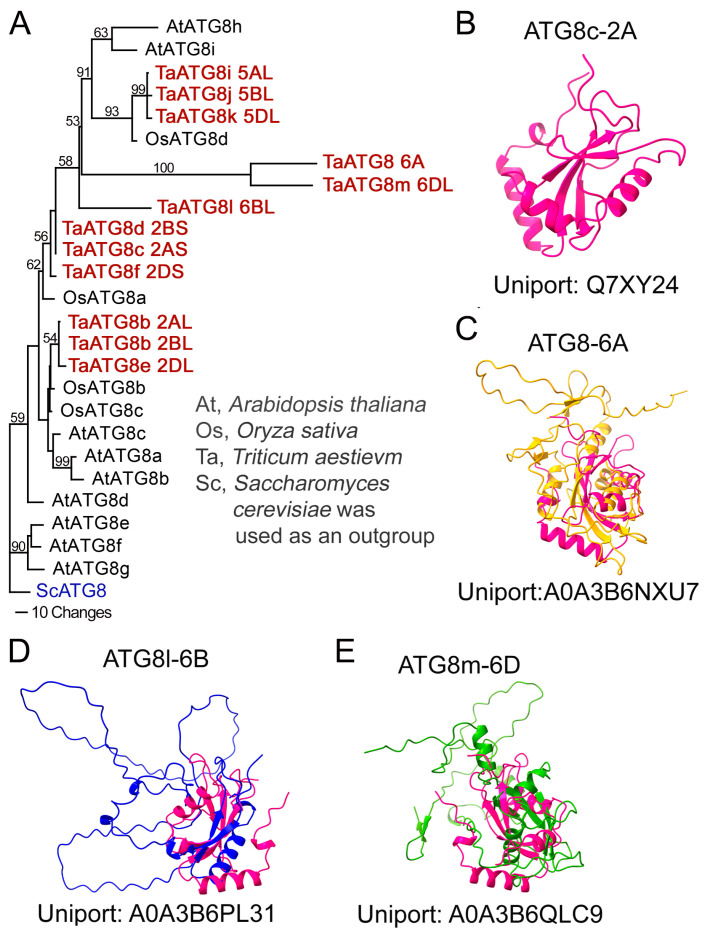
Phylogeny and predicted structure of wheat ATG8. (**A**) Phylodendrogram of ATG8 proteins from *T. aestivum* (Ta; highlighted in red), *A. thaliana* (At), and *O. sativa* (Os). *S. cerevisiae* (Sc; highlighted in blue) ATG8 was used as the outgroup. (**B**) Structure of ATG8c-2A (uniport: Q7XY24) based on AlphaFold prediction. (**C**–**E**) Predicted structures of ATG8 homoeologs on chromosome 6 superimposed with ATG8c-2A (pink). (**C**) ATG8-6A (uniport: A0A3B6NXU7). (**D**) ATG8l-6B (uniport: A0A3B6PL31). (**E**) ATG8m-6D (uniport: A0A3B6QLC9).

Next, we examined the probability that ATG8 heterogeneity originates from the cross-reactivity of anti-ATG8 with paralogs or proteins harboring ATG8-like motifs. The wheat genome has 12 putative ATG8 proteins and no other proteins containing ATG8-like motifs longer than seven amino acids (Supplemental Figure S2). Phylogenetic analysis under stringent conditions (bootstrap value cut-off score above 70%) produces a phylodendrogram with only one clade containing wheat, rice, and *A. thaliana* proteins (Figure 1A). Two wheat paralogs, ATG8-6A and ATG8m-6D, form a distinct clade. Other genes remain unclustered. A lower stringency analysis generated a more complex phylodendrogram (Supplemental Figure S3). Next, wheat ATG8 proteins were analyzed for conserved regions. Only one seven-amino-acid-long peptide was conserved in 10 of the 12 TaATG8 proteins, and no other protein in the NCBI Genbank contained this motif. Thus, additional bands could result from antibodies recognizing ATG8 paralogs but not from cross-reactions with other proteins.

Sequences of ATG8-6A and ATG8m-6D showed conservation with ATG8-6B but lacked the highly conserved ubiquitin-like fold. We visualized the predicted protein structures for all TaATG8 homoeologs on chromosome 6 (Figure 1B–E) and superimposed each of them over the predicted structure of ATG8c-2A. The alignment demonstrated a structural difference in the predicted ubiquitin fold, meaning that ATG8-6A and ATG8m-6D are not canonical ATG8. The predicted size of all ATG8 proteins on chromosome 6 ranges between 22 and 26 kD, while the predicted size of other ATG8 proteins is ~12 kD.

**Figure 2:**
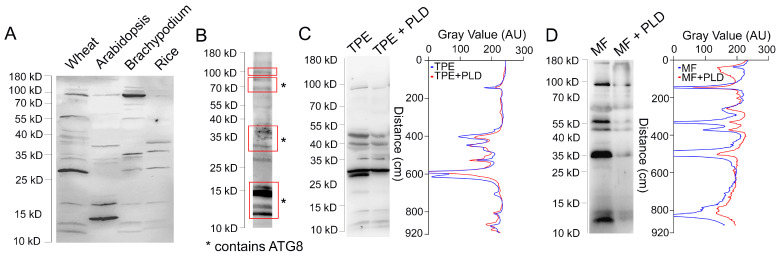
Characterization of ATG8 heterogeneity. (**A**) Western blot of total protein extract from *T.aestivum*, *A. thaliana*, *B. distachyon*, and *O. sativa* using anti-ATG8. (**B**) Western blot of proteins that were immunoprecipitated from total protein extract of Berkut probed with anti-ATG8. Red boxes indicate gel slices that were excised and sent for proteomics analysis. Asterisks (*) denote slices in which ATG8 was detected. (**C**,**D**) Delipidation assay of ATG8. Western blotting and corresponding densitometric plots of total protein extracts (**C**) or microsomal fractions (**D**) from leaves of var. Berkut before and after incubation with Phospholipase D with anti-ATG8. Arrows point peaks that disappear after the delipidation. Bars and numbers indicate the position and corresponding size of molecular weight markers.

To test the conservation of ATG8 heterogeneity in other monocot species, we performed Western blotting with total protein extracts from *A. thaliana*, *Brachypodium distachyon*, and *Oryza sativa* grown under normal conditions (Figure 2A). The 17 and 90 kD species were detected in all species, 12, 14, and 27 kDa species occurred in all monocots, and other bands were not conserved.

Conservation of ATG8 heterogeneity in different species prompted us to obtain a deeper insight into the nature of high-molecular-weight bands. It was possible to immuno-precipitate ATG8 bands from total leaf extract (Figure 2B) and analyze the presence of ATG8 in gel slices corresponding to different ATG8 species by proteomics analysis. The excised gel slices correspond to areas highlighted by red boxes in Figure 2B. ATG8 was found in gel slices corresponding to size 10–17 kD, 30–38 kD, and 65–85 kD but not in the slice corresponding to 95–110 kD.

It has been shown that lipidation with phosphatidylethanolamine contributes to the generation of ATG8 heterogeneity as detected by Western blotting [73]. To examine this possibility, we incubated the total protein extract and microsomal fraction with phospholipase D. After the delipidation reaction, the intensity of the two bands was significantly reduced in both samples (Figure 2C,D). Thus, immuno-affinity-purified ATG8 antibody captures the heterogeneity of ATG8 states, some of which are generated through lipidation.

### 3.2. Impact of Heat and Drought on ATG8 Protein and Transcript Levels

Autophagic responses to heat and drought stress were measured in sixteen genotypes from the spring wheat diversity panel “Elite” (Table 1) [11]. The lines were selected according to their yield in field trials during the 2023 growth season. The trials were performed in a research farm located near Othello and Lind (Washington, DC, USA), where the average precipitation in the 2023 season was 1 mm, and the average temperature was 29 °C (Supplemental Figure S4A). One-way ANOVA analysis was used to compare the differences in yield between LDRC2 and other genotypes in both locations and the average between the locations (Supplemental Figure S4D–F). We selected all genotypes that showed significantly higher yield than LDRC2 (LDRC9, LDRC19, LDRC37, LDRC43, LDRC48, and LDRC74) and several that showed no statistically significant differences genotypes (LDRC2, LDRC10, LDRC33, LDRC42, LDRC65, LDRC5, LDRC15, LDRC16, and LDRC81). Berkut was selected as a control. Selected lines were subjected to a heat wave of 40 °C for 1 h under low soil moisture content. The experiment was repeated twice. In each experiment, we collected leaves from two different plants, and all subsequent experiments were performed with this material.

**Figure 3:**
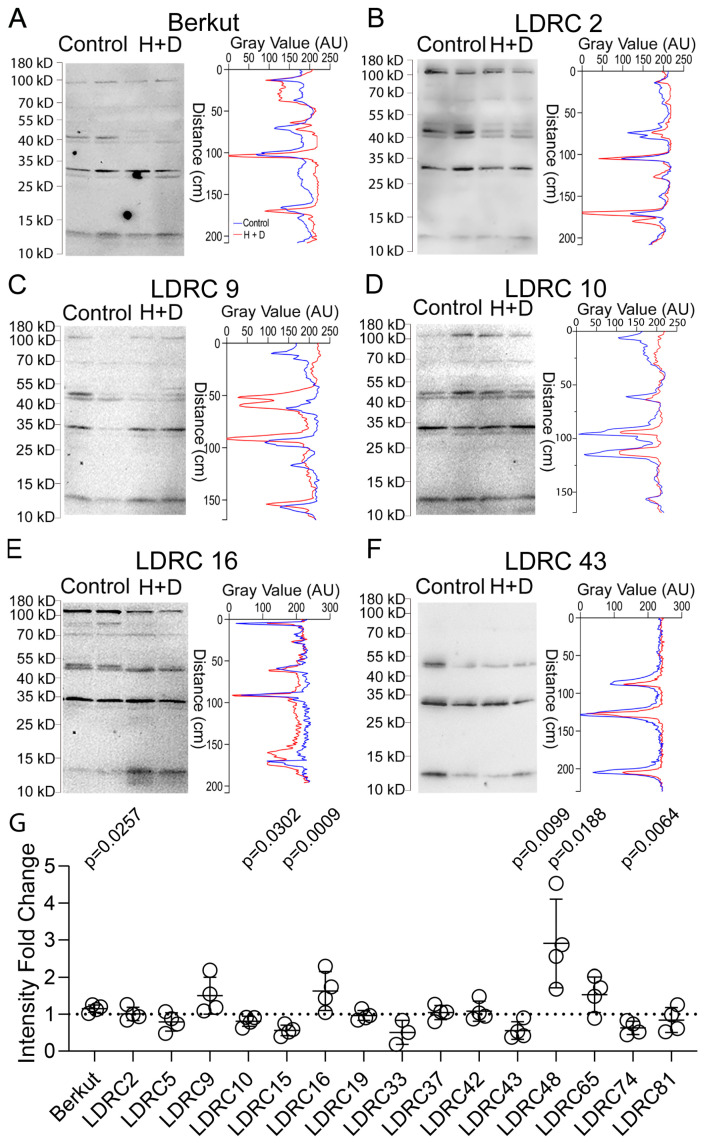
Response of ATG8 bands to heat and drought. (**A**–**F**) Representative images of Western blotting with anti-ATG8 and corresponding densitometric scans of total protein extracts from leaves of control and heat- and drought-stressed (H + D) Berkut plants (**A**), LDRC2 (**B**), LDRC9 (**C**), LDRC10 (**D**), LDRC16 (**E**), LDRC43 (**F**). Bars and numbers indicate the position and size of molecular weight markers. (**G**) Fold change of cumulative ATG8 bands intensity in extracts from heat and drought stress material relative to the control. *p*-values represent statistical differences for Student’s *t*-test at 95% confidence (*n* = 4).

Western blotting with anti-ATG8 revealed genotype-specific patterns of ATG8 bands that were seemingly different under stress treatment (Figure 3A–F; Supplemental Figure S5). Quantification of the cumulative intensity of 15 kD, 30 kD, and 70 kD bands in each lane of the heat and drought stress extracts relative to the control revealed three patterns (Figure 3G): (1) no change in relative ATG8 abundance (Berkut, LDRC2, LDRC19, LDRC37, LDRC42); (2) increase in ATG8 abundance (LDRC9, LDRC16, LDRC48, LDRC65); and (3) decrease in ATG8 abundance (LDRC 5, LDRC10, LDRC15, LDRC33, LDRC43, LDRC74, LDRC81). The intensity of the Rubisco band was used for the normalization of protein loading.

**Figure 4:**
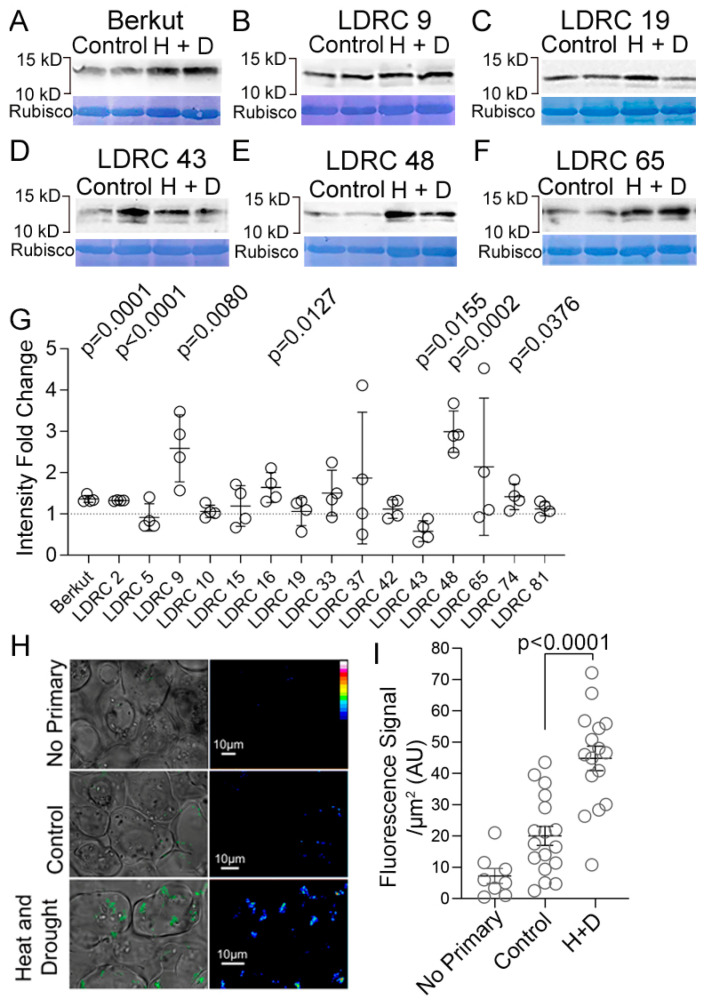
Impact of heat and drought stress on the abundance of ATG8. (**A**–**F**) Western blotting with anti-ATG8 of total protein extracts from leaves of control and heat- and drought-stressed (H + D) plants of Berkut (**A**), LDRC9 (**B**), LDRC 19 (**C**), LDRC 43 (**D**), LDRC 48 (**E**), LDRC 65 (**F**). Bars and numbers indicate the position and corresponding size of molecular weight markers. Amido black staining of the corresponding membrane showing Rubisco protein. (**G**) Fold change of ATG8 protein abundance in response to heat and drought stress relative to the control. *p*-values represent statistical differences between control and stress treatments for Student’s *t*-test at 95% confidence (*n* = 4, two different plants in two independent experiments). (**H**) Representative images showing immunostaining of ATG8 in control or heat and drought-treated leaves of LDRC48. Each image is a single 1 mm thick optical section. Scale bar, 10 mm. (**I**) Average fluorescence signal of individual ATG8 puncta in mesophyll cells of control or heat and drought-treated leaves. *p*-values represent statistical differences for Student’s *t*-test at 95% confidence (*n* = 8, 17, or 16 puncta for negative control, watered or stressed plants, at least 5 cells per each sample).

Next, we measured the abundance of 12–15 kD ATG8 bands under control and heat and drought stress (Figure 4A–F). Generally, there were three types of responses (Figure 4G): (1) increase in relative ATG8 abundance (Berkut, LDRC2, LDRC9, LDRC16, LDRC48, LDRC74); (2) decrease in relative ATG8 abundance (LDRC 43); (3) no change (LDRC10, LDRC42, LDRC81). In LDRC5, LDRC15, LDRC19, LDRC33, LDRC37, LDRC65 the response was inconsistent. To test the correlation between ATG8 abundance and the formation of autophagosomes, we stained autophagosomes on the transverse leaf sections of LDRC48 under control and stress. This genotype was selected for higher ATG8 protein abundance under stress conditions relative to the control. We reasoned that this genotype can be used for correlating ATG8 protein abundance and the accumulation of autophagosomes. Anti-ATG8 serum stained puncta in both the control and the stressed leaves, though the intensity of staining in the control was weaker relative to the stress-treated samples on images recorded under identical microscope settings (Figure 4H). Quantification revealed that the average fluorescence signal of the ATG8-positive puncta in LDRC48 under heat and drought was significantly higher than the control (Figure 4I). Thus, an increase in ATG8 protein abundance correlates with a greater number of autophagosomes in cells.

**Figure 5:**
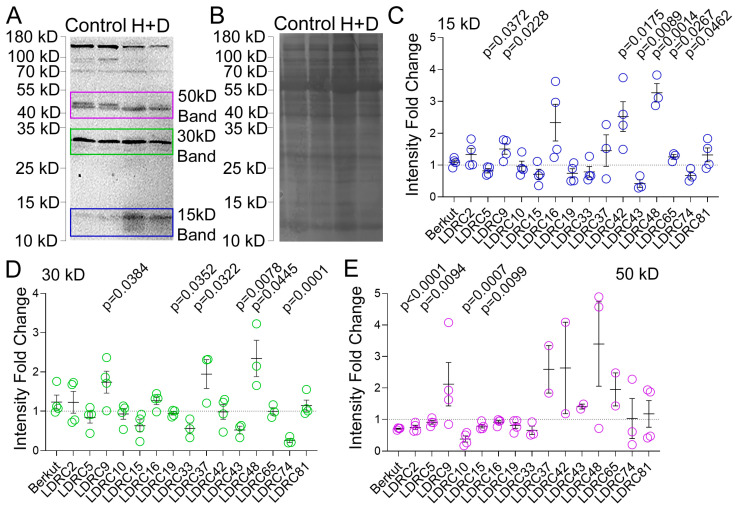
Impact of heat and drought stress on different ATG8 bands. (**A**,**B**) A representative Western blot (**A**) and corresponding colloidal-silver stained membrane (**B**) of total protein extract from leaves of control and stressed LDRC16 with anti-ATG8. The intensity of bands corresponding to approximately 15 kD, 30 kD, and 50 kD denoted by the rectangles were used for quantification in panels (**C**–**E**). Bars and numbers indicate the position and corresponding size of molecular weight markers. Fold change of 15 kD (**C**), 30 kD (**D**), or 50 kD (**E**) ATG8 bands abundance in the heat- and drought-stressed plants relative to the control. *p*-values represent statistical differences for Student’s *t*-test at 95% confidence (*n* = 4, two different plants in two independent experiments).

As ATG8 protein was also detected in the gel slices corresponding to ~30 kD and ~50 kD bands, we compared the impact of heat stress on the abundance of these bands with the abundance of 15 kDa bands. The regions used for measuring the intensity of bands are shown in Figure 5A. In most genotypes, the response of the bands was synchronous. The intensity of all bands increased or was not affected in LDRC9, LDRC37, LDRC42, LDRC48, LDRC65, and LDRC81 (Figure 5B–D). In other varieties, the intensity of all bands decreased: LDRC5, LDRC10, LDRC15, LDRC19, LDRC33, and LDRC74. In four varieties, Berkut, LDRC2, LDRC16, and LDRC43, the changes of bands varied. For example, in LDRC16, the intensity of the 15 and 30 kD bands increased, whereas the intensity of the 50 kD decreased. We found that the variability of the 30 kD band between biological replicates was lower than the variability of the 15 kD and 50 kD bands. The 30 kD band was, on average, lower in intensity in the population compared to 15 kD and 50 kD.

**Figure 6:**
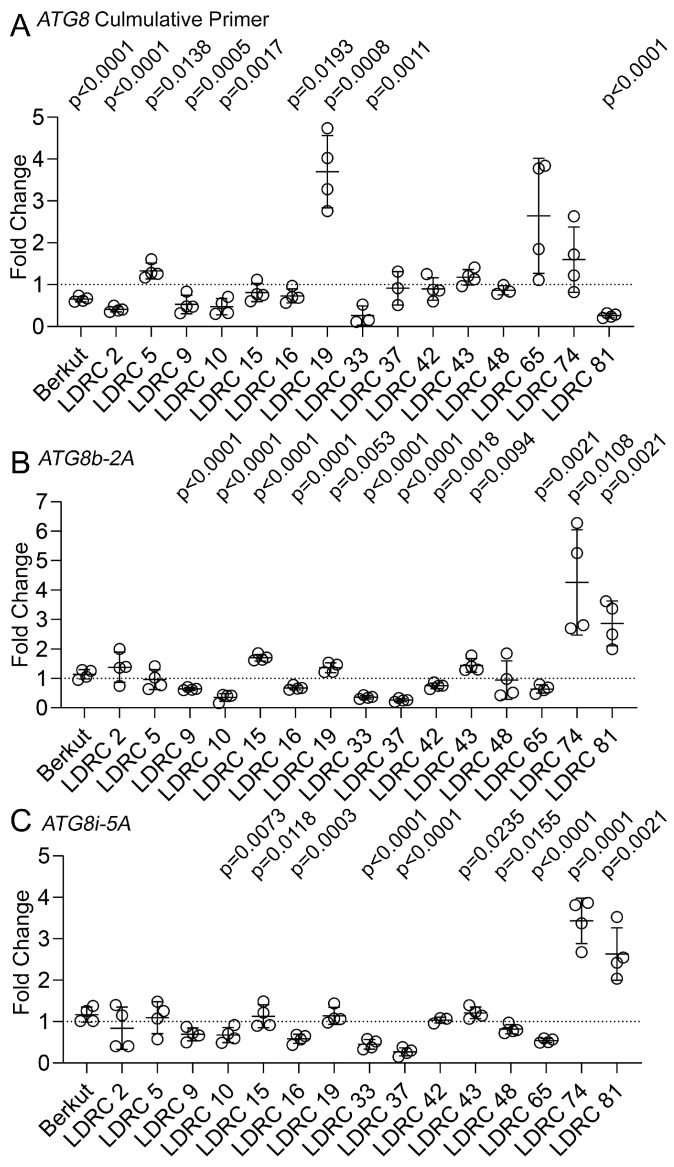
Impact of heat and drought stress on *ATG8* transcription. (**A**) Fold change of cumulative transcription level of *ATG8c-2A*, *ATG8f-2D*, *ATG8d-2B*, and *ATG8l-6B* in response to heat and drought stress relative to control. Student’s *t*-test at 95% confidence (*n* = 4, two different plants in two independent experiments). (**B**,**C**) Fold change of *ATG8b-2A* (**B**) or *ATG8i-5A* (**C**) transcription level in response to heat and drought stress relative to control. ADP-ribosylation factor 2 was used as a housekeeping gene for the normalization of the transcript level (Genebank: XM_044502292.1; [64]) (*n* = 4, two different plants in two independent experiments). Student’s *t*-test at 95% confidence (*n* = 4, two different plants in two independent experiments).

To measure the impact of heat and drought stress on the transcription of *ATG8* genes, we designed a pair of universal primers capable of simultaneous amplification of *ATG8d-2B*, *ATG8c-2A*, *ATG8f-2D*, and *ATG8l-6B2* and two pairs of homoeolog-specific primers for *ATG8b-2A* and *ATG8i-5A* (Supplemental Table S1). At least one of the ATG8 transcripts was upregulated in response to heat and drought stress relative to the control in genotypes LDRC5, LDRC15, LDRC19, LDRC65, LDRC74, and LDRC81. The rest of the genotypes maintained steady transcription levels for all *ATG8* primer pairs (Figure 6).

### 3.3. Impact of Drought Stress on Transcription of Autophagy Genes in Diverse Species

To evaluate the conservation of autophagic responses to stress in other species, we performed transcriptomic analyses using published RNA-seq datasets. We selected drought because it was the most represented stress in the Gene Expression Omnibus (GEO) repository for a broad range of species. Eleven GEO datasets were selected for *A. thaliana*, *O. sativa*, *Z. mays*, *S. bicolor*, *S. tuberosum*, and *S. lycopersicum* (Supplemental Table S2). In the selected experiments, RNAs were extracted from stressed and non-stressed leaves. Each genotype in the same experiment was considered as a separate dataset for differential gene expression analysis. The RNA-seq datasets were subjected to quality control. Low-quality reads (1–20%) were filtered out using the Trimmomatic application. Then, the reads were aligned to the respective reference genome. The alignment rate of the reads was around 80–95% (Supplemental Table S3). The number of reads was estimated using the reads mapped to each predicted gene model.

To determine the similarity of sample replicates and groups, principal component analysis (PCA) was performed using VST values for each dataset. Each dataset was grouped into species, and combined VST values were used in the analysis. The first 2000 most upregulated and downregulated genes were used in PCA analysis (Supplemental Figure S6). Datasets showing high variance among biological replicates were excluded from the analysis. For example, the variance of biological replicates for the *B. distachyon* dataset (GEO accession#GSE126992 and Bioproject#PRJNA360513) and *Panicum virgatum* dataset (GEO accession#GSE132772) failed the PCA analysis.

Differential expression analyses of drought-stressed vs. control samples for each comparison group were performed. Supplemental Dataset S4 contains *p*-values and *p*-adjusted values of DESeq2 analyses. The number of up- and downregulated differentially expressed genes (DEGs) for each analysis, along with the number of predicted and active genes, is summarized in Supplemental Table S4. Genes with higher log2 (fold changes) than zero were considered as differentially expressed. VST values of raw counts were transformed into z-scores to construct the heatmaps. All differentially expressed autophagy genes are shown in Supplemental Dataset S5, and the outcomes are summarized in Supplemental Figure S7. Drought caused upregulation of *ATG8* family members in both dicot and monocot species; *ATG3* and *ATG7* were upregulated mostly in monocots, and *ATG4* and *ATG18* were upregulated mostly in dicots (Supplemental Tables S5 and S6). Hence, *ATG3* and *ATG7* could serve as markers of autophagic activity in wheat.

**Figure 7:**
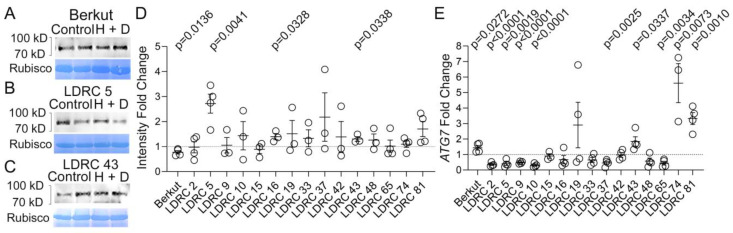
Impact of heat and drought on ATG7. (**A**–**C**) Western blotting with anti-ATG7 of total protein extracts from leaves of control and heat- and drought-stressed (H + D) Berkut (**A**), LDRC5 (**B**), LDRC43 (**C**). Bars and numbers indicate the position and size of molecular weight markers. Corresponding membrane stained with amido black shows Rubisco protein in each lane. Intensity of the Rubisco band was used for the normalization of the signal on the Western blotting. (**D**) Fold change of ATG7 protein abundance in response to heat and drought stress relative to the control. *p*-values represent statistical differences between control and stress treatments for Student’s *t*-test at 95% confidence (*n* = 4, two different plants in two independent experiments). (**E**) Fold change of *ATG7* transcript abundance in response to heat and drought stress relative to the control. ADP-ribosylation factor 2 (Genebank: XM_044502292.1; [64]) was used as a housekeeping gene for the normalization of RT-qPCR values. *p*-values represent statistical differences between control and stress treatments for Student’s *t*-test at 95% confidence (*n* = 4, two different plants in two independent experiments).

As both ATG3 and ATG7 function together in the pathway responsible for the lipidation of ATG8, we only examined the response of ATG7 to heat and drought stress. Polyclonal antibodies against wheat ATG7 were produced in mice. An immuno-depletion assay with ATG7 antigen confirmed that the antibodies specifically recognize the ATG7 band corresponding to ca. 90 kD (Supplemental Figure S8A). The impact of heat and drought stress on ATG7 protein abundance was measured in 16 genotypes (Figure 7A–D; Supplemental Figure S8B). In all genotypes, anti-ATG7 cross-reacted with a single band. Overall, heat and drought stress caused higher ATG7 abundance in LDRC5, LDRC16, and LDRC43 and lower ATG7 abundance in Berkut (Figure 7A–D). Transcription of *ATG7* was upregulated in Berkut, LDRC43, LDRC74, and LDRC81 and downregulated in LDRC2, LDRC5, LDRC9, LDRC10, LDRC37, LDRC43, and LDRC65 (Figure 7E).

### 3.4. Impact of Heat and Drought on NBR1 Protein and Transcript Levels

**Figure 8:**
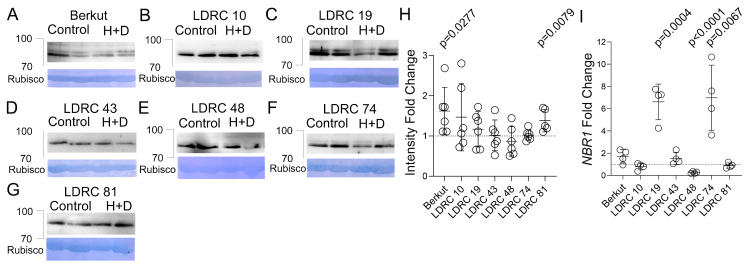
Impact of heat and drought stress on NBR1. (**A**–**G**) Western blotting with anti-NBR1 of total protein extracts from leaves of well-watered (control) or heat- and drought-stressed (H + D) genotypes. Bars and numbers indicate the position and corresponding size of molecular weight markers. Amido black staining of the corresponding Western blotting membrane shows Rubisco protein. (**H**) Fold change of NBR1 protein abundance in response to heat and drought stress relative to the control. *p*-values represent statistical differences between control and stress treatments for Student’s *t*-test at 95% confidence (*n* = 4, two different plants in two independent experiments). (**I**) Fold change of *NBR1* transcript abundance in response to heat and drought stress relative to the control. ADP-ribosylation factor 2 (Genebank: XM_044502292.1 [64]) was used as a housekeeping gene for the normalization of RT-qPCR values. *p*-values represent statistical differences between control and stress treatments for Student’s *t*-test at 95% confidence (*n* = 4, two different plants in two independent experiments).

Heat stress causes misfolding and damaging of proteins, which could cause cytotoxicity. The selective autophagy receptor NBR1 plays a role in targeting protein aggregates for autophagy [53,54]. NBR1 interacts with ATG8 and degrades together with the autophagosome in the vacuole. Therefore, activation of autophagy is expected to increase *NBR1* transcript and decrease or cause no changes in NBR1 protein abundance. We examined NBR1 protein levels in seven varieties that showed different responses of ATG8 to stress. Polyclonal anti-NBR1 was generated in mice. An immuno-depletion assay with NBR1 antigen confirmed that the antibodies specifically recognize the NBR1 band corresponding to ca. 90 kD (Supplemental Figure S9). In some genotypes, the antibody detected a single band, and in others, a doublet (Figure 8A–G). Heat and drought stress did not affect the band pattern and caused an increase in NBR1 abundance only in Berkut and LDRC81. The abundance of NBR1 was not affected in other genotypes. The wheat genome has three homoeologs of *NBR1*. We designed a pair of primers that targeted all of them and measured the impact of heat and drought stress on the transcription of *NBR1* by RT-qPCR. Transcription of *NBR1* was upregulated in Berkut, LDRC19, LDRC43, and LDRC74 and downregulated in LDRC48 (Figure 8I).

### 3.5. Autophagy Markers Correlate with Yield

**Figure 9:**
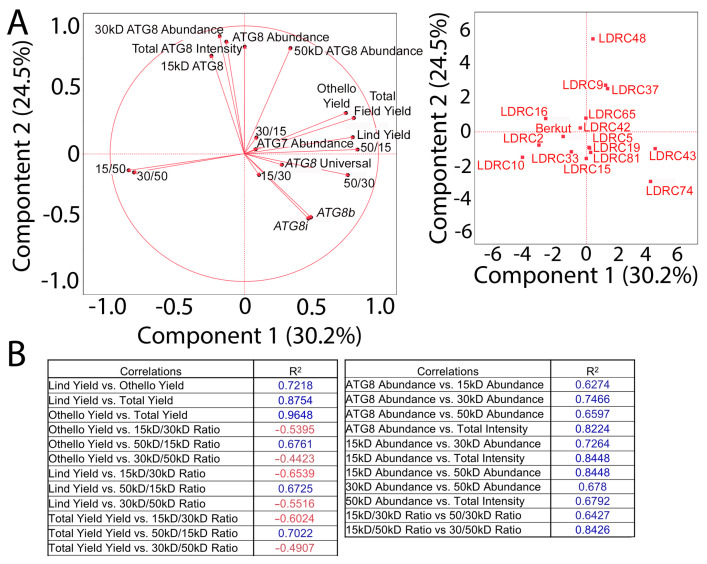
Relationship between yield and autophagy parameters. (**A**) Principal component analysis of yield, ATG8 parameters, and ATG7 for all genotypes. (**B**) R^2^ values for principal component analysis in (**A**). Blue font denotes a positive correlation and red denotes a negative correlation between the values.

We examined the correlation between changes in autophagy markers and yield in each field trial individually and the average yield values. Neither the abundance of 15 kD, 30 kD, 50 kD ATG8 isoforms from one membrane nor the abundance of ATG7 band correlated with yield parameters in the population of 16 genotypes (Figure 9A,B). However, the ratios of 15 kD/30 kD and 30 kD/50 kD bands of ATG8 correlated negatively with yield (R^2^ = −0.5395 and −0.4423, respectively). The abundance of ATG8 isoforms correlated positively with each other (Figure 9B).

**Figure 10:**
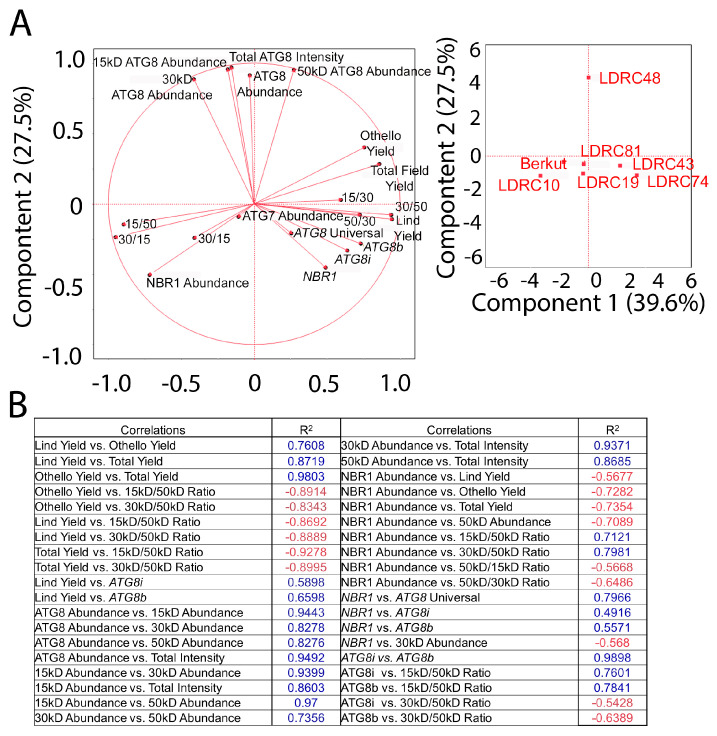
Relationship between yield, autophagy, and NBR1 parameters. (**A**) Principal component analysis of yield ATG8 parameters, ATG7, and NBR1 parameters for 7 genotypes. (**B**) R^2^ values for the principal component analysis in (**A**). Blue color denotes a positive correlation and red denotes a negative correlation between the values.

*NBR1* transcript and NBR1 protein abundance, and *ATG8i* and *ATG8b* transcript abundance correlated positively with yield in the Lind trial with all seven genotypes selected for analysis (R^2^ = 0.5898 and 0.6598, respectively; Figure 8 and Figure 10A,B). NBR1 abundance correlated negatively with yield in both the Lind and Othello trials (R^2^ = −0.5677 and −0.7354, respectively), whereas *NBR1* correlated positively with an abundance of all *ATG8* transcripts (R^2^ = 0.7966, 0.4916, and 0.5571, respectively). *ATG8b* and *ATG8i* abundance correlated positively with each other (R^2^ = 0.9898). The ratios of 15 kD/50 kD and 30 kD/50 kD ATG8 bands correlated negatively with yield.

## 4. Discussion

Recycling of dysfunctional cellular components plays a key role in both development and stress [26,74,75]. Therefore, many cellular mechanisms of adaptation to harsh environmental conditions will rely on autophagy. Here, we analyzed heat and drought responses of autophagic markers ATG8, ATG7, and NBR1 in a genetically diverse panel of stress-tolerant and stress-susceptible wheat genotypes.

### 4.1. Heterogeneity of ATG8 and NBR1

One important discovery of our study is the high heterogeneity of ATG8 proteins in monocots, including wheat. It has been shown that lipidation of ATG8 generates an additional isoform that runs at ~15 kD on the SDS-PAGE gels [73,76]. However, in addition to nascent ATG8 and ATG8-PE isoforms, antibodies against ATG8 cross-react with multiple bands running below ~15 kD in *A. thaliana* [73,77,78]. Our immunoaffinity-purified antibody against wheat ATG8 cross-reacts with multiple bands ranging between 12 and 90 kD. A similar range of ATG8 bands was detected with anti-ATG8 in *O. sativa* and *B. distachyon*. The fact that proteomics analysis identified ATG8 in all but the 90 kD bands suggests that higher-molecular-weight isoforms represent ATG8 species or ATG8 conjugates. The reason for the absence of ATG8 in the ~90 kD band could be explained by a low abundance of ATG8 in this material and technical challenges with the identification of ATG8 peptides. A similar size range of bands was detected in total protein extracts from *A. thaliana* using commercially available ATG8 antibodies [79] and also with our immuno-affinity-purified antibodies. Thus, ATG8 proteins encompass heterogeneous isoforms in both monocot and dicot species.

The size heterogeneity of ATG8 bands could have multiple origins. One reason may be lipidation. Consistent with this hypothesis, some heterogeneity of ATG8 was lost following treatment with lipase. However, even after the delipidation reaction, the pattern of ATG8 bands remained complex. Other post-translational modifications could also contribute to changing the size of nascent ATG8, e.g., it is known that ATG8 undergoes phosphorylation, ubiquitination, and acetylation in mammalian cells [80,81].

Second, the sequence diversity of ATG8 in the allohexaploid wheat genome contains 12 ATG8 paralogs, of which 9 are evolutionarily conserved. Three ATG8 homoeologs on chromosome 6 contain unique intrinsically disordered regions. Based on the phylogenetic analysis, these homoeologs are wheat-specific with a predicted molecular weight of 28 kD. Hence, the ~30 kD bands on the Western blotting in Figure 1A could correspond to these proteins.

Third is the homo- or hetero-oligomerization of ATG8 and covalent binding to the ligands (ATG8ation). For example, it has been shown that ATG8 can dimerize in yeast cells [82]. ATG8-ATG3 conjugates in *A. thaliana* run on an SDS-PAGE gel between ~75 and ~50 kD [73,76]. Recently, ATG8 was shown to interact with CLATHRIN LIGHT CHAIN 2 (CLC2) during Golgi remodeling after heat stress in *A. thaliana* [83]. Two *A. thaliana* paralogs, ATG8a and ATG8i, interact with the ESCRT component FREE1 to regulate autophagosome closure [84].

Interestingly, the pattern of ATG8 bands changes in response to stress. This fact suggests that each of the above mechanisms responsible for ATG8 heterogeneity would not only alter the size but also generate ATG8 forms with specialized functions. Functional diversity and different localization were shown for two ATG8 subfamilies in mammalian cells: MAP1LC3 (microtubule-associated protein 1 light chain 3, hereafter referred to as LC3) and GABARAP (γ-aminobutyric acid receptor-associated protein). The LC3 subfamily primarily functions in autophagophore elongation, whereas GABARAP functions in autophagosome–lysosome fusion [85]. Additionally, mammalian ATG8 paralogs participate in other processes besides autophagy. For example, LC3C is involved in COPII-dependent ER export [86], and GATE-16 (GABARAPL2/ATG8) functions in intra-Golgi trafficking [87].

Plant ATG8 paralogs also show functional diversity. For example, all *A. thaliana* ATG8, with the exception of ATG8h and ATG8i, are cleaved by ATG4 [73]. Analysis of ATG8 transcription patterns supports the hypothesis of functional specialization. For example, GUS-reporter assays demonstrated that out of 5 *ATG8* family members (*ATG8a*, *ATG8c*, *ATG8f*, *ATG8e*, and *ATG8h*), only *ATG8a* and *ATG8h* were upregulated in response to sugar starvation [88]. The five maize *ATG8* are expressed in multiple tissues, including shoot apex, seedlings and leaves (L4, L3, and L2 order of appearance), non-pollinated ears, and tassels [78]. However, the expression level was different: *ZmATG8c* had higher expression in the shoot apex, and *ZmATG8d* was expressed in older leaves [78].

Analysis of other proteins revealed a lack of heterogeneity in the case of ATG7 and genotype-specific heterogeneity in the case of NBR1. Of the seven genotypes analyzed, two showed distinguishable NBR1 double bands in all biological replicates. The upper band may originate from a post-translational modification such as ubiquitination [55,89]. While the heterogeneity of ATG8 was affected by heat and drought stress, the pattern of NBR1 bands remained similar under all growth conditions. Determining the functional significance of NBR1 heterogeneity needs further analysis.

### 4.2. Impact of Heat and Drought Stress on ATG8

As autophagy encompasses the turnover of proteins that are associated with the autophagosomes, higher autophagic activity should be accompanied by reduced or constant ATG8 protein abundance. Sustaining ATG8 production under elevated autophagic flux would require higher transcription of ATG8. Many publications show transcriptional upregulation of ATG8 and other autophagic proteins in response to stress. *ATG1*, *ATG4*, *ATG5*, *ATG8a*, and *ATG18b* were upregulated during drought stress in *Medicago truncatula* [38]. In tomato, eighteen autophagy genes, including *ATG1*, *ATG3*, *ATG7*, *ATG8*, and *ATG9*, were upregulated by drought stress [90] and *ATG5*, *ATG7*, and *NBR1* were upregulated by 45 °C heat stress [39]. *ATG8* was upregulated in both roots and leaves during osmotic stress in *Triticum dicoccoides* [36]. It was shown that the ethylene response factor, *ERF5*, induced by drought stress, binds directly to *ATG8d* in tomato to increase both gene transcription and autophagic activity [91].

Using publicly available RNA-seq datasets, we found that drought causes upregulation of *ATG8* transcript in both dicots and monocots, whereas *ATG3* and *ATG7* were consistently upregulated in three monocots species, *O. sativa*, *Z. mays*, and *S. bicolor*, but not in dicot species *A. thaliana*, *S. tuberosum*, and *S. lycopersicum*. Analysis of *ATG8* transcription in the genetic diversity panel using three pairs of primers revealed that the level of at least one *ATG8* transcript was upregulated in eight of sixteen genotypes. Only one genotype, LDRC74, showed upregulation of genes detected by all three pairs of ATG8 primers in response to heat and drought stress. ATG8 transcription was reduced or not affected in the remaining eight genotypes. ATG8 total protein level was reduced or remained constant in LDRC19, LDRC43, LDRC74, and LDRC81. This outcome is consistent with the upregulation of autophagy in four of sixteen genotypes. Providing both *ATG8* transcripts and ATG8 protein abundance were measured accurately under our experimental conditions and both parameters correlate with autophagic activity, these data demonstrate that activation of autophagy may not be the default response to heat and drought in spring wheat.

Heat and drought stress altered ATG8 heterogeneity in many genotypes. The reduction in total ATG8 protein abundance in LDRC19, LDRC43, LDRC74, and LDRC81 was accompanied by a reduction in the 30 kD band. However, the abundance of 15 kD bands in LDRC74 increased in response to heat and drought stress. The abundance of 30 kD and 50 kD bands increased in LDRC9 and LDRC37, while the abundance of 50 kD bands increased only in LDRC43 and LDRC65 and decreased in LDRC10. These data indicate that ATG8 isoforms play different roles in the stress response.

The 30 kD and 50 kD bands changed in a genotype- and treatment-specific manner. However, these changes were not consistent among the biological replicates and treatments. For example, two bands at 30 kD were detected in Berkut, LDRC10, LDRC19, LDRC65, and LDRC74 under both control and stress treatments. Furthermore, two 50 kD bands were detected in LDRC10 under both conditions, whereas in Berkut, LDRC16, LDRC33, LDRC65, and LDRC81, the 50 kD doublet appeared only under control conditions. Under heat and drought stress, there were three or one 50 kD band in Berkut and three or two bands in LDRC9. Three 50 kD bands in LDRC2 persisted under heat and drought stress but at lower abundance. Variable heterogeneity of the 30 kD and 50 kD bands under control and stress conditions suggest functional specialization of individual ATG8 isoforms.

### 4.3. Correlation between ATG8, ATG7, and NBR1 Abundance and Yield

Multiple pieces of evidence highlight the contribution of autophagy to stress tolerance. For example, overexpression of *ATG10* in apples enhances salt stress tolerance [92], overexpression of *ATG3b* in *A. thaliana* leads to both salt and osmotic stress tolerance [37], and overexpression of *ATG5* or *ATG7* in *A. thaliana* promotes ATG8 lipidation, autophagosome formation, and autophagic activity [43]. The latter transgenic lines are more resistant to oxidative stress (treatment with methyl viologen) and necrotrophic pathogens. *ATG2* and *ATG7* also contribute to salt stress tolerance in wheat through suppression of salt-induced programmed cell death [93]. In *O. sativa*, ATG10 plays a role in salt tolerance and resistance to methyl viologen [29], whereas ATG6 contributes to heat, cold, and drought stress responses [94]. In our experiments, parameters derived from the heterogeneity of ATG8, such as the ratios between ~15 kD and ~30 kD and ~30 kD and ~50 kD bands, correlated negatively with the yield and could be used as markers of stress resiliency.

Heat stress is known to cause protein misfolding and aggregation [54], which are recycled through a specific type of autophagy known as aggrephagy [53,55]. NBR1 acts as a cargo receptor for protein aggregates and becomes recruited to the autophagosome through interaction with ATG8 [54]. The importance of NBR1 for stress tolerance is supported by several studies. First, *A. thaliana nbr1* mutant is hypersensitive to heat stress and oxidative stress (methyl viologen) [54]. Second, overexpression of *NBR1* results in greater UV-B and heat stress tolerance in *A. thaliana* [95], and in a lower abundance of reactive oxygen species under salt stress, higher transcription of *ATG8*, greater autophagosome abundance, and reduced accumulation of insoluble proteins under salt stress in poplar [96].

As an autophagosome cargo receptor, NBR1 degrades in the vacuole together with ATG8 and the autophagosome. Hence, higher autophagic activity should be accompanied by upregulation of *NBR1* transcription and constant or lower NBR1 protein levels. Four out of seven genotypes show this type of response: upregulation of *NBR1* transcription was accompanied by constant protein abundance in LDRC19, LDRC43, and LDRC74; constant transcription level of *NBR1* was accompanied by reduced protein abundance in LDRC48. Changes in NBR1 protein abundance negatively correlated with yield under heat and drought stress in both locations. Thus, NBR1 abundance could be used as a marker of heat and drought stress resilience.

## 5. Conclusions

Although autophagy offers enormous potential for improving crop resilience to abiotic and biotic stress, exploiting this trait in breeding programs is hindered by the lack of phenotyping tools. Robust phenotyping of autophagy would enable the identification of corresponding genetic markers that could be used in genomic selection. Application of three common markers for assessing autophagic activity, ATG7, ATG8, and NBR1, under heat and drought stress in genetically diverse wheat genotypes harnessed several important conclusions.

First, changes in ATG8 and NBR1 protein and transcript abundance in response to heat and drought stress in LDRC19, LDRC43, and LDRC74 are consistent with upregulation of autophagy. These genotypes belong to distant phylogenetic clades and originate from different regions: LDRC19 from Uruguay, LDRC43 from Mozambique, and LDRC74 from Afghanistan. All three genotypes were in the high-yielding group. This outcome means that the higher autophagic activity trait was selected independently by several breeding programs in different geographical locations.

Second, the abundance of ATG8 and NBR1 could be used to assess the autophagic response to stress. Simultaneous measuring of NBR1 and ATG8 abundance increases the accuracy of the prediction. The abundance of ATG7 shows limited variability and poor correlation with yield. This parameter needs further development before using in phenotyping autophagy.

Third, no changes of ATG8 protein or transcript abundance under heat and drought stress in several genotypes suggest a lack of autophagic response. For example, the accumulation of ATG8 protein in LDRC9 and LDRC48 was accompanied by steady *ATG8* transcription. In LDRC10, ATG8 protein abundance remained constant after the stress and *ATG8* transcripts were downregulated. Genotypes LDRC2, LDRC33, and LDRC37 lack changes in both ATG8 protein and *ATG8* transcript abundance in response to stress. Constant abundance of ATG8 was accompanied by downregulation of at least one *ATG8* transcript in Berkut, LDRC9, LDRC16, LDRC42, LDRC48, and LDRC65. Yet, LDRC48 and LDRC9 were among the high-yielding genotypes. Hence, autophagy could be dispensable for stress tolerance.

Suppression of autophagy and accumulation of autophagy proteins under stress could be a common outcome. For example, accumulation of autophagosomes and downregulation of *ATG8c* under heat stress was reported in pepper [40], and accumulation of autophagosomes correlated with accumulation of LC3-II (ATG8) response to aggregation-prone proteins mHTT and alpha-synuclein in mammalian cells [97]. The low frequency of genotypes that activate autophagy in our population could be explained by negative selection in some geographical locations due to the pressure from another environmental factor, e.g., pathogens. This possibility seems unlikely, considering the importance of autophagy for all processes related to plant health, including immunity. Another possibility could be the sensitivity of the autophagy pathway to heat stress. In this case, genotypes with lower autophagy and high yield could exploit other mechanisms for ameliorating stress-induced damage. As the selection of landraces was likely based on yield and end-user qualities, the tolerance traits were selected randomly. It is also plausible that our control growth conditions caused stress to some genotypes and caused activation of autophagy before we applied heat and drought stress. Testing these predictions will require developing varieties with efficient autophagy using phenotyping tools developed through our work and then examining the outcome interactions between their genotype and environment.

## Figures and Tables

**Table 1:** List of spring wheat genotypes used in this study.

LDRC	Accession	Name	Species	Type	Country Origin	City Origin
LDRC2	CItr 4175	Saracen	*Triticum aestivum* subsp. *aestivum*	Landrace	Philippines	
LDRC5	CItr 11223	Croatia 1	*Triticum aestivum* subsp. *aestivum*	Landrace	Croatia	
LDRC9	CItr 15134	Local White	*Triticum aestivum* subsp. *aestivum*	Landrace	Pakistan	
LDRC10	CItr 15144	Hallany	*Triticum aestivum* subsp. *aestivum*	Landrace	Saudi Arabia	
LDRC15	PI 8813	Kurd	*Triticum aestivum* subsp. *aestivum*	Landrace	Iraq	
LDRC16	PI 9791	Yantagbay	*Triticum aestivum* subsp. *aestivum*	Landrace	Uzbekistan	Tashkent
LDRC19	PI 43355	Pelon	*Triticum aestivum* subsp. *aestivum*	Landrace	Uruguay	
LDRC33	PI 166333	Mahlut	*Triticum aestivum* subsp. *aestivum*	Landrace	Turkey	Urfa
LDRC37	PI 185715	Ruivo	*Triticum aestivum* subsp. *aestivum*	Landrace	Portugal	Portalegre
LDRC42	PI 192147	Matte Lungo	*Triticum aestivum* subsp. *aestivum*	Landrace	Ethiopia	
LDRC43	PI 192569	Forma Vinda de Varmland	*Triticum aestivum* subsp. *aestivum*	Variety	Mozambique	
LDRC48	PI 220431	Mokhtar	*Triticum aestivum* subsp. *aestivum*	Landrace	Egypt	
LDRC65	PI 283147	Dorziyeh Karak	*Triticum aestivum* subsp. *aestivum*	Landrace	Jordan	
LDRC74	PI 366716	805	*Triticum aestivum* subsp. *aestivum*	Landrace	Afghanistan	Ghowr
LDRC81	PI 382150	Inayama	*Triticum aestivum* subsp. *aestivum*	Landrace	Japan	Kyoto
Berkut			*Triticum aestivum* subsp. *aestivum*	Variety	Mexico	

## Data Availability

The data presented in this study were derived from the RNA-seq datasets available at the following public domain: http://www.ncbi.nlm.nih.gov/geo/ (accessed on 3 July 2024). The raw data supporting the conclusions of this article will be made available by the authors on request.

## Supplementary Materials

The following supporting information can be downloaded at https://www.mdpi.com/article/10.3390/cells13141226/s1, Supplemental Figure S1: Characterization of anti-ATG8. Supplemental Figure S2: Alignment of ATG8 sequences. Conserved regions are highlighted in blue. Supplemental Figure S3: Phylogenetic analysis of ATG8. Supplemental Figure S4: Analysis of yield in the field trials. Supplemental Figure S5: Impact of heat and drought stress on ATG8 isoforms. Supplemental Figure S6: Principal component analysis biplots of the RNA-seq datasets. Supplemental Figure S7: The impact of heat and drought stress on ATG7. Supplemental Figure S8: Characterization of anti-NBR1. Supplemental Figure S9: Characterization of anti-NBR1. Supplemental Table S1: Sequences of primers used in this work. Supplemental Table S2: Features of datasets used in the study. GEO accession, references, replicate numbers, drought time, sequencer type, and read length were provided in the table. Multiple values provided rows represent respective GEO accession datasets in the same order. GEO datasets with GSE65022, GSE137780, and GSE151277 accession numbers have no publication. Supplemental Table S3: The selected RNA-seq datasets used for the analysis. RNAs were extracted from stressed and non-stressed leaves. Each genotype in the same experiment was considered as a separate dataset for differential gene expression analysis. The RNA-seq datasets were subjected to quality control. Low-quality reads (1–20%) were filtered out using the Trimmomatic application. Then, the reads were aligned to the respective reference genome. The alignment rate of the reads was around 80–95%. The number of reads was estimated using the reads mapped to each predicted gene model. Supplemental Table S4: The number of up- and downregulated differentially expressed genes (DEGs) for each analysis, along with the number of predicted and active genes. Supplemental Table S5: Summary of all ATG genes upregulated by drought stress in each RNA-seq dataset. Supplemental Table S6: Summary of ATG genes classified by flowering type based on the RNA-seq datasets. The column—up more than 50%—represents the genes that upregulated at least 50% of all datasets. Supplemental Dataset S1: Alignment of *ATG8* nucleic acid sequences used for primer design and the sequences. Supplemental Dataset S2: Alignment of ATG8 protein sequences and the sequences used for construction of the phylodendrograms. Supplemental Dataset S3: Identified ATG genes for each species used in the study. Supplemental Dataset S4: DESeq2 result table for each species used in the study. Supplemental Dataset S5: Heatmaps of all differentially expressed autophagy genes.
