# Supplementary material for: Genotype-Specific Activation of Autophagy during Heat Wave in Wheat
Source: Cells. 2024 Jul 20;13(14):1226. doi: 10.3390/cells13141226 (PMC11274669; doi:10.3390/cells13141226)
Supplement: Supplementary file 1 [file cells-13-01226-s001.zip › Supplemental Dataset 1.pdf]

Supplemental Dataset 1

Genotype-specific activation of autophagy during heat wave in wheat.

Kathleen Hickey[1], Yunus Sahin[1,2], Glenn Turner[1], Taras Nazarov[1], Vadim Jitkov[3], Mike Pumphrey[3], Andrei Smertenko[1]*
[1]Institute of Biological Chemistry, Washington State University, Pullman, WA, USA
[2]Department of Crop and Soil Sciences, University of Georgia, Athens, GA, 30602
[3]Department of Crop and Soil Sciences, Washington State University, Pullman, WA, USA

Supplemental Dataset 1. Alignment of *ATG8* nucleic acid sequences used for design of RT-qPCR primers and original sequences.

```
TaATG8_6A        ------------------------------------------------------------   0
TaATG8m_6DL      CCGGCCGGCGGCTAATAAAGTTCCTGAATTCGATGCAAAAAAAAAAATTATATATCTCCA   60
TaATG8j_5BL      ------------------------------------------------------------   0
TaATG8i_5AL      ------------------------------------------------------------   0
TaATG8k_5DL      ------------------------------------------------------------   0
TaATG8l_6BS      -CTGCCACCGGG--TTACCTTTGCCGGCTTCGCTTC------------------------   33
TaATG8f_2DS      ------------------------------------------------------------   0
TaATG8c_2AS      ------------------------------------------------------------   0
TaATG8d_2BS      ------------------------------------------------------------   0
TaATG8b_2AL      ------------------------------------------------------------   0
TaATG8e_2DL      ------------------------------------------------------------   0


TaATG8_6A        ------------------------------------------------------------   0
TaATG8m_6DL      GCACCGCCCGGCAAGATACAACCCATCTTTTAGGGCGTGCTCTGTTTCCCTTCATTCATT   120
TaATG8j_5BL      ------------------------------------------------------------   0
TaATG8i_5AL      ------------------------------------------------------------   0
TaATG8k_5DL      ------------------------------------------------------------   0
TaATG8l_6BS      -----------------------------------------------------AAAGCAT   40
TaATG8f_2DS      ------------------------------------------------------------   0
TaATG8c_2AS      -----------------------------------------------------GGAGCAT   7
TaATG8d_2BS      ------------------------------------------------------------   0
TaATG8b_2AL      ------------------------------------------------------------   0
TaATG8e_2DL      ------------------------------------------------------GAAACA   6


TaATG8_6A        ------------------------------------------------------------   0
TaATG8m_6DL      ACCTCTGCGCCGCTCAACCTAT-------------CGCCCCCAAGACCAGGCGCTGTGAG   167
TaATG8j_5BL      ------------------------------------------------------------   0
TaATG8i_5AL      ------------------------------------------------------------   0
TaATG8k_5DL      ------------------------------------------------------------   0
TaATG8l_6BS      GACTCCGCGCCGAGTCCTCTCTCCAACACCTGAGTCGCCTCCGCCTCCGTGGAGCCGCAG   100
TaATG8f_2DS      ------------------------------------------------------------   0
TaATG8c_2AS      G----CGTGA--------------------C----GGAATTCAATACAAGTTCGGTTTAT   39
TaATG8d_2BS      ------------------------------------------------------------   0
TaATG8b_2AL      ------------------------------------------------------------   0
TaATG8e_2DL      A----AGAGA--------------------TCGATGGCCACCGGCGCACGGTAGTTGGTT   42


TaATG8_6A        ------------------------------------------------------------   0
TaATG8m_6DL      CCTTCCCGTGCATCCACCCAGGGAAGAACATGGTCATCATCGTCTCTGGATCCAGGAGGC   227
TaATG8j_5BL      ------------------------------------------------------------   0
TaATG8i_5AL      ------------------------------------------------------------   0
TaATG8k_5DL      ------------------------------------------------------------   0
TaATG8l_6BS      TCCTGGTGTTGAACCGCCGCCAGATCAAGT---------------------CGACCTGGC   139
TaATG8f_2DS      ------------------------------------------------------------   0
TaATG8c_2AS      AACTCCTTTGCAC-----------AGAACG---------------------AACCGGCGA   67
```

```
TaATG8d_2BS      ------------------------------------------------------------   0
TaATG8b_2AL      ------------------------------------------------------------   0
TaATG8e_2DL      TCCG---CTTCCG-----------TGGGGG---------------------AAACGGAGG   67

TaATG8_6A        ------------------------------------------------------------   0
TaATG8m_6DL      CTGCCTGTGTCCTCCGGCGGCGCACCGGTTTTCACCCGGGAGCAGCCAGCGAGATGCATG   287
TaATG8j_5BL      ------------------------------------------------------------   0
TaATG8i_5AL      --------------------------------------GTATCGTG-----TCATCTTCT   17
TaATG8k_5DL      ------------------------------------------------------------   0
TaATG8l_6BS      CTTGCTGCAGCCCCTACGGTCGTCCGAGTTATAAT----TGCCAAG-----TGCCCGCCT   190
TaATG8f_2DS      ------------------------------------------------------------   0
TaATG8c_2AS      AACCGTGTGGCCTATAGAGTTGTGCACGCAATACAACTAGACCCCA-----AGGGGTACG   122
TaATG8d_2BS      ------GTGGCCTATAGAGTTGTGCACGCAATACAACTAGACCCCA-----AGGGGTACG   49
TaATG8b_2AL      ------------------------------------------------------------   0
TaATG8e_2DL      CGTACTCTTCCATTTT--------------ATAA----AAATCAAG-----TTATCTATT   104

TaATG8_6A        ------------------------------------------------------------   0
TaATG8m_6DL      GCGACACAGGGTATGTGGAGGCGGTGCAAGGGTTTTTTCCTTTGCACGTACGGGAGCGCC   347
TaATG8j_5BL      ------------------------------------------------------------   0
TaATG8i_5AL      TCTTC--CTCTTTCCGAGGGGTAGGGA--AGGG---------------------------   46
TaATG8k_5DL      ------------------------------------------------------------   0
TaATG8l_6BS      CGGTT--TGAGTCACCACTTGGTCAGCAGAGGCCTGACCCGCCGAGCCT-----TTCTGC   243
TaATG8f_2DS      ------------CCCCACAGGAAAAGT--CCTCTTAAATAGCCGCGACA-----CGCCAC   41
TaATG8c_2AS      GCATA--AGATTCCCCACAGGAAAGGT--CCTCTTAAATAGCCGCGACA-----CGCCAC   173
TaATG8d_2BS      GCATA--AGATTCCCCACAGGAAAGGT--CCTCTTAAATAGCCGCGACA-----CGCCAC   100
TaATG8b_2AL      ------------------------------------------------A-----ACCCCC   7
TaATG8e_2DL      TCGTT--ACGTTTCCTGCGGGTCTCGC--CTCCTCCCTTTACTTAACCA-----ACCCCC   155

TaATG8_6A        --------------ATGGTTCGTCCCTTGGAAGTGCGGTTCGTTCCCTTGTCCGGCTTTG   46
TaATG8m_6DL      GGATC-TGCTATACATGGTGAGGCCGTTTGAGGATGACTACCTGTGTCCTGCTTGGTTTG   406
TaATG8j_5BL      ------------------------------------------------------------   0
TaATG8i_5AL      -----------------------ATAAC-T-----------------TCTCCAGCTTCGC   65
TaATG8k_5DL      ------------------------------------------------------CTTCGC   6
TaATG8l_6BS      TTTTGCGCCGATTCAGCGCTGGTACAAG-CCAGCCCTGTTACAAACTTCCACCGCTGCAG   302
TaATG8f_2DS      CCACC-ACCAAACCCTACCCCG-CGACT-TCCCTCCGGTTGCTTCCGCCGAGCGACTTCC   98
TaATG8c_2AS      CCACC-ACCAAACCCTACCCCG-CGACT-TCCCTCCGGTTGCTTCCGCCGAGCGACTTCC   230
TaATG8d_2BS      CCACC-ACCAAACCCTACCCCG-CGACT-TCCCTCCGGTTGCTTCCGCCGAGCGACTTCC   157
TaATG8b_2AL      AATCC-CCCACTCTTTCCCCAA-CCCAC-GCCCTCCCGTCCCGTCTCCCCGCCGCCTCCT   64
TaATG8e_2DL      AATCC-CCCACTCTTTCCCCAA-CCCAC-GCCCTCCCGTCCCGTCTCCCCGCCGCCTCCT   212

TaATG8_6A        CACCTGGAG-----GGGATCCACCACAAGGTCCTA--------GA-GCAAAGAA---AGT   89
TaATG8m_6DL      CACCTGGAG-----GGGTCAGGGCAACACATTCCAAGGATGGATCGACCAAAAG---GTC   458
TaATG8j_5BL      --------TTGACAACAATCTATCCGCGATT------TTCAAATAAGAAACAGCAGCAGC   46
TaATG8i_5AL      CA------TTGACAACAATCTATCCGCGATTCAATCAAATACGTAGGAAACAGCAGCAGC   119
TaATG8k_5DL      CA------TTGACAACAATCTATCCGCGATTCAATCAAATACGTAGGAAACAGCAGCAGC   60
TaATG8l_6BS      CTT-CGGGCCGGCCATACCTCTGTTGCCGCCCGCGTGACTCAAGGCGCCTCTACTTCCAA   361
TaATG8f_2DS      AAT--CCAG---------TCTCCCCTCGATTCCGCCGGCCCGATCGCCTCGATCAATCTC   147
TaATG8c_2AS      AAT--CCAG---------TCTCCCCTCGATTCCGCCGGCCCGATCGCCTCGATCAATCTC   279
TaATG8d_2BS      AAT--CCAG---------TCTCCCCTCGATTCCGCCGGCCCGATCGCCTCGATCAATCTC   206
TaATG8b_2AL      CCTCCTCCGCGTCGCAAACCCTCGCCCAAATCCGCCGAATCC-----CGCGACCCAGGTG   119
TaATG8e_2DL      CCTCCTCCGCGTCGCAAACCCTCGCCCAAATCCGCCGAATCC-----CGCGACCCAGGTG   267

TaATG8_6A        A----TCCAAGGCTG------CACCTGTGTTT--GAAGTCCCTGGGTTATGGACAC-CCG   136
TaATG8m_6DL      A----TCCAAGGATAGATCGTGCTCTCCTTTT--TATATTCCAGGCCTCCGCACAG-CCG   511
TaATG8j_5BL      AACACCAGCAATCGACTCCGCGAGAACCAACAAGGGGGAGGATGAAATCCTTCAAGAAGG   106
TaATG8i_5AL      AAGACCAGCAATCGGCTCCTCGAGAACCAGCGAGGAGGAGGATGAAGTCCTTCAAGAAGG   179
TaATG8k_5DL      AAGACCAGCAATCGGCTCCTCGAGAACCAGCGAGGAGGAGGATGAAGTCCTTCAAGAAGG   120
TaATG8l_6BS      G----CCCCCGGCTGCGCCCTTCCGTCGGTTGGAGATGGCCAAGACTTGCTTCAAGACCG   417
```

```
TaATG8f_2DS      G----TCCCCGGCTGCGCCCTCCCGTCGGTTGGAGATGGCCAAGACTTGCTTCAAGACCG   203
TaATG8c_2AS      G----TCCCAGGCTGCGCCCTTCCGTCGGTTGGAGATGGCCAAGACTTGCTTCAAGACCG   335
TaATG8d_2BS      G----TCCCAGGCTGCGCCCTTCCGTCGGTTGGAGATGGCCAAGACTTGCTTCAAGACCG   262
TaATG8b_2AL      A----GCATCCGCCCTGGTTCCCGATCGATTC-CGCGTCGGTTTCCGTCGAATCAGGGAT   174
TaATG8e_2DL      A----GCATCCGCCCTGGTTCCCGATCGATTC-CGCGTCGGTTTCCGTCGAATCAGGGAT   322
                                                                *      *

TaATG8_6A        ATGTACCTCTAGAGAATGCCTATCGTGACTGTATTAACATGACTGTCGTATACCTT----   192
TaATG8m_6DL      ATGTTGCTGTTGTGGAAGAGTATCTCCGTTCTGTTAACATCATGCCTGTG----------   561
TaATG8j_5BL      AATTCACCCTGGAGGAGAGGGCGAATGAGTCGGCCGCCATGATCGCCAAGTACCCCGGCA   166
TaATG8i_5AL      AATTCACCCTGGAGGAGAGGGCGAATGAGTCGGCCGCCATGATCGCCAAGTACCCCGGCA   239
TaATG8k_5DL      AATTCACCCTGGAGGAGAGGGCGAATGAGTCGGCCGCCATGATCGCCAAGTACCCCGGCA   180
TaATG8l_6BS      AGCACCCCCTGGAAAGGAGGCAAGCTGAATCTGCTAGGATCCGTGAGAAGTACGCTGACA   477
TaATG8f_2DS      AGCACCCCCTGGAAAGGAGGCAAGCTGAATCTGCTAGGATCCGTGAGAAGTACGCTGACA   263
TaATG8c_2AS      AGCACCCCCTGGAAAGGAGGCAAGCTGAATCTGCTAGGATCCGTGAGAAGTATGCTGACA   395
TaATG8d_2BS      AGCACCCCCTGGAAAGGAGGCAAGCTGAATCTGCTAGGATCCGTGAGAAGTATGCTGACA   322
TaATG8b_2AL      TCGACCCGATCGAGAGGAGGCAGGCCGAGGCTAACCGCATAAGGGAGAAGTACTCTGACA   234
TaATG8e_2DL      TCGACCCGATCGAGAGGAGGCAGGCCGAGGCTAACCGCATAAGGGAGAAGTACTCTGACA   382
                       *  * *                          **

TaATG8_6A        -----GATTTGCACTTGCCAAGGAAAATGAAGCCTAAGCTTTCTCTGAGAGAGA---CCT   244
TaATG8m_6DL      -----TTTTTAGACATCAAGATGCCAAGGAAGTCTGTGTTTACTCTGCGTGAAA---CCT   613
TaATG8j_5BL      GGATCCCCGTGATTGTTGAAAGGTTTTCGAGGAGTAACCTTCCAGAAATGGAAAAGAGGA   226
TaATG8i_5AL      GGATCCCCGTGATTGTTGAAAGGTTTTCGAGGAGTAAACTTCCAGAAATGGAGAAGAGGA   299
TaATG8k_5DL      GGATCCCCGTGATTGTTGAAAGGTTTTCGAGGAGTAAACTTCCAGAAATGGAGAAGAGGA   240
TaATG8l_6BS      GAATTCCGGTGATCGTTGAGAAGGCTGATAAGTCTGATCTCCCGAAAATTGATAAGAGGT   537
TaATG8f_2DS      GAATTCCGGTGATCGTTGAGAAGGCTGATAAGTCTGATGTCCCGGAAATTGATAAGAAGA   323
TaATG8c_2AS      GAATTCCGGTGATCGTTGAGAAGGCTGATAAGTCTGATGTCCCGGAAATTGATAAGAAGA   455
TaATG8d_2BS      GAATTCCGGTGATCGTTGAGAAGGCTGATAAGTCTGATGTCCCGGAAATTGATAAGAAGA   382
TaATG8b_2AL      GAATTCCTGTGATCGTTGAGAAGGCTGGGAAGAGTGATATTCCTGACATTGACAAGAAAA   294
TaATG8e_2DL      GAATTCCTGTGATCGTTGAGAAGGCTGGGAAGAGTGATATTCCTGACATTGACAAGAAAA   442
                          *     *    * *      * *  *    *  *       ** *

TaATG8_6A        TCTATATGATATATGGTGGCAATACAATCAAAGAACTTGCGGAGCTTGTATGCGGTCGTC   304
TaATG8m_6DL      TTTACGTTCTTTATGGGGGAACACCTGTGAAGGAACTCTCTGAGCTCGTATGTTATCGCT   673
TaATG8j_5BL      AGTACCTGGTTCCATGTGACATGCTAGTTGGGCAGTTCATTTTCATCCTGCGCTCCAGGT   286
TaATG8i_5AL      AGTACCTGGTTCCGTGTGACATGCCAGTTGGGCAGTTCATTTTCATCCTGCGCTCCAGGT   359
TaATG8k_5DL      AGTACCTGGTTCCGTGTGACATGCCAGTTGGGCAGTTCATTTTCATCCTGCGCTCCAGGT   300
TaATG8l_6BS      ACCTTGTCCCTAACGAGATGCTAAACCCGCCAGTCCGCGACCCATCTCGCCTCAACGAGC   597
TaATG8f_2DS      AGTATCTTGTCCCGGCCGACCTCACTGTTGGCCAGTTTGTCTACGTGGTGCGGAAGAGGA   383
TaATG8c_2AS      AGTACCTTGTCCCTGCCGACCTCACTGTTGGCCAGTTTGTCTACGTGGTGCGGAAGAGGA   515
TaATG8d_2BS      AGTACCTTGTCCCTGCCGACCTCACTGTTGGCCAGTTTGTCTACGTGGTGCGGAAGAGGA   442
TaATG8b_2AL      AGTACCTTGTCCCTGCCGACCTTACAGTTGGACAGTTCGTGTACGTTGTCCGGAAGCGGA   354
TaATG8e_2DL      AGTACCTTGTCCCTGCCGACCTTACAGTTGGACAGTTCGTGTACGTTGTCCGGAAGCGGA   502
                       *

TaATG8_6A        TGAATCTTGAGGTGGGGCATCCAATGAAGTTCACCATTGATGGGACTCTTCTGCCGGAAG   364
TaATG8m_6DL      TAAATCTCGAGGAAGGTCGCCCCATGCAGTTCCTCATGAATGGTGATCTACTCGCTGAGG   733
TaATG8j_5BL      TACATCTGT----------------------CTCCAGGAACGGCGCTTTT----------   314
TaATG8i_5AL      TACATCTGT----------------------CTCCAGGGACGGCGCTTTT----------   387
TaATG8k_5DL      TACATCTGT----------------------CTCCAGGGACGGCGCTTTT----------   328
TaATG8l_6BS      GTAGTTCTA-------------------CTATGGCTGGCGCACCGTTCTG----------   628
TaATG8f_2DS      TCAAGCTGA----------------------GCCCAGAAAAGGCCATCTT----------   411
TaATG8c_2AS      TCAAGCTGA----------------------GCCCAGAAAAGGCCATCTT----------   543
TaATG8d_2BS      TCAAGCTGA----------------------GCCCAGAAAAGGCCATCTT----------   470
TaATG8b_2AL      TCAAGCTCA----------------------GTGCTGAGAAGGCGATCTT----------   382
TaATG8e_2DL      TCAAGCTCA----------------------GTGCTGAGAAGGCGATCTT----------   530
                                                   *           * *

TaATG8_6A        ATTGTGTACTCTCGTCAGTTTACAACAAGTATATTTGGGACGGGAGCATTCTGACACTCT   424
TaATG8m_6DL      CGACCACTGTGTCTGCAATCTATGAGAAACATCTTTTGTCTGATAACCTTGCTACCTTGC   793
TaATG8j_5BL      ----------------CGTGTTTGTGAAAAACACCTTGCCCCAAA-C-------AGGTAA   350
TaATG8i_5AL      ----------------CGTGTTTGTGCGCAACACCTTGCCCCAGA-C-------CGCTAA   423
```

```
TaATG8k_5DL      ----------------CGTGTTTGTGCGCAACACCTTGCCCCAGA-C-------CGCTAA   364
TaATG8l_6BS      ----------------CTGTTGCGGCCTCAAGCCGGCGCCCCCGA-GCTTCCTCCTCTGC   671
TaATG8f_2DS      ----------------CGTCTTTGTGAATAGCACCTTGCCACCGA-C-------TGCTTC   447
TaATG8c_2AS      ----------------CGTCTTTGTGAATAGCACCTTGCCACCGA-C-------TGCTTC   579
TaATG8d_2BS      ----------------CGTCTTTGTGAATAGCACCTTGCCACCGA-C-------TGCTTC   506
TaATG8b_2AL      ----------------CATCTTTGTGAAGAACACTCTTCCACCGA-C-------AGCTGC   418
TaATG8e_2DL      ----------------CATCTTTGTGAAGAACACTCTTCCACCGA-C-------AGCTGC   566
                                     *                       *            *

TaATG8_6A        GCTGCTGTGTT---------GATAATGTGAAGAAAATCTTCAACGTGGACCTGTCAGTGA   475
TaATG8m_6DL      AGTGTTACATTGACAAGAAGAAGTTTGTTCCAAGAAGCTTCAATGTCGACAAGTCAGTTA   853
TaATG8j_5BL      CCTGATGGGTAGCGTGTATGATT---CGTACAAAGAT----AAGCAGGATGGCT----TC   399
TaATG8i_5AL      CCTGATGGGTAGCGTGTATGATT---CGTACAAAGAT----AAGCAGGATGGCT----TC   472
TaATG8k_5DL      CCTGATGGGTAGCGTGTATGATT---CGTACAAAGAT----AAGCAGGATGGCT----TC   413
TaATG8l_6BS      CATGGCTCTTCGCGGCTGCCACA---CAAACACGGCATGTCACCGCGGCCTACT----TC   724
TaATG8f_2DS      GTTGATGTCAGCCATCTATGAAG---AAAACAAGGAC-------GAGGACGGCT----TC   493
TaATG8c_2AS      GTTGATGTCAGCGATCTATGAAG---AAAACAAGGAC-------GAGGACGGCT----TC   625
TaATG8d_2BS      GTTGATGTCAGCGATCTATGAAG---AAAACAAGGAC-------GAGGACGGCT----TC   552
TaATG8b_2AL      CCTGATGTCTGCCATTTACGAGG---AGAACAAGGAC-------GAGGATGGCT----TC   464
TaATG8e_2DL      CCTGATGTCTGCCATTTACGAGG---AGAACAAGGAC-------GAGGATGGCT----TC   612
                   **                                           *     *

TaATG8_6A        AAGTTCCTGTGATACCAC---CAGTGCATGTGATTGAAGATGAAGTCCCATTACCCACTC   532
TaATG8m_6DL      AAGTTCCCATCGTTGAGC---CAGTTTATGTCAATGAAGAGTACATCCCGTTTCCTCCCT   910
TaATG8j_5BL      CTCTACATGTGTTACAGCAACGAGAAGACATTTGGGTGACATCTGCCTGA---------C   450
TaATG8i_5AL      CTCTACATGTGTTACAGCAGCGAGAAGACATTTGGGTGACATCTGCCTGA---------C   523
TaATG8k_5DL      CTCTACATGTGTTACAGCAGCGAGAAGACATTTGGGTGACATCTGCCTGA---------C   464
TaATG8l_6BS      AACTCGCCCGCTTTCTCCCGTGCGCCCGTGTCACTGCCTCTGCTATCCCA---------T   775
TaATG8f_2DS      CTGTACATGACTTACAGTGGCGAGAA-----CACTTTCGGCTCTGCCTAA---------T   539
TaATG8c_2AS      CTGTACATGACTTACAGTGGCGAGAA-----CACTTTCGGCTCTGCCTAA---------T   671
TaATG8d_2BS      CTGTACATGACTTACAGTGGCGAGAA-----CACTTTCGGCTCTGCCTAA---------T   598
TaATG8b_2AL      CTCTACATGACCTACAGCGGTGAGAA-----CACCTTCGGATTGCTCTAG---------A   510
TaATG8e_2DL      CTCTACATGACCTACAGCGGCCTTTC-----GTCGCCGTTTAGGTTCTGT---------T   658
                    *        *                                 *

TaATG8_6A        TGAGGATCTGCTCTGTCCGGGACGCAAGGAGGGCGTCTATGATGAGC--AAGGTTGGAAC   590
TaATG8m_6DL      TCCGCATCCGCAACATCAGGGATGCAAAGAAGGCAGCGGCTGATGCC--AGGAGTGGCTC   968
TaATG8j_5BL      TGCAATTCAGCACACCTAAAATACT-AAGTGTAGTGCACCCAATCCC--AGTGTACATAT   507
TaATG8i_5AL      TGCAATTCAG-------------------CGTAGTGCACCCAATCCC--AGTGTACATAT   562
TaATG8k_5DL      TGCAATTCAG-------------------CGTAGTGCACCCAATCCC--AGTGTACATAT   503
TaATG8l_6BS      GGCTGCGCACTGCCCCGAGCCTCGC----CATGGCCGTGCGTCTCCTCCACTGGCAGGCC   831
TaATG8f_2DS      CCATGTGCGCTGCCACTGTAAATAA-----ATGGATGTTCAGGTTGCGACGTCTGTGTAC   594
TaATG8c_2AS      CCATGTGCGCTGCCACTGTAAATAA-ATGAATGGATGTTCAGGTTGCGACGTCTGTGTAC   730
TaATG8d_2BS      CCATGTGCGCTGCCACTGTAAATAA-ATGAATGGATGTTCAGGTTGCGACGTCTGTGTAC   657
TaATG8b_2AL      TGGCGCTGGCTGCCACGGCAATCGCTCTGAATGCCTCCTGTAAATAAGGCGTGCCAATCA   570
TaATG8e_2DL      GACTGGTGGTGTTGCAATGCAGCGC---GAGAGGGTGTCAGGACGCCGCAGTGGTGATCC   715

TaATG8_6A        CTACTCGCTGAAGGATGGTGCTCCAGCTGTGCTGGTGCACGGCACCTATGGGGAGGGCGG   650
TaATG8m_6DL      CTACGGCAATGCC--------ACCATCTCTCA-------TGGATCGTACAGCGAGGAGGG   1013
TaATG8j_5BL      GTGA---CATCAG--------T------CTCTTGTTAAATGTTAAGTTGATCAGTAGCAG   550
TaATG8i_5AL      GTGA---CATCAG--------T------CTCTTGTTAAATGTTAAGTTGATCAGTAGCAG   605
TaATG8k_5DL      GTGA---CATCAG--------T------CTCTTGTTAAATGTTAAGTTGATCAGTAGCAG   546
TaATG8l_6BS      CGCC----ACCAA--------GCACCTCCTCTGCATGGCTCGTGCGCCTCTGTCTCGGCC   879
TaATG8f_2DS      ATACTATCACTAG--------T------ATGCTGGTGGA---TTGCTCTCATGGTTTAAT   637
TaATG8c_2AS      ATACTATCACTAG--------T------ATGCTGGTGGA---TTGCTCTCATGGTTTAAT   773
TaATG8d_2BS      ATACTATCACTAG--------T------ATGCTGGTGGA---TTGCTCTCATGGTTTAAT   700
TaATG8b_2AL      ACTGTGTATATAT--------TCCGGTTTGCCTCGTCGT---AGGATCTTTAAATTGCAC   619
TaATG8e_2DL      TTGTTCTTCTCAG--------CACCATTTTTCTAGCCTATGGGCAAGAAGTGAATTGCAT   767

TaATG8_6A        GTC--A-CTAGTTTTCTATTAG--------------------------------------   669
TaATG8m_6DL      TCA--T-CTGCAGTTCTATTAGGCGACTTGGCTTAAGGGTTGGTATGAACTATGATCGGC   1070
```

```
TaATG8j_5BL      CCT--TTGCC--GATCTCTAGTTTATTTTACTTGCACGTCTCACATCTGTATTGCTCCCG   606
TaATG8i_5AL      CCT--CTGCTACAATCTCTAGTTTATTTTACTTGCACGTTTCATATCTGTATTGCTCCCG   663
TaATG8k_5DL      CCT--CTGCTACAATCTCTAGTTTATTTTACTTGCACGTTTCATATCTGTATTGCTCCCG   604
TaATG8l_6BS      ACCGGCCGCAGCCGTGCCTCGCCTACCGCGCTTC------TCCGC--GGCAT--------   923
TaATG8f_2DS      GCT--TTTAAGTGTGGTATTTTATCATCTGAATG------TTAGAACGGCCT--------   681
TaATG8c_2AS      GCT--T-TAAGTGTGGTATTTTATCATCTGAATG------TTAGAACGGCCT--------   816
TaATG8d_2BS      GCT--T-TAAGTGTGGTATTTTATCATCTGAATG------TTAGAACGGCCT--------   743
TaATG8b_2AL      AAC--CAAGAAAAACCTATTCGTATCCTGACTTA------TCCTATTTGAAT--------   663
TaATG8e_2DL      GTA--CTCCATATGGTCCTGGGTACCTATGGTTA------TCCAA------A--------   805
                                   *

TaATG8_6A        ------------------------------------------------------------   669
TaATG8m_6DL      GAATTT--TGTCAGATTGGCTGGAGTTTTGCACCCTTGTGGGGTAGTGTTAGGATCGACC   1128
TaATG8j_5BL      GCAAAA----AAAAAAACTGTATTGTTGTATCACCTAGGTAA--A--------CT-TATT   651
TaATG8i_5AL      GCAAAAGAAAAAAAAACCTGTATTGTTGTATCACCGAAGTAA--A--------CT-TATT   712
TaATG8k_5DL      GCAAAAGAAAAAAAAACCTGTATTGTTGTATCACCGAAGTAA--A--------CT-TATT   653
TaATG8l_6BS      ----CG---GCTC-----------------CCCGGTCGTCCAGCG--------CCCGCCT   951
TaATG8f_2DS      ----GT---AAAACTCTATATT---------ACGGTCGAAGATTC--------ATCAATT   717
TaATG8c_2AS      ----GT---AAAACTCTATCTATTACTATAAAGGGTCGAAGATTT--------ATCAATT   861
TaATG8d_2BS      ----GT---AAAACTCTATCTATTACTATAAAGGGTCGAAGATTT--------ATC----   784
TaATG8b_2AL      ----GT---TCTGACTTATATATATATA--TCAAGTAATCGACTC--------TGAAATT   706
TaATG8e_2DL      ----GA---CAAA--------AGATTGC--AAACAGAATCGTGCA--------ATTCATT   840

TaATG8_6A        ------------------------------------------------------------   669
TaATG8m_6DL      GGTGAATTTGGTCTGTTCTTCAGACCGACCCTTGCGCGGCAGCTTTGAATTGCTGAGGCC   1188
TaATG8j_5BL      TCCCTGTGCATGATGCTGCTTGAGAAGTCCTTTGGCAAGGATGGGCGTATATATATATAT   711
TaATG8i_5AL      TCCCTGTGCATGATGCTGCTTGCGAAGTCCTTTGGCAAGGATGGGCGTATATATGAATAG   772
TaATG8k_5DL      TCCCTGTGCATGATGCTGCTTGCGAAGTCCTTTGGCAAGGATGGGCGTATATATGAATAG   713
TaATG8l_6BS      GTCGGCGGCTGACCCCTCTGGTGCTACTCTA-GGGCGCACAGCCCTC--TTCC-GACGTC   1007
TaATG8f_2DS      CTCGTCTGGTTTATGTTCCT-----CATGCT-CTGGGATGATTTCAA--TCGC------A   763
TaATG8c_2AS      CTCGTCTGGTTTATGTTCCT-----CATGCT-CTGGGATGATTTCAA--TCGC------A   907
TaATG8d_2BS      ------------------------------------------------------------   784
TaATG8b_2AL      TGGTAAAATCTACTGCTCCTGCGTTAATGCA-TGCCGCTGTGGTTAC--TTGTTCACTGC   763
TaATG8e_2DL      TTCCAAAACTTCAGTTGGCGGATTTGTTGCT-GTGCGTTGATGACCT--TTGG-CCCGAC   896

TaATG8_6A        ------------------------------------------------------------   669
TaATG8m_6DL      AGTTTGAATTATGCTAAACCTTCATGGATGATGTTATGGGCGTTCAGGCTTACTTTCAGG   1248
TaATG8j_5BL      ATATA-TATATATATATATATA----TATATATATATATATA------------------   748
TaATG8i_5AL      AAGTG-TTGATTGTGA--------------------------------------------   787
TaATG8k_5DL      AAGTG-TTGATTGTGATGAAGT----AAACGCCTCCTGCTGA------------------   750
TaATG8l_6BS      CTGCG-ATGG-AGCCACCCGTAGCTCCCTACTCCTTGCTGCTTTCC--------------   1051
TaATG8f_2DS      AATTA-GTGGCAGCAGAACTGAGTTTTTTTTTATTATATTGTTTC---------------   807
TaATG8c_2AS      AATTA-GTGGCAGCAGAACCG---------------------------------------   927
TaATG8d_2BS      ------------------------------------------------------------   784
TaATG8b_2AL      TATTG-ATGT-GATGTTTCATAGATCCGTGTAGATCTCAGCGTTGC--------------   807
TaATG8e_2DL      CAGTC-TTGTGGCTGTTTGCTA----GGCTGTGTTCACTGTTTTGC--------------   937

TaATG8_6A        ------------------------------------------------------------   669
TaATG8m_6DL      GTTGTTTTCTGATTTTTGCTGTGGTTGCTCTTCTTGCTTTCACTAACTCTGTCAAGTGCC   1308
TaATG8j_5BL      ---------TATATATGAATAAAAGTGTTGATTGTGA-----------------------   776
TaATG8i_5AL      ------------------------------------------------------------   787
TaATG8k_5DL      ---------G--------------------------------------------------   751
TaATG8l_6BS      ---------ACCAACTGCGCCGCATCGCTCGTGCTCCCCTC---------------CTCG   1087
TaATG8f_2DS      ---------------ACACACCTTGTCCTTTT----------------------------   824
TaATG8c_2AS      ------------------------------------------------------------   927
TaATG8d_2BS      ------------------------------------------------------------   784
TaATG8b_2AL      ---------CAGTAATAAATCTGGGCGCTGTT-----------------------------   830
TaATG8e_2DL      ------------------------------------------------------------   937
```

```
TaATG8_6A        ------------------------------------------------------------   669
TaATG8m_6DL      AAACACTGATTAACCTCTACTCTAAGAACATGCTC---AAGGCAC------GTGGCTTAT   1359
TaATG8j_5BL      ------------------------------------------------------------   776
TaATG8i_5AL      ------------------------------------------------------------   787
TaATG8k_5DL      ------------------------------------------------------------   751
TaATG8l_6BS      TCACGCCGGGCCGCCTCCGCCCGTAGGACGACGTCCACTCCGCGCGTTCAGGCTGCCTGT   1147
TaATG8f_2DS      ------------------------------------------------------------   824
TaATG8c_2AS      ------------------------------------------------------------   927
TaATG8d_2BS      ------------------------------------------------------------   784
TaATG8b_2AL      ------------------------------------------------------------   830
TaATG8e_2DL      ------------------------------------------------------------   937


TaATG8_6A        ----------------------------- 669
TaATG8m_6DL      TAGATTGAATAACT--GCCATCTTGTGTT 1386
TaATG8j_5BL      ----------------------------- 776
TaATG8i_5AL      ----------------------------- 787
TaATG8k_5DL      ----------------------------- 751
TaATG8l_6BS      TGGCTCCGCTCACCGTGCCTGCTTGGCT- 1175
TaATG8f_2DS      ----------------------------- 824
TaATG8c_2AS      ----------------------------- 927
TaATG8d_2BS      ----------------------------- 784
TaATG8b_2AL      ----------------------------- 830
TaATG8e_2DL      ----------------------------- 937
```

ATG8 sequences

```
>TaATG8i_5AL
GTATCGTGTCATCTTCTTCTTCCTCTTTCCGAGGGGTAGGGAAGGGATAACTTCTCCAGC
TTCGCCATTGACAACAATCTATCCGCGATTCAATCAAATACGTAGGAAACAGCAGCAGCA
AGACCAGCAATCGGCTCCTCGAGAACCAGCGAGGAGGAGGATGAAGTCCTTCAAGAAGGA
ATTCACCCTGGAGGAGAGGGCGAATGAGTCGGCCGCCATGATCGCCAAGTACCCCGGCAG
GATCCCCGTGATTGTTGAAAGGTTTTCGAGGAGTAAACTTCCAGAAATGGAGAAGAGGAA
GTACCTGGTTCCGTGTGACATGCCAGTTGGGCAGTTCATTTTCATCCTGCGCTCCAGGTT
ACATCTGTCTCCAGGGACGGCGCTTTTCGTGTTTGTGCGCAACACCTTGCCCCAGACCGC
TAACCTGATGGGTAGCGTGTATGATTCGTACAAAGATAAGCAGGATGGCTTCCTCTACAT
GTGTTACAGCAGCGAGAAGACATTTGGGTGACATCTGCCTGACTGCAATTCAGCGTAGTG
CACCCAATCCCAGTGTACATATGTGACATCAGTCTCTTGTTAAATGTTAAGTTGATCAGT
AGCAGCCTCTGCTACAATCTCTAGTTTATTTTACTTGCACGTTTCATATCTGTATTGCTC
CCGGCAAAAGAAAAAAAAACCTGTATTGTTGTATCACCGAAGTAAACTTATTTCCCTGTG
CATGATGCTGCTTGCGAAGTCCTTTGGCAAGGATGGGCGTATATATGAATAGAAGTGTTG
ATTGTGA

>TaATG8j_5BL
TTGACAACAATCTATCCGCGATTTTCAAATAAGAAACAGCAGCAGCAACACCAGCAATCG
ACTCCGCGAGAACCAACAAGGGGGAGGATGAAATCCTTCAAGAAGGAATTCACCCTGGAG
GAGAGGGCGAATGAGTCGGCCGCCATGATCGCCAAGTACCCCGGCAGGATCCCCGTGATT
GTTGAAAGGTTTTCGAGGAGTAACCTTCCAGAAATGGAAAAGAGGAAGTACCTGGTTCCA
TGTGACATGCTAGTTGGGCAGTTCATTTTCATCCTGCGCTCCAGGTTACATCTGTCTCCA
GGAACGGCGCTTTTCGTGTTTGTGAAAAACACCTTGCCCCAAACAGGTAACCTGATGGGT
AGCGTGTATGATTCGTACAAAGATAAGCAGGATGGCTTCCTCTACATGTGTTACAGCAAC
GAGAAGACATTTGGGTGACATCTGCCTGACTGCAATTCAGCACACCTAAAATACTAAGTG
TAGTGCACCCAATCCCAGTGTACATATGTGACATCAGTCTCTTGTTAAATGTTAAGTTGA
TCAGTAGCAGCCTTTGCCGATCTCTAGTTTATTTTACTTGCACGTCTCACATCTGTATTG
CTCCCGGCAAAAAAAAAAACTGTATTGTTGTATCACCTAGGTAAACTTATTTCCCTGTGC
ATGATGCTGCTTGAGAAGTCCTTTGGCAAGGATGGGCGTATATATATATATATATATATA
TATATATATATATATATATATATATATATATATATGAATAAAAGTGTTGATTGTGA

>TaATG8k_5DL
CTTCGCCATTGACAACAATCTATCCGCGATTCAATCAAATACGTAGGAAACAGCAGCAGC
AAGACCAGCAATCGGCTCCTCGAGAACCAGCGAGGAGGAGGATGAAGTCCTTCAAGAAGG
AATTCACCCTGGAGGAGAGGGCGAATGAGTCGGCCGCCATGATCGCCAAGTACCCCGGCA
GGATCCCCGTGATTGTTGAAAGGTTTTCGAGGAGTAAACTTCCAGAAATGGAGAAGAGGA
```

AGTACCTGGTTCCGTGTGACATGCCAGTTGGGCAGTTCATTTTCATCCTGCGCTCCAGGT
TACATCTGTCTCCAGGGACGGCGCTTTTCGTGTTTGTGCGCAACACCTTGCCCCAGACCG
CTAACCTGATGGGTAGCGTGTATGATTCGTACAAAGATAAGCAGGATGGCTTCCTCTACA
TGTGTTACAGCAGCGAGAAGACATTTGGGTGACATCTGCCTGACTGCAATTCAGCGTAGT
GCACCCAATCCCAGTGTACATATGTGACATCAGTCTCTTGTTAAATGTTAAGTTGATCAG
TAGCAGCCTCTGCTACAATCTCTAGTTTATTTTACTTGCACGTTTCATATCTGTATTGCT
CCCGGCAAAAGAAAAAAAAACCTGTATTGTTGTATCACCGAAGTAAACTTATTTCCCTGT
GCATGATGCTGCTTGCGAAGTCCTTTGGCAAGGATGGGCGTATATATGAATAGAAGTGTT
GATTGTGATGAAGTAAACGCCTCCTGCTGAG

>TaATG8l_6BS
CTGCCACCGGGTTACCTTTGCCGGCTTCGCTTCAAAGCATGACTCCGCGCCGAGTCCTCT
CTCCAACACCTGAGTCGCCTCCGCCTCCGTGGAGCCGCAGTCCTGGTGTTGAACCGCCGC
CAGATCAAGTCGACCTGGCCTTGCTGCAGCCCCTACGGTCGTCCGAGTTATAATTGCCAA
GTGCCCGCCTCGGTTTGAGTCACCACTTGGTCAGCAGAGGCCTGACCCGCCGAGCCTTTC
TGCTTTTGCGCCGATTCAGCGCTGGTACAAGCCAGCCCTGTTACAAACTTCCACCGCTGC
AGCTTCGGGCCGGCCATACCTCTGTTGCCGCCCGCGTGACTCAAGGCGCCTCTACTTCCA
AGCCCCCGGCTGCGCCCTTCCGTCGGTTGGAGATGGCCAAGACTTGCTTCAAGACCGAGC
ACCCCCTGGAAAGGAGGCAAGCTGAATCTGCTAGGATCCGTGAGAAGTACGCTGACAGAA
TTCCGGTGATCGTTGAGAAGGCTGATAAGTCTGATCTCCCGAAAATTGATAAGAGGTACC
TTGTCCCTAACGAGATGCTAAACCCGCCAGTCCGCGACCCATCTCGCCTCAACGAGCGTA
GTTCTACTATGGCTGGCGCACCGTTCTGCTGTTGCGGCCTCAAGCCGGCGCCCCCGAGCT
TCCTCCTCTGCCATGGCTCTTCGCGGCTGCCACACAAACACGGCATGTCACCGCGGCCTA
CTTCAACTCGCCCGCTTTCTCCCGTGCGCCCGTGTCACTGCCTCTGCTATCCCATGGCTG
CGCACTGCCCCGAGCCTCGCCATGGCCGTGCGTCTCCTCCACTGGCAGGCCCGCCACCAA
GCACCTCCTCTGCATGGCTCGTGCGCCTCTGTCTCGGCCACCGGCCGCAGCCGTGCCTCG
CCTACCGCGCTTCTCCGCGGCATCGGCTCCCCGGTCGTCCAGCGCCCGCCTGTCGGCGGC
TGACCCCTCTGGTGCTACTCTAGGGCGCACAGCCCTCTTCCGACGTCCTGCGATGGAGCC
ACCCGTAGCTCCCTACTCCTTGCTGCTTTCCACCAACTGCGCCGCATCGCTCGTGCTCCC
CTCCTCGTCACGCCGGGCCGCCTCCGCCCGTAGGACGACGTCCACTCCGCGCGTTCAGGC
TGCCTGTTGGCTCCGCTCACCGTGCCTGCTTGGCT

>TaATG8m_6DL
CCGGCCGGCGGCTAATAAAGTTCCTGAATTCGATGCAAAAAAAAAAAATTATATATCTCCA
GCACCGCCCGGCAAGATACAACCCATCTTTTAGGGCGTGCTCTGTTTCCCTTCATTCATT
ACCTCTGCGCCGCTCAACCTATCGCCCCCAAGACCAGGCGCTGTGAGCCTTCCCGTGCAT
CCACCCAGGGAAGAACATGGTCATCATCGTCTCTGGATCCAGGAGGCCTGCCTGTGTCCT
CCGGCGGCGCACCGGTTTTCACCCGGGAGCAGCCAGCGAGATGCATGGCGACACAGGGTA
TGTGGAGGCGGTGCAAGGGTTTTTTCCTTTGCACGTACGGGAGCGCCGGATCTGCTATAC
ATGGTGAGGCCGTTTGAGGATGACTACCTGTGTCCTGCTTGGTTTGCACCTGGAGGGGTC
AGGGCAACACATTCCAAGGATGGATCGACCAAAAGGTCATCCAAGGATAGATCGTGCTCT
CCTTTTTATATTCCAGGCCTCCGCACAGCCGATGTTGCTGTTGTGGAAGAGTATCTCCGT
TCTGTTAACATCATGCCTGTGTTTTTAGACATCAAGATGCCAAGGAAGTCTGTGTTTACT
CTGCGTGAAACCTTTTACGTTCTTTATGGGGGAACACCTGTGAAGGAACTCTCTGAGCTC
GTATGTTATCGCTTAAATCTCGAGGAAGGTCGCCCCATGCAGTTCCTCATGAATGGTGAT
CTACTCGCTGAGGCGACCACTGTGTCTGCAATCTATGAGAAACATCTTTTGTCTGATAAC
CTTGCTACCTTGCAGTGTTACATTGACAAGAAGAAGTTTGTTCCAAGAAGCTTCAATGTC
GACAAGTCAGTTAAAGTTCCCATCGTTGAGCCAGTTTATGTCAATGAAGAGTACATCCCG
TTTCCTCCCTTCCGCATCCGCAACATCAGGGATGCAAAGAAGGCAGCGGCTGATGCCAGG
AGTGGCTCCTACGGCAATGCCACCATCTCTCATGGATCGTACAGCGAGGAGGGTCATCTG
CAGTTCTATTAGGCGACTTGGCTTAAGGGTTGGTATGAACTATGATCGGCGAATTTTGTC
AGATTGGCTGGAGTTTTGCACCCTTGTGGGGTAGTGTTAGGATCGACCGGTGAATTTGGT
CTGTTCTTCAGACCGACCCTTGCGCGGCAGCTTTGAATTGCTGAGGCCAGTTTGAATTAT
GCTAAACCTTCATGGATGATGTTATGGGCGTTCAGGCTTACTTTCAGGGTTGTTTTCTGA
TTTTTGCTGTGGTTGCTCTTCTTGCTTTCACTAACTCTGTCAAGTGCCAAACACTGATTA
ACCTCTACTCTAAGAACATGCTCAAGGCACGTGGCTTATTAGATTGAATAACTGCCATCT
TGTGTT

>TaATG8c_2AS
GGAGCATGCGTGACGGAATTCAATACAAGTTCGGTTTATAACTCCTTTGCACAGAACGAA
CCGGCGAAACCGTGTGGCCTATAGAGTTGTGCACGCAATACAACTAGACCCCAAGGGGTA
CGGCATAAGATTCCCCACAGGAAAGGTCCTCTTAAATAGCCGCGACACGCCACCCACCAC
CAAACCCTACCCCGCGACTTCCCTCCGGTTGCTTCCGCCGAGCGACTTCCAATCCAGTCT

CCCCTCGATTCCGCCGGCCCGATCGCCTCGATCAATCTCGTCCCAGGCTGCGCCCTTCCG
TCGGTTGGAGATGGCCAAGACTTGCTTCAAGACCGAGCACCCCCTGGAAAGGAGGCAAGC
TGAATCTGCTAGGATCCGTGAGAAGTATGCTGACAGAATTCCGGTGATCGTTGAGAAGGC
TGATAAGTCTGATGTCCCGGAAATTGATAAGAAGAAGTACCTTGTCCCTGCCGACCTCAC
TGTTGGCCAGTTTGTCTACGTGGTGCGGAAGAGGATCAAGCTGAGCCCAGAAAAGGCCAT
CTTCGTCTTTGTGAATAGCACCTTGCCACCGACTGCTTCGTTGATGTCAGCGATCTATGA
AGAAAACAAGGACGAGGACGGCTTCCTGTACATGACTTACAGTGGCGAGAACACTTTCGG
CTCTGCCTAATCCATGTGCGCTGCCACTGTAAATAAATGAATGGATGTTCAGGTTGCGAC
GTCTGTGTACATACTATCACTAGTATGCTGGTGGATTGCTCTCATGGTTTAATGCTTTAA
GTGTGGTATTTTATCATCTGAATGTTAGAACGGCCTGTAAAACTCTATCTATTACTATAA
AGGGTCGAAGATTTATCAATTCTCGTCTGGTTTATGTTCCTCATGCTCTGGGATGATTTC
AATCGCAAATTAGTGGCAGCAGAACCG

>TaATG8d_2BS
GTGGCCTATAGAGTTGTGCACGCAATACAACTAGACCCCAAGGGGTACGGCATAAGATTC
CCCACAGGAAAGGTCCTCTTAAATAGCCGCGACACGCCACCCACCACCAAACCCTACCCC
GCGACTTCCCTCCGGTTGCTTCCGCCGAGCGACTTCCAATCCAGTCTCCCCTCGATTCCG
CCGGCCCGATCGCCTCGATCAATCTCGTCCCAGGCTGCGCCCTTCCGTCGGTTGGAGATG
GCCAAGACTTGCTTCAAGACCGAGCACCCCCTGGAAAGGAGGCAAGCTGAATCTGCTAGG
ATCCGTGAGAAGTATGCTGACAGAATTCCGGTGATCGTTGAGAAGGCTGATAAGTCTGAT
GTCCCGGAAATTGATAAGAAGAAGTACCTTGTCCCTGCCGACCTCACTGTTGGCCAGTTT
GTCTACGTGGTGCGGAAGAGGATCAAGCTGAGCCCAGAAAAGGCCATCTTCGTCTTTGTG
AATAGCACCTTGCCACCGACTGCTTCGTTGATGTCAGCGATCTATGAAGAAAACAAGGAC
GAGGACGGCTTCCTGTACATGACTTACAGTGGCGAGAACACTTTCGGCTCTGCCTAATCC
ATGTGCGCTGCCACTGTAAATAAATGAATGGATGTTCAGGTTGCGACGTCTGTGTACATA
CTATCACTAGTATGCTGGTGGATTGCTCTCATGGTTTAATGCTTTAAGTGTGGTATTTTA
TCATCTGAATGTTAGAACGGCCTGTAAAACTCTATCTATTACTATAAAGGGTCGAAGATT
TATC

>TaATG8f_2DS
CCCCACAGGAAAAGTCCTCTTAAATAGCCGCGACACGCCACCCACCACCAAACCCTACCC
CGCGACTTCCCTCCGGTTGCTTCCGCCGAGCGACTTCCAATCCAGTCTCCCCTCGATTCC
GCCGGCCCGATCGCCTCGATCAATCTCGTCCCCGGCTGCGCCCTCCCGTCGGTTGGAGAT
GGCCAAGACTTGCTTCAAGACCGAGCACCCCCTGGAAAGGAGGCAAGCTGAATCTGCTAG
GATCCGTGAGAAGTACGCTGACAGAATTCCGGTGATCGTTGAGAAGGCTGATAAGTCTGA
TGTCCCGGAAATTGATAAGAAGAAGTATCTTGTCCCGGCCGACCTCACTGTTGGCCAGTT
TGTCTACGTGGTGCGGAAGAGGATCAAGCTGAGCCCAGAAAAGGCCATCTTCGTCTTTGT
GAATAGCACCTTGCCACCGACTGCTTCGTTGATGTCAGCCATCTATGAAGAAAACAAGGA
CGAGGACGGCTTCCTGTACATGACTTACAGTGGCGAGAACACTTTCGGCTCTGCCTAATC
CATGTGCGCTGCCACTGTAAATAAATGGATGTTCAGGTTGCGACGTCTGTGTACATACTA
TCACTAGTATGCTGGTGGATTGCTCTCATGGTTTAATGCTTTTAAGTGTGGTATTTTATC
ATCTGAATGTTAGAACGGCCTGTAAAACTCTATATTACGGTCGAAGATTCATCAATTCTC
GTCTGGTTTATGTTCCTCATGCTCTGGGATGATTTCAATCGCAAATTAGTGGCAGCAGAA
CTGAGTTTTTTTTTATTATATTGTTTCACACACCTTGTCCTTTT

>TaATG8b_2AL
AACCCCCAATCCCCCACTCTTTCCCCAACCCACGCCCTCCCGTCCCGTCTCCCCGCCGCC
TCCTCCTCCTCCGCGTCGCAAACCCTCGCCCAAATCCGCCGAATCCCGCGACCCAGGTGA
GCATCCGCCCTGGTTCCCGATCGATTCCGCGTCGGTTTCCGTCGAATCAGGGATTCGACC
CGATCGAGAGGAGGCAGGCCGAGGCTAACCGCATAAGGGAGAAGTACTCTGACAGAATTC
CTGTGATCGTTGAGAAGGCTGGGAAGAGTGATATTCCTGACATTGACAAGAAAAAGTACC
TTGTCCCTGCCGACCTTACAGTTGGACAGTTCGTGTACGTTGTCCGGAAGCGGATCAAGC
TCAGTGCTGAGAAGGCGATCTTCATCTTTGTGAAGAACACTCTTCCACCGACAGCTGCCC
TGATGTCTGCCATTTACGAGGAGAACAAGGACGAGGATGGCTTCCTCTACATGACCTACA
GCGGTGAGAACACCTTCGGATTGCTCTAGATGGCGCTGGCTGCCACGGCAATCGCTCTGA
ATGCCTCCTGTAAATAAGGCGTGCCAATCAACTGTGTATATATTCCGGTTTGCCTCGTCG
TAGGATCTTTAAATTGCACAACCAAGAAAAACCTATTCGTATCCTGACTTATCCTATTTG
AATGTTCTGACTTATATATATATATATCAAGTAATCGACTCTGAAATTTGGTAAAATCTACT
GCTCCTGCGTTAATGCATGCCGCTGTGGTTACTTGTTCACTGCTATTGATGTGATGTTTC
ATAGATCCGTGTAGATCTCAGCGTTGCCAGTAATAAATCTGGGCGCTGTT

>TaATG8e_2DL
GAAACAAAGAGATCGATGGCCACCGGCGCACGGTAGTTGGTTTCCGCTTCCGTGGGGGAA

ACGGAGGCGTACTCTTCCATTTTATAAAAATCAAGTTATCTATTTCGTTACGTTTCCTGC
GGGTCTCGCCTCCTCCCTTTACTTAACCAACCCCCAATCCCCCACTCTTTCCCCAACCCA
CGCCCTCCCGTCCCGTCTCCCCGCCGCCTCCTCCTCCTCCGCGTCGCAAACCCTCGCCCA
AATCCGCCGAATCCCGCGACCCAGGTGAGCATCCGCCCTGGTTCCCGATCGATTCCGCGT
CGGTTTCCGTCGAATCAGGGATTCGACCCGATCGAGAGGAGGCAGGCCGAGGCTAACCGC
ATAAGGGAGAAGTACTCTGACAGAATTCCTGTGATCGTTGAGAAGGCTGGGAAGAGTGAT
ATTCCTGACATTGACAAGAAAAAGTACCTTGTCCCTGCCGACCTTACAGTTGGACAGTTC
GTGTACGTTGTCCGGAAGCGGATCAAGCTCAGTGCTGAGAAGGCGATCTTCATCTTTGTG
AAGAACACTCTTCCACCGACAGCTGCCCTGATGTCTGCCATTTACGAGGAGAACAAGGAC
GAGGATGGCTTCCTCTACATGACCTACAGCGGCCTTTCGTCGCCGTTTAGGTTCTGTTGA
CTGGTGGTGTTGCAATGCAGCGCGAGAGGGTGTCAGGACGCCGCAGTGGTGATCCTTGTT
CTTCTCAGCACCATTTTTCTAGCCTATGGGCAAGAAGTGAATTGCATGTACTCCATATGG
TCCTGGGTACCTATGGTTATCCAAAGACAAAAGATTGCAAACAGAATCGTGCAATTCATT
TTCCAAAACTTCAGTTGGCGGATTTGTTGCTGTGCGTTGATGACCTTTGGCCCGACCAGT
CTTGTGGCTGTTTGCTAGGCTGTGTTCACTGTTTTGC

>TaATG8i_5AL
GTATCGTGTCATCTTCTTCTTCCTCTTTCCGAGGGGTAGGGAAGGGATAACTTCTCCAGC
TTCGCCATTGACAACAATCTATCCGCGATTCAATCAAATACGTAGGAAACAGCAGCAGCA
AGACCAGCAATCGGCTCCTCGAGAACCAGCGAGGAGGAGGATGAAGTCCTTCAAGAAGGA
ATTCACCCTGGAGGAGAGGGCGAATGAGTCGGCCGCCATGATCGCCAAGTACCCCGGCAG
GATCCCCGTGATTGTTGAAAGGTTTTCGAGGAGTAAACTTCCAGAAATGGAGAAGAGGAA
GTACCTGGTTCCGTGTGACATGCCAGTTGGGCAGTTCATTTTCATCCTGCGCTCCAGGTT
ACATCTGTCTCCAGGGACGGCGCTTTTCGTGTTTGTGCGCAACACCTTGCCCCAGACCGC
TAACCTGATGGGTAGCGTGTATGATTCGTACAAAGATAAGCAGGATGGCTTCCTCTACAT
GTGTTACAGCAGCGAGAAGACATTTGGGTGACATCTGCCTGACTGCAATTCAGCGTAGTG
CACCCAATCCCAGTGTACATATGTGACATCAGTCTCTTGTTAAATGTTAAGTTGATCAGT
AGCAGCCTCTGCTACAATCTCTAGTTTATTTTACTTGCACGTTTCATATCTGTATTGCTC
CCGGCAAAAGAAAAAAAAACCTGTATTGTTGTATCACCGAAGTAAACTTATTTCCCTGTG
CATGATGCTGCTTGCGAAGTCCTTTGGCAAGGATGGGCGTATATATGAATAGAAGTGTTG
ATTGTGA
