# Supplementary material for: Genotype-Specific Activation of Autophagy during Heat Wave in Wheat
Source: Cells. 2024 Jul 20;13(14):1226. doi: 10.3390/cells13141226 (PMC11274669; doi:10.3390/cells13141226)
Supplement: Supplementary file 1 [file cells-13-01226-s001.zip › Supplemental Dataset 2.pdf]

Supplemental Dataset 2

Genotype-specific activation of autophagy during heat wave in wheat.

Kathleen Hickey[1], Yunus Sahin[1,2], Glenn Turner[1], Taras Nazarov[1], Vadim Jitkov[3], Mike Pumphrey[3], Andrei Smertenko[1*]
[1]Institute of Biological Chemistry, Washington State University, Pullman, WA, USA
[2]Department of Crop and Soil Sciences, University of Georgia, Athens, GA, 30602
[3]Department of Crop and Soil Sciences, Washington State University, Pullman, WA, USA

Supplemental Dataset 2. The alignment of ATG8 amino acid sequences in NEXUS format used for phylogenetic analysis and corresponding ATG8 peptide sequences.

```
#NEXUS
BEGIN DATA;
dimensions ntax=26 nchar=293;
format missing=?
interleave datatype=PROTEIN gap= -;

matrix
AtATG8h      ------------MGIVVKS--FKDQFS-----------------------
AtATG8i      ----------------MKS--FKEQYT-----------------------
TaATG8i_5AL  ----------------MKS--FKKEFT-----------------------
TaATG8k_5DL  -------------------------MD-----------------------
TaATG8j_5BL  ----------------MKS--FKKEFT-----------------------
OsATG8d      ----------------MKPRPFKEEFT-----------------------
TaATG8b_2AL  ---------------MAK-SSFKLEHP-----------------------
TaATG8b_2BL  ---------------MAK-SSFKLEHP-----------------------
TaATG8e_2DL  --------------------------P-----------------------
OsATG8b      ---------------MAK-SSFKLDHT-----------------------
OsATG8c      ---------------MAR-SSFKLEHP-----------------------
AtATG8c      ---------------MAN-SSFKLEHP-----------------------
AtATG8d      ---------------MAI-SSFKHEHPLVEMNIDVEVTKFHLSLLCSVVE
TaATG8c_2AS  ---------------MAK-TCFKTEHP-----------------------
TaATG8d_2BS  ---------------MAK-TCFKTEHP-----------------------
TaATG8f_2DS  ---------------MAK-TCFKTEHP-----------------------
OsATG8a      ---------------MAR-TSFKLEHP-----------------------
AtATG8a      MIFACLKFAETNRIAMAK-SSFKISNP-----------------------
AtATG8b      ---------------MEK-NSFKLSNP-----------------------
AtATG8e      ---------------MNKGSIFKMDND-----------------------
AtATG8f      ---------------MAK-SSFKQEHD-----------------------
AtATG8g      ---------------MSN-VSFRQDHD-----------------------
ScATG8       ---------------MKS--TFKSEYP-----------------------
TaATG8l_6BS  ----------------MAKTCFKTEHP-----------------------
TaATG8_6A    -------MVRPLEVRFVPLSGFAPGGDPPQGPR-----------------
TaATG8m_6DL  -------MVRPFEDDYLCPAWFAPGGVRATHSKD----------------

AtATG8h      ---------------------SDERLKESNNIIAKYPDRIPVIIEKYSN-
AtATG8i      ---------------------LDERLAESREIIAKYPTRIPVIAEKYCK-
TaATG8i_5AL  ---------------------LEERANESAAMIAKYPGRIPVIVERFSR-
TaATG8k_5DL  ---------------------AEERANESAAMIAKYPGRIPVIVERFSR-
```

```
TaATG8j_5BL  ---------------------LEERANESAAMIAKYPGRIPVIVERFSR-
OsATG8d      ---------------------LEERAKESAAMIASYPDRIPVIVEKFSR-
TaATG8b_2AL  ---------------------LERRQAEATRIREKYSDRIPVIVEKAGK-
TaATG8b_2BL  ---------------------LERRQAEANRIREKYSDRIPVIVEKAGK-
TaATG8e_2DL  ---------------------IERRQAEANRIREKYSDRIPVIVEKAGK-
OsATG8b      ---------------------LERRQAEANRIREKYSDRIPVIVEKAER-
OsATG8c      ---------------------LERRQAEANRIREKYPDRIPVIVEKAER-
AtATG8c      ---------------------LERRQIESSRIREKYPDRIPVIVERAER-
AtATG8d      SLLFTGRRIAISVPWNPKILSLKKRQAEAARIREKYPDRIPVIVERAEK-
TaATG8c_2AS  ---------------------LERRQAESARIREKYADRIPVIVEKADK-
TaATG8d_2BS  ---------------------LERRQAESARIREKYADRIPVIVEKADK-
TaATG8f_2DS  ---------------------LERRQAESARIREKYADRIPVIVEKADK-
OsATG8a      ---------------------LERRQAESARIREKYSDRIPVIVEKADK-
AtATG8a      ---------------------LEARMSESSRIREKYPDRIPVIVEKAGQ-
AtATG8b      ---------------------LEMRMAESTRIRAKYPERVPVIVEKAGQ-
AtATG8e      ---------------------FEKRKAEAGRIREKYPDRIPVIVEKAEK-
AtATG8f      ---------------------LEKRRAEAARIREKYPDRIPVIVEKAEK-
AtATG8g      ---------------------FEKRKAEALRIREKYSDRVPVIVEKSEK-
ScATG8       ---------------------FEKRKTESERIADRFKNRIPVICEKAEK-
TaATG8l_6BS  ---------------------LERRQAESARIREKYADRIPVIVEKADK-
TaATG8_6A    -------AKKVSK--AAPVFEVPGLWTPDVPLENAYRDCINMTVVYLDLH
TaATG8m_6DL  -----GSTKRSSKDRSCSPFYIPGLRTADVAVVEEYLRSVNIMPVFLDIK

AtATG8h      -----ADLPDMEKNKYLVPRDMTVGHFIHMLSKR---------------M
AtATG8i      -----TDLPAIEKKKFLVPRDMSVGQFIYILSAR---------------L
TaATG8i_5AL  -----SKLPEMEKRKYLVPCDMPVGQFIFILRSR---------------L
TaATG8k_5DL  -----SNLPEMEKRKYLVPCDMPVGQFIFILRSR---------------L
TaATG8j_5BL  -----SNLPEMEKRKYLVPCDMLVGQFIFILRSR---------------L
OsATG8d      -----SNLPEMEKRKYLVPCNMPVGQFIFILRSR---------------L
TaATG8b_2AL  -----SDIPDIDKKKYLVPADLTVGQFVYVVRKR---------------I
TaATG8b_2BL  -----SDIPDIDKKKYLVPADLTVGQFVYVVRKR---------------I
TaATG8e_2DL  -----SDIPDIDKKKYLVPADLTVGQFVYVVRKR---------------I
OsATG8b      -----SDIPDIDKKKYLVPADLTVGQFVYVVRKR---------------I
OsATG8c      -----SDIPDIDKKKYLVPADLTVGQFVYVVRKR---------------I
AtATG8c      -----SDVPNIDKKKYLVPADLTVGQFVYVVRKR---------------I
AtATG8d      -----SDVPDIDRKKYLVPADLTVGQFVYVVRKR---------------I
TaATG8c_2AS  -----SDVPEIDKKKYLVPADLTVGQFVYVVRKR---------------I
TaATG8d_2BS  -----SDVPEIDKKKYLVPADLTVGQFVYVVRKR---------------I
TaATG8f_2DS  -----SDVPEIDKKKYLVPADLTVGQFVYVVRKR---------------I
OsATG8a      -----TDVPEIDKKKYLVPADLTVGQFVYVVRKR---------------I
AtATG8a      -----SDVPDIDKKKYLVPADLTVGQFVYVVRKR---------------I
AtATG8b      -----SDVPDIDKKKYLVPADLTIGQFVYVVRKR---------------I
AtATG8e      -----SEVPNIDKKKYLVPSDLTVGQFVYVIRKR---------------I
AtATG8f      -----SDIPTIDKKKYLVPADLTVGQFVYVIRKR---------------I
AtATG8g      -----SDIPNIDKKKYLVPADLTVGQFVYVIRKR---------------I
ScATG8       -----SDIPEIDKRKYLVPADLTVGQFVYVIRKR---------------I
TaATG8l_6BS  -----SDLPKIDKR-YLVPNEMLNPPVRDPSRLNERSSTMAGAPFCCCGL
TaATG8_6A    LPRKMKPKLSLRETFYMIYGGNTIKELAELVCGR---------------L
TaATG8m_6DL  MPR--KSVFTLRETFYVLYGGTPVKELSELVCYR---------------L

AtATG8h      QLDPSKALFVFVHNTLPQTASRMD-------------------SLYNTFK
AtATG8i      HLSPGKALFVFVNNTLPQTAALMD-------------------SVYESYK
TaATG8i_5AL  HLSPGTALFVFVRNTLPQTANLMG-------------------SVYDSYK
```

```
TaATG8k_5DL  HLSPGTALFVFVRDTLPQTANLMG-------------------SVYDSYK
TaATG8j_5BL  HLSPGTALFVFVKNTLPQTGNLMG-------------------SVYDSYK
OsATG8d      HLSPGTALFVFVNNTLPQTAQLMG-------------------SVYESYK
TaATG8b_2AL  KLSAEKAIFIFVKNTLPPTAALMS-------------------AIYEENK
TaATG8b_2BL  KLSAEKAIFIFVKNTLPPTAALMS-------------------AIYEENK
TaATG8e_2DL  KLSAEKAIFIFVKNTLPPTAALMS-------------------AIYEENK
OsATG8b      KLSPEKAIFIFVKNTLPPTAALMS-------------------AIYEENK
OsATG8c      KLSAEKAIFIFVKNTLPPTAALMS-------------------AIYEENK
AtATG8c      KLSAEKAIFVFVKNTLPPTAAMMS-------------------AIYDENK
AtATG8d      KLSPEKAIFIFVKNILPPTAAIMS-------------------AIYEEHK
TaATG8c_2AS  KLSPEKAIFVFVNSTLPPTASLMS-------------------AIYEENK
TaATG8d_2BS  KLSPEKAIFVFVNSTLPPTASLMS-------------------AIYEENK
TaATG8f_2DS  KLSPEKAIFVFVNSTLPPTASLMS-------------------AIYEENK
OsATG8a      KLSPEKAIFVFVKNTLPPTASLMS-------------------AIYEENK
AtATG8a      KLGAEKAIFVFVKNTLPPTAALMS-------------------AIYEEHK
AtATG8b      KLGAEKAIFVFVKNTLPPTAALMS-------------------AIYEEHK
AtATG8e      KLSAEKAIFIFVDNVLPPTGELMS-------------------SVYEDKK
AtATG8f      KLSAEKAIFIFVDNVLPPAGALMS-------------------SVYEEKK
AtATG8g      QLSAEKAIFIFVDNVLPPTGAMMS-------------------TIYDENK
ScATG8       MLPPEKAIFIFVNDTLPPTAALMS-------------------AIYQEHK
TaATG8l_6BS  KPAPPSFLLCHGSSRLPHKHGMSPRPTSTRPLSPVRPCHCLCYPMAAHCP
TaATG8_6A    NLEVGHPMKFTIDGTLLPEDCVLS-------------------SVYNKYI
TaATG8m_6DL  NLEEGRPMQFLMNGDLLAEATTVS-------------------AIYEKHL

AtATG8h      EE-DGFLYMCYSTEKT------FG--------------------------
AtATG8i      DD-DGFVYMCYSSEKT------FG--------------------------
TaATG8i_5AL  DKQDGFLYMCYSSEKT------FG--------------------------
TaATG8k_5DL  DKQDGFLYMCYSSEKT------FG--------------------------
TaATG8j_5BL  DKQDGFLYMCYSNEKT------FG--------------------------
OsATG8d      DEGDGFLYLCYSSEKT------FG--------------------------
TaATG8b_2AL  DE-DGFLYMTYSGENT------FGLL------------------------
TaATG8b_2BL  DE-DGFLYMTYSGENT------FGLL------------------------
TaATG8e_2DL  DE-DGFLYMTYSGLSSP-----FRFC------------------------
OsATG8b      DE-DGFLYMTYSGENT------FGLL------------------------
OsATG8c      DE-DGFLYMTYSGENT------FGLFV-----------------------
AtATG8c      DE-DGFLYMTYSGENT------FGLV------------------------
AtATG8d      DE-DGFLYMSYSGENT------FGIFF-----------------------
TaATG8c_2AS  DE-DGFLYMTYSGENT------FGSA------------------------
TaATG8d_2BS  DE-DGFLYMTYSGENT------FGSA------------------------
TaATG8f_2DS  DE-DGFLYMTYSGENT------FGSA------------------------
OsATG8a      DE-DGFLYMTYSGENT------FGSA------------------------
AtATG8a      DE-DGFLYMTYSGENT------FGSLTVA---------------------
AtATG8b      DE-DGFLYMTYSGENT------FGGSFYC---------------------
AtATG8e      DE-DGFLYITYSGENT------FGASSI----------------------
AtATG8f      DD-DGFLYVTYSGENT------FGFGSP----------------------
AtATG8g      EE-DGFLYVTYSGENT------FGSSMT----------------------
ScATG8       DK-DGFLYVTYSGENT------FGR-------------------------
TaATG8l_6BS  EPRHGRASPPLAGPPPS-----TSSAWLVRLCLGHRPQPCLAYRASPRHR
TaATG8_6A    WD-GSILTLCCCVDN---VKKIFNVDLSVKVPVIPPVHVIEDEVPLPTLR
TaATG8m_6DL  LS-DNLATLQCYIDKKKFVPRSFNVDKSVKVPIVEPVYVNEEYIPFPPFR

AtATG8h      -------------------------------------------
AtATG8i      -------------------------------------------
```

```
TaATG8i_5AL  -------------------------------------------
TaATG8k_5DL  -------------------------------------------
TaATG8j_5BL  -------------------------------------------
OsATG8d      -------------------------------------------
TaATG8b_2AL  -------------------------------------------
TaATG8b_2BL  -------------------------------------------
TaATG8e_2DL  -------------------------------------------
OsATG8b      -------------------------------------------
OsATG8c      -------------------------------------------
AtATG8c      -------------------------------------------
AtATG8d      -------------------------------------------
TaATG8c_2AS  -------------------------------------------
TaATG8d_2BS  -------------------------------------------
TaATG8f_2DS  -------------------------------------------
OsATG8a      -------------------------------------------
AtATG8a      -------------------------------------------
AtATG8b      -------------------------------------------
AtATG8e      -------------------------------------------
AtATG8f      -------------------------------------------
AtATG8g      -------------------------------------------
ScATG8       -------------------------------------------
TaATG8l_6BS  LPGRPAPACRRLTPLVLL-------------------------
TaATG8_6A    ICSVRDARRASMMSKVGTYSLKDGAPAVLVHGTYGEGGSLVFY
TaATG8m_6DL  IRNIRDAKKAAADARSGSY-----GNATISHGSYSEEGHLQFY
;
end;
```

ATG8 sequences

```
>TaATG8i_5AL TraesCS5A03G0430400
MKSFKKEFTLEERANESAAMIAKYPGRIPVIVERFSRSKLPEMEKRKYLVPCDMPVGQFIFILRSRLHLSPGTALFV
FVRNTLPQTANLMGSVYDSYKDKQDGFLYMCYSSEKTFG

>TaATG8j_5BL TraesCS5B03G0425000
MKSFKKEFTLEERANESAAMIAKYPGRIPVIVERFSRSNLPEMEKRKYLVPCDMLVGQFIFILRSRLHLSPGTALFV
FVKNTLPQTGNLMGSVYDSYKDKQDGFLYMCYSNEKTFG

>TaATG8k_5DL TraesCS5D03G0399300
MDAEERANESAAMIAKYPGRIPVIVERFSRSNLPEMEKRKYLVPCDMPVGQFIFILRSRLHLSPGTALFVFVRDTLP
QTANLMGSVYDSYKDKQDGFLYMCYSSEKTFG

>TaATG8_6A TraesCS6A03G1019400
MVRPLEVRFVPLSGFAPGGDPPQGPRAKKVSKAAPVFEVPGLWTPDVPLENAYRDCINMTVVYLDLHLPRKMKPKLS
LRETFYMIYGGNTIKELAELVCGRLNLEVGHPMKFTIDGTLLPEDCVLSSVYNKYIWDGSILTLCCCVDNVKKIFNV
DLSVKVPVIPPVHVIEDEVPLPTLRICSVRDARRASMMSKVGTYSLKDGAPAVLVHGTYGEGGSLVFY

>TaATG8l_6BS TraesCS6B03G0572700
MAKTCFKTEHPLERRQAESARIREKYADRIPVIVEKADKSDLPKIDKRYLVPNEMLNPPVRDPSRLNERSSTMAGAP
FCCCGLKPAPPSFLLCHGSSRLPHKHGMSPRPTSTRPLSPVRPCHCLCYPMAAHCPEPRHGRASPPLAGPPPSTSSA
WLVRLCLGHRPQPCLAYRASPRHRLPGRPAPACRRLTPLVLL


>TaATG8m_6DL TraesCS6D03G0897600
```

MVRPFEDDYLCPAWFAPGGVRATHSKDGSTKRSSKDRSCSPFYIPGLRTADVAVVEEYLRSVNIMPVFLDIKMPRKS
VFTLRETFYVLYGGTPVKELSELVCYRLNLEEGRPMQFLMNGDLLAEATTVSAIYEKHLLSDNLATLQCYIDKKKFV
PRSFNVDKSVKVPIVEPVYVNEEYIPFPPFRIRNIRDAKKAAADARSGSYGNATISHGSYSEEGHLQFY

>TaATG8c_2AS TraesCS2A03G0482400
MAKTCFKTEHPLERRQAESARIREKYADRIPVIVEKADKSDVPEIDKKKYLVPADLTVGQFVYVVRKRIKLSPEKAI
FVFVNSTLPPTASLMSAIYEENKDEDGFLYMTYSGENTFGSA*

>TaATG8b_2AL TraesCS2A03G1101500
MAKSSFKLEHPLERRQAEATRIREKYSDRIPVIVEKAGKSDIPDIDKKKYLVPADLTVGQFVYVVRKRIKLSAEKAI
FIFVKNTLPPTAALMSAIYEENKDEDGFLYMTYSGENTFGLL

>TaATG8d_2BS TraesCS2B03G0601600
MAKTCFKTEHPLERRQAESARIREKYADRIPVIVEKADKSDVPEIDKKKYLVPADLTVGQFVYVVRKRIKLSPEKAI
FVFVNSTLPPTASLMSAIYEENKDEDGFLYMTYSGENTFGSA

>TaATG8b_2BL TraesCS2B03G1237500
MAKSSFKLEHPLERRQAEANRIREKYSDRIPVIVEKAGKSDIPDIDKKKYLVPADLTVGQFVYVVRKRIKLSAEKAI
FIFVKNTLPPTAALMSAIYEENKDEDGFLYMTYSGENTFGLL

>TaATG8f_2DS TraesCS2D03G0485800
MAKTCFKTEHPLERRQAESARIREKYADRIPVIVEKADKSDVPEIDKKKYLVPADLTVGQFVYVVRKRIKLSPEKAI
FVFVNSTLPPTASLMSAIYEENKDEDGFLYMTYSGENTFGSA

>TaATG8e_2DL (TRIAE_CS42_2DL_TGACv1_157983_AA0504720.1.1, Yue et al., 2018)
PIERRQAEANRIREKYSDRIPVIVEKAGKSDIPDIDKKKYLVPADLTVGQFVYVVRKRIKLSAEKAIFIFVKNTLPP
TAALMSAIYEENKDEDGFLYMTYSGLSSPFRFC
