# Supplementary material for: Genotype-Specific Activation of Autophagy during Heat Wave in Wheat
Source: Cells. 2024 Jul 20;13(14):1226. doi: 10.3390/cells13141226 (PMC11274669; doi:10.3390/cells13141226)

Supplemental Dataset 5. Heatmaps of all differentially expressed autophagy genes.

A. Expression patterns of autophagy DEGs in GSE57950_h471

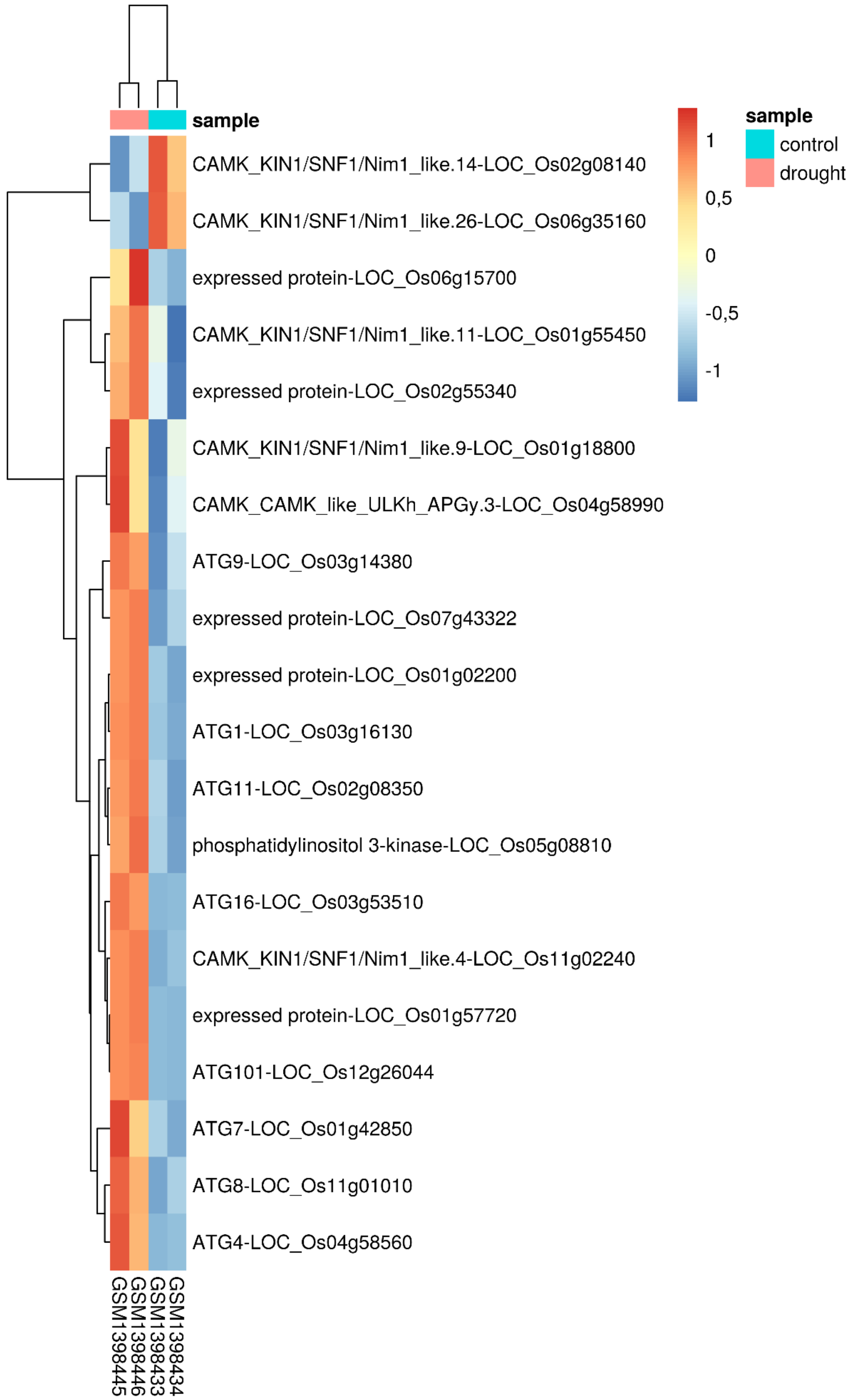

B. Expression patterns of autophagy DEGs in GSE57950_hhz

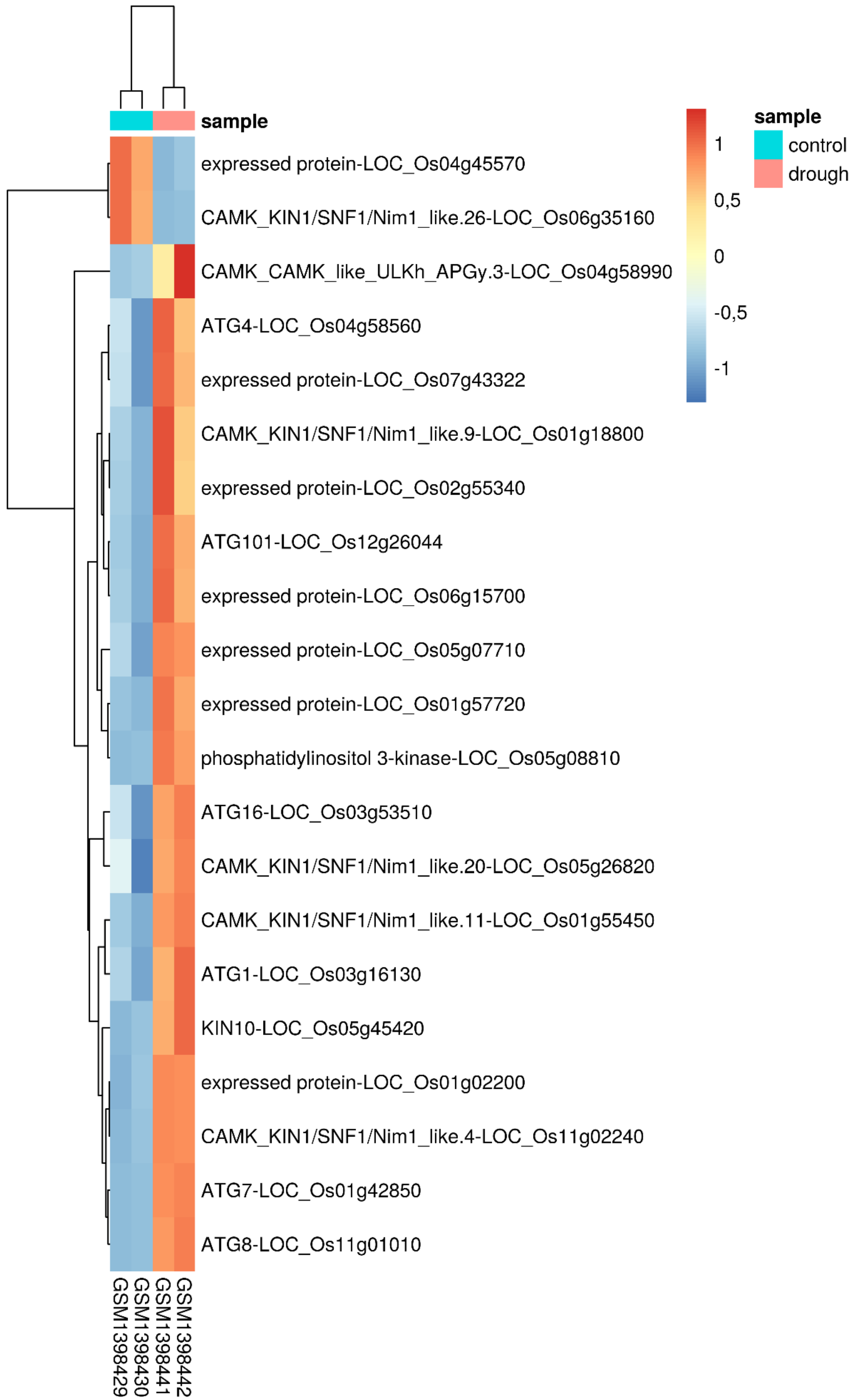

C. Expression patterns of autophagy DEGs in GSE57950_p28

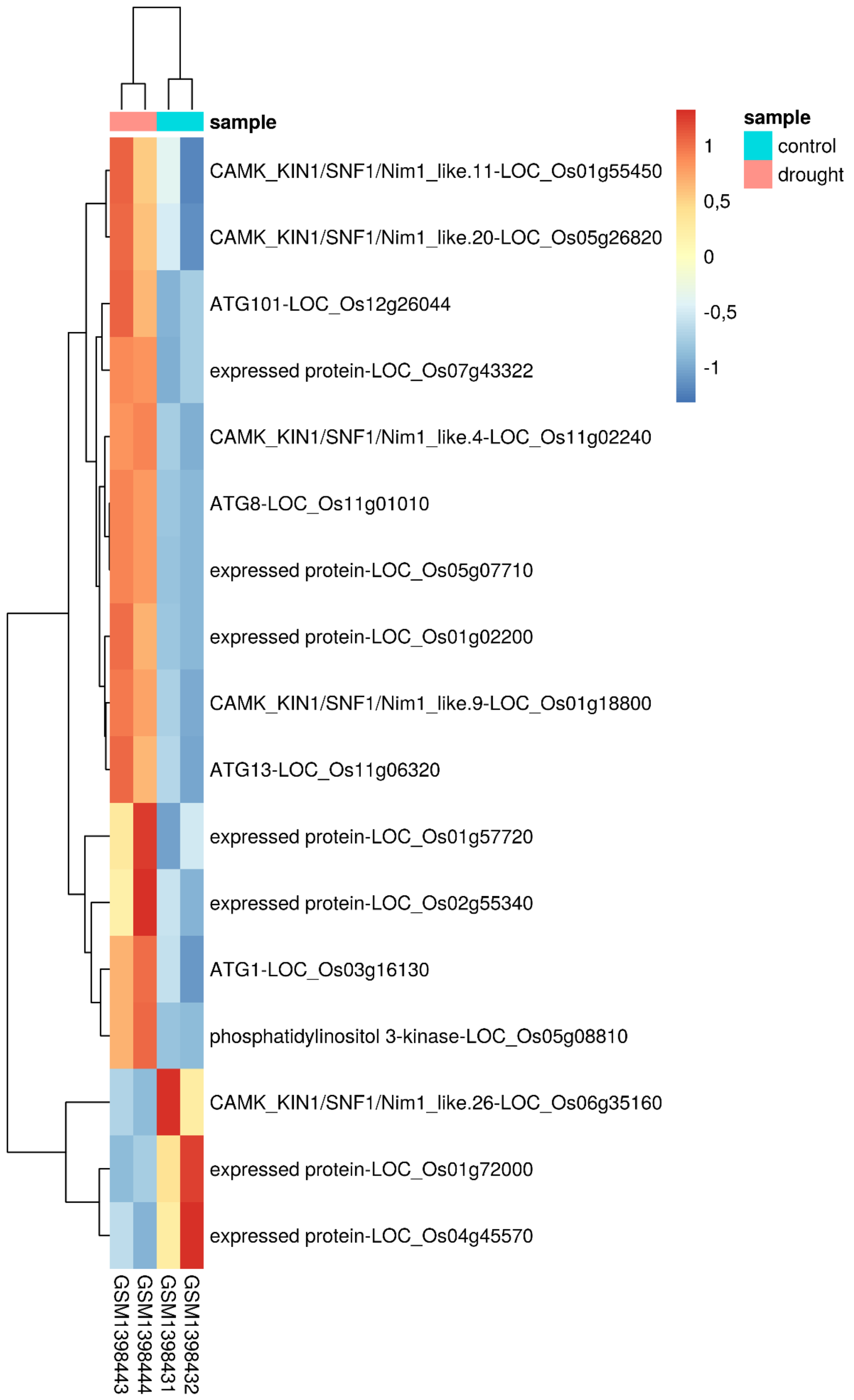

D. Expression patterns of autophagy DEGs in GSE65022

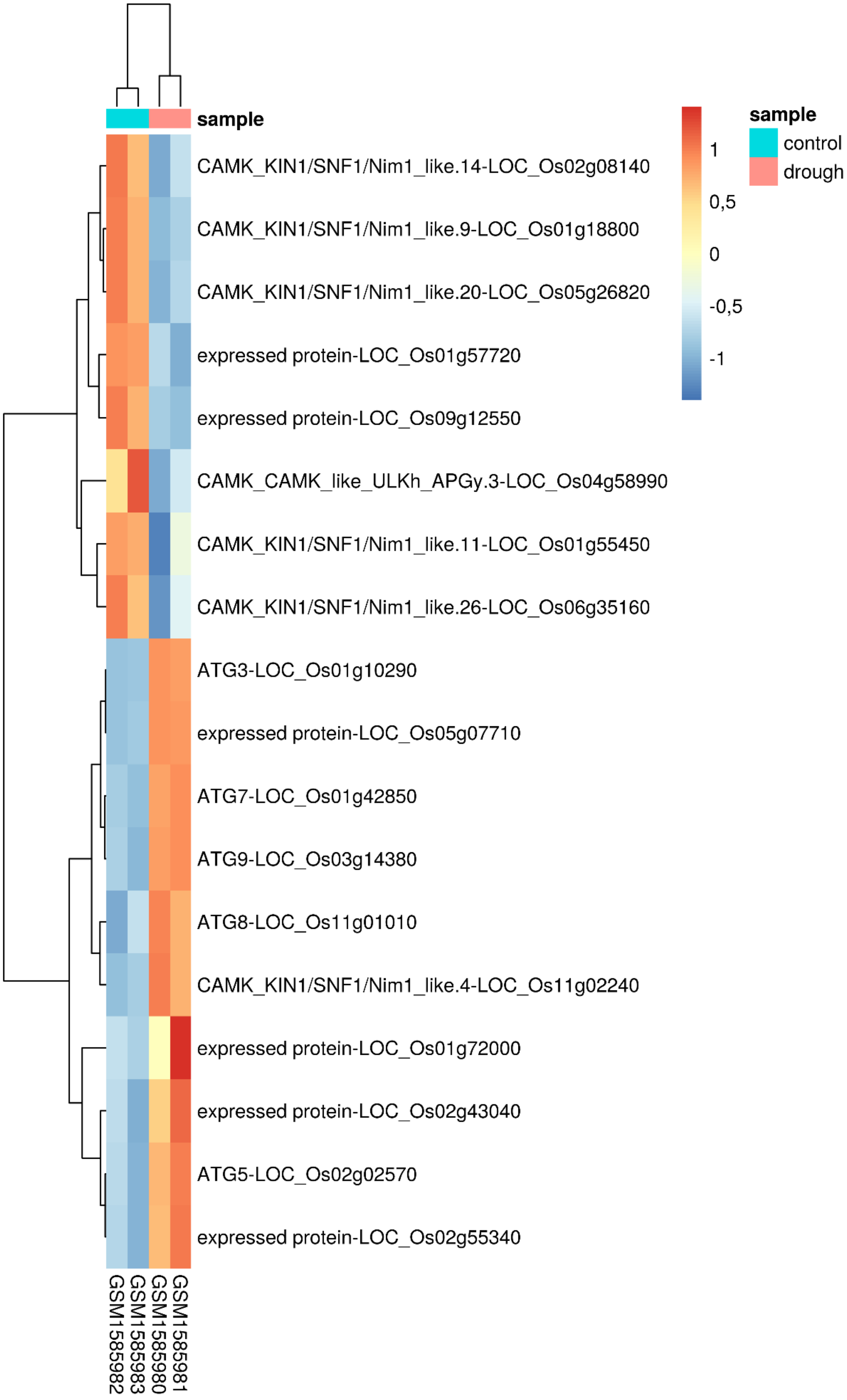

E. Expression patterns of autophagy DEGs in GSE78972_long

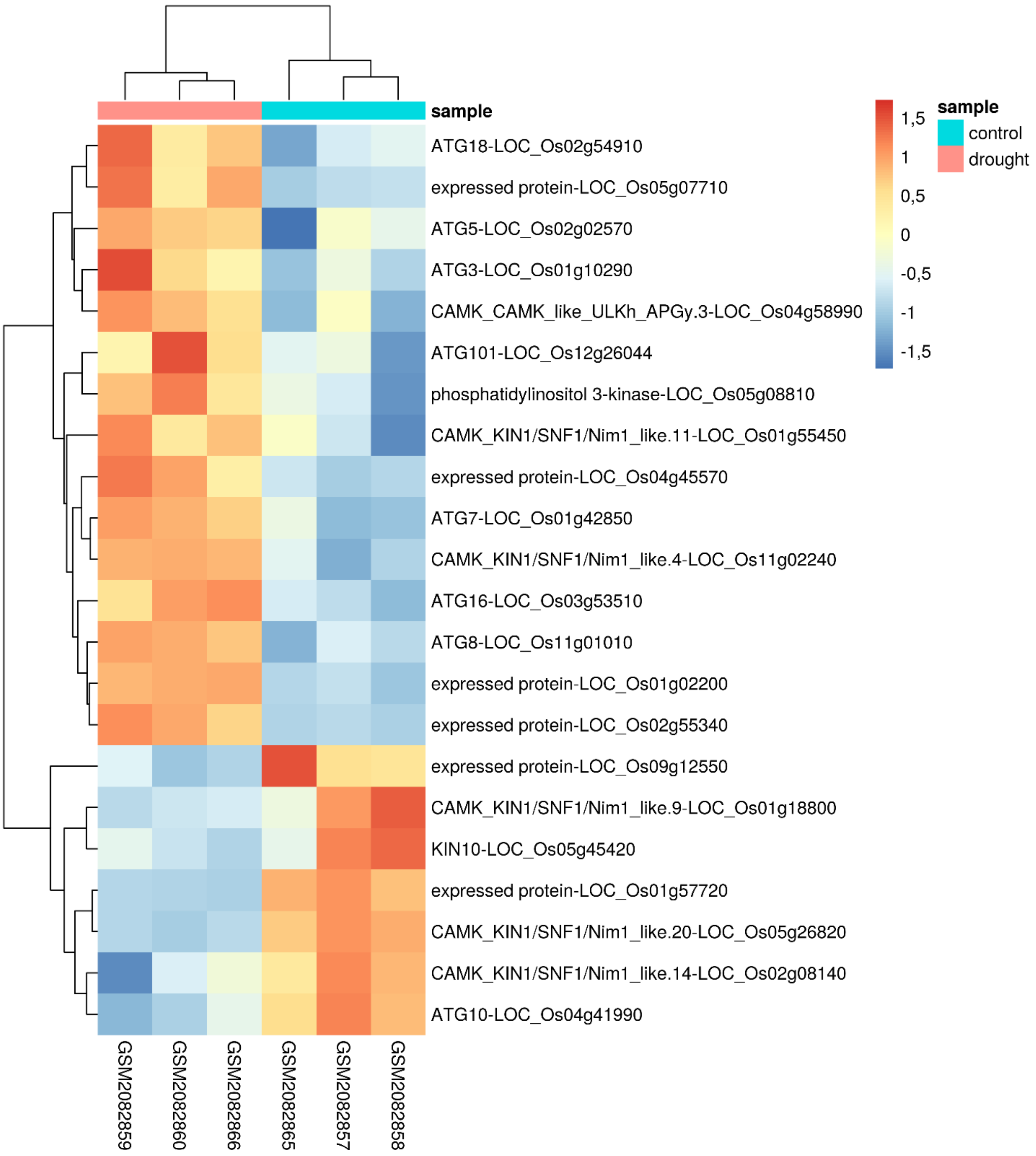

F. Expression patterns of autophagy DEGs in GSE78972_short

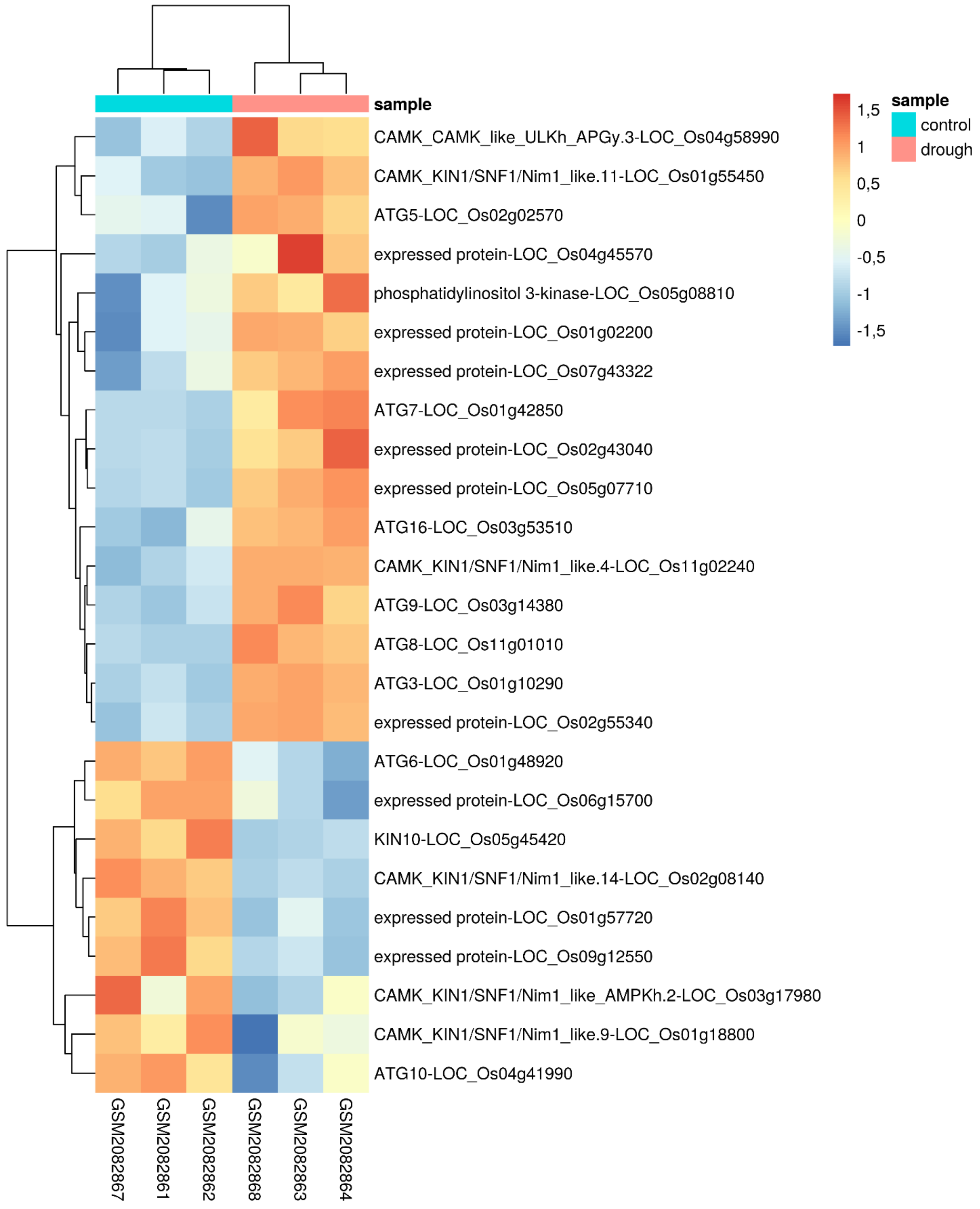

G. Expression patterns of autophagy DEGs in GSE48507

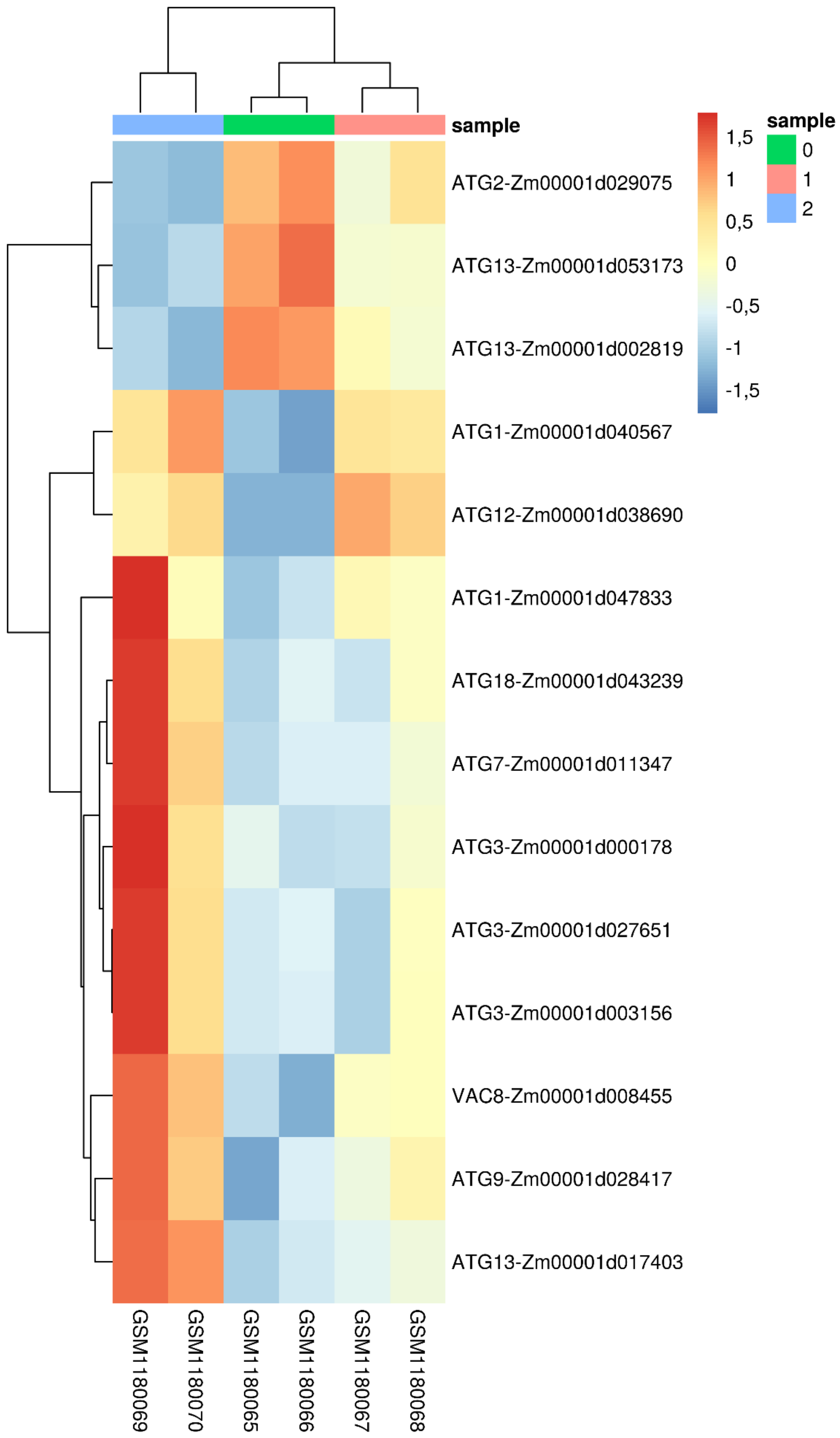

H. Expression patterns of autophagy DEGs in GSE132113

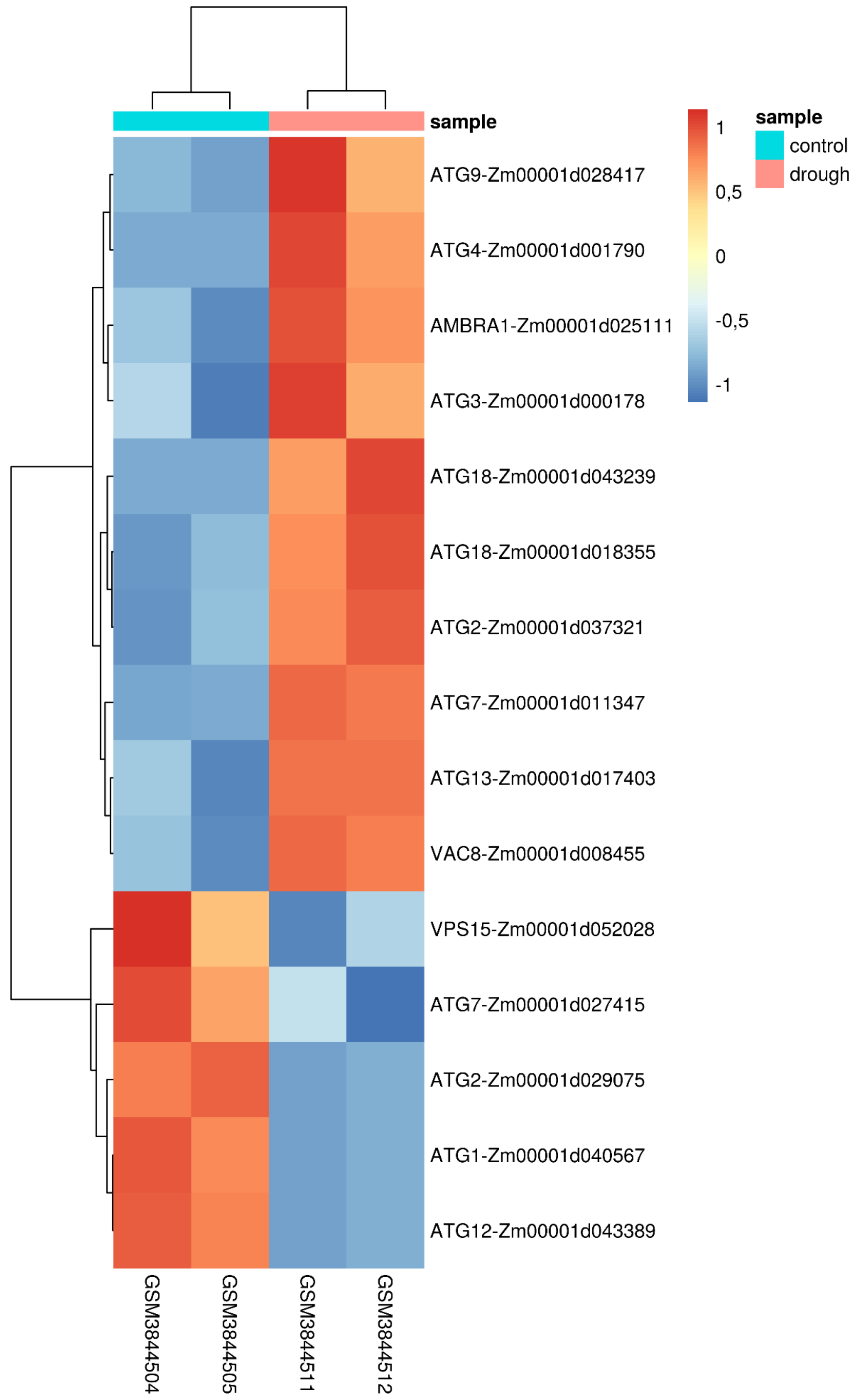

I. Expression patterns of autophagy DEGs in GSE137780_b104

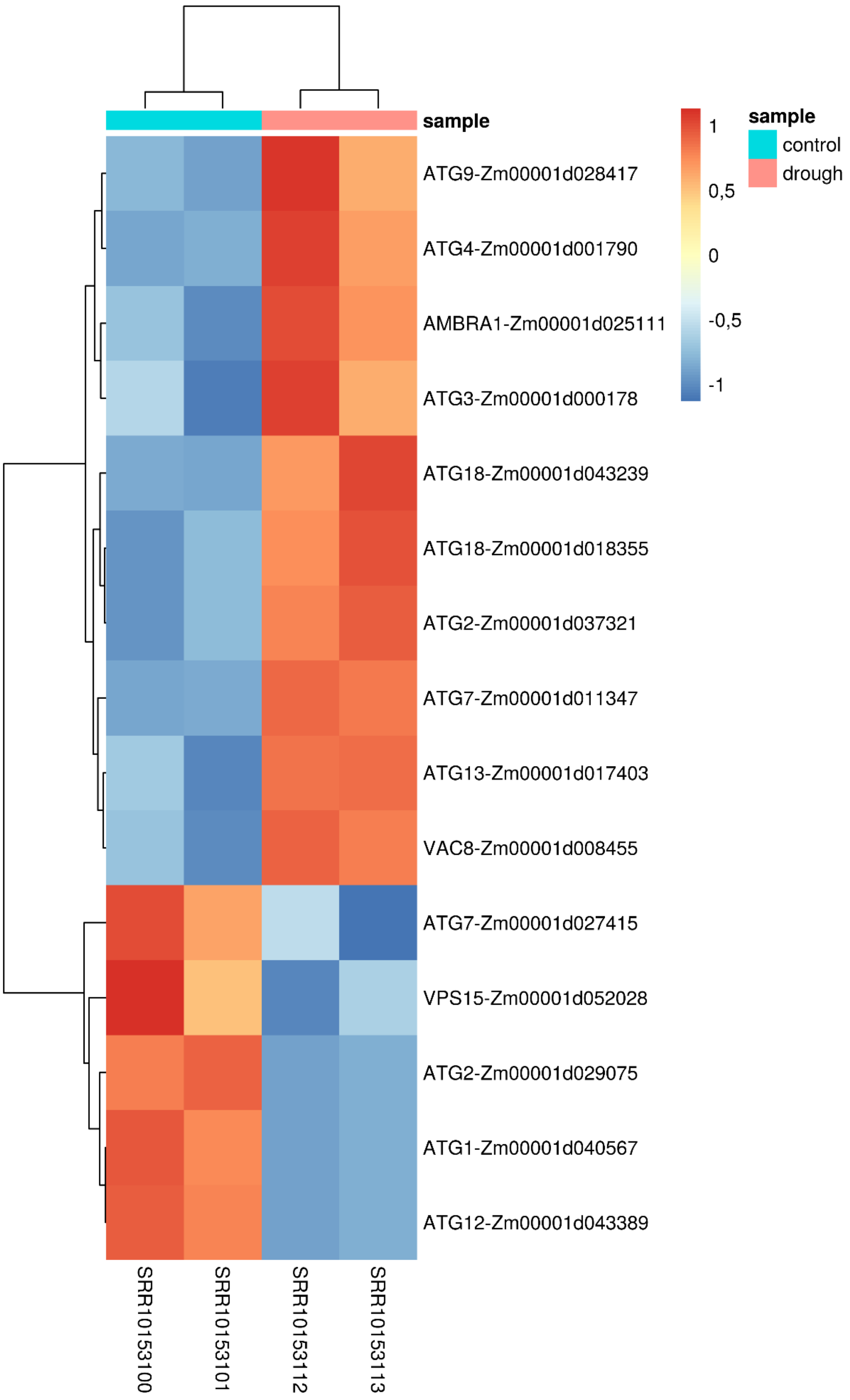

J. Expression patterns of autophagy DEGs in GSE137780_DH4866

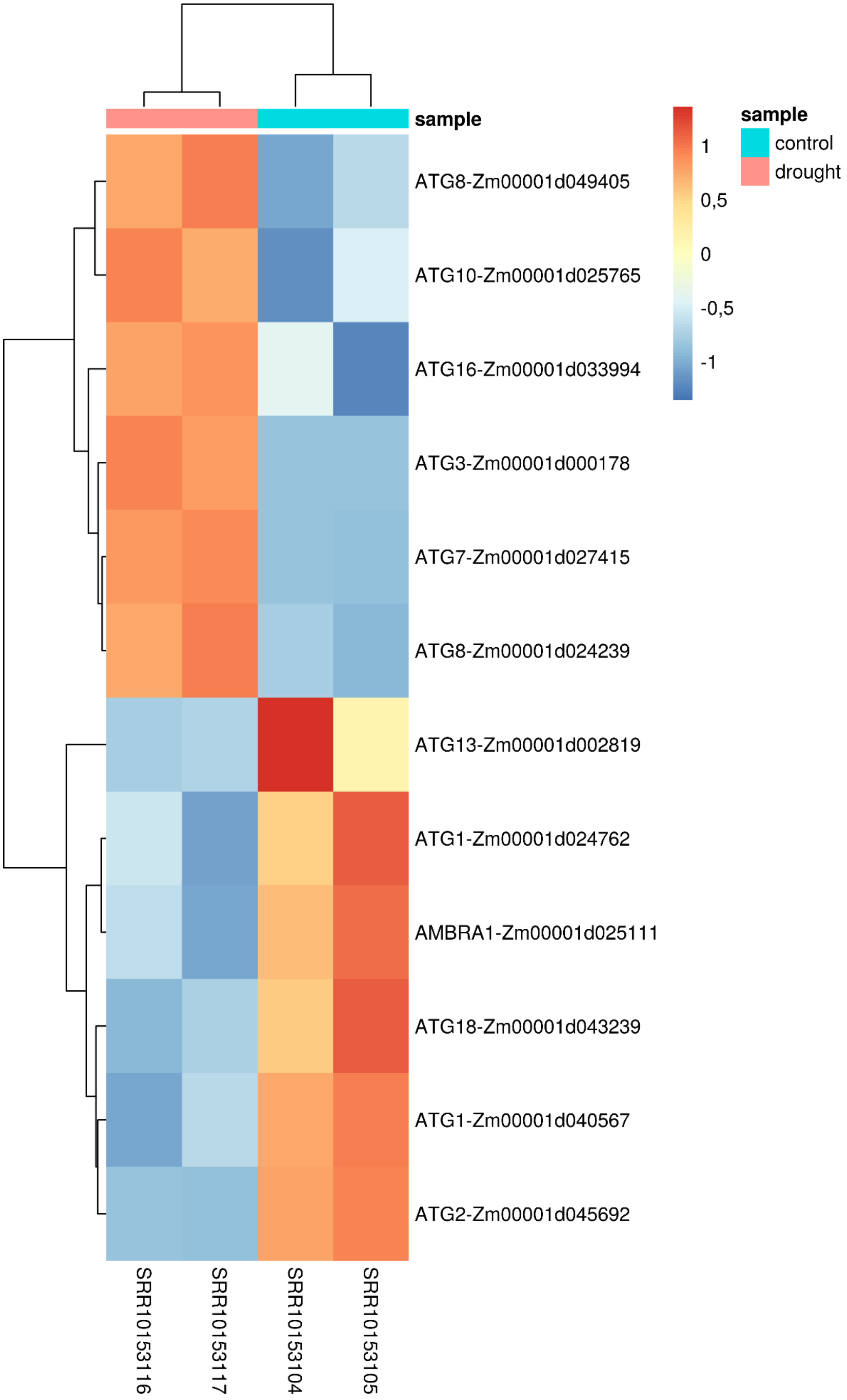

K. Expression patterns of autophagy DEGs in GSE137780_W22

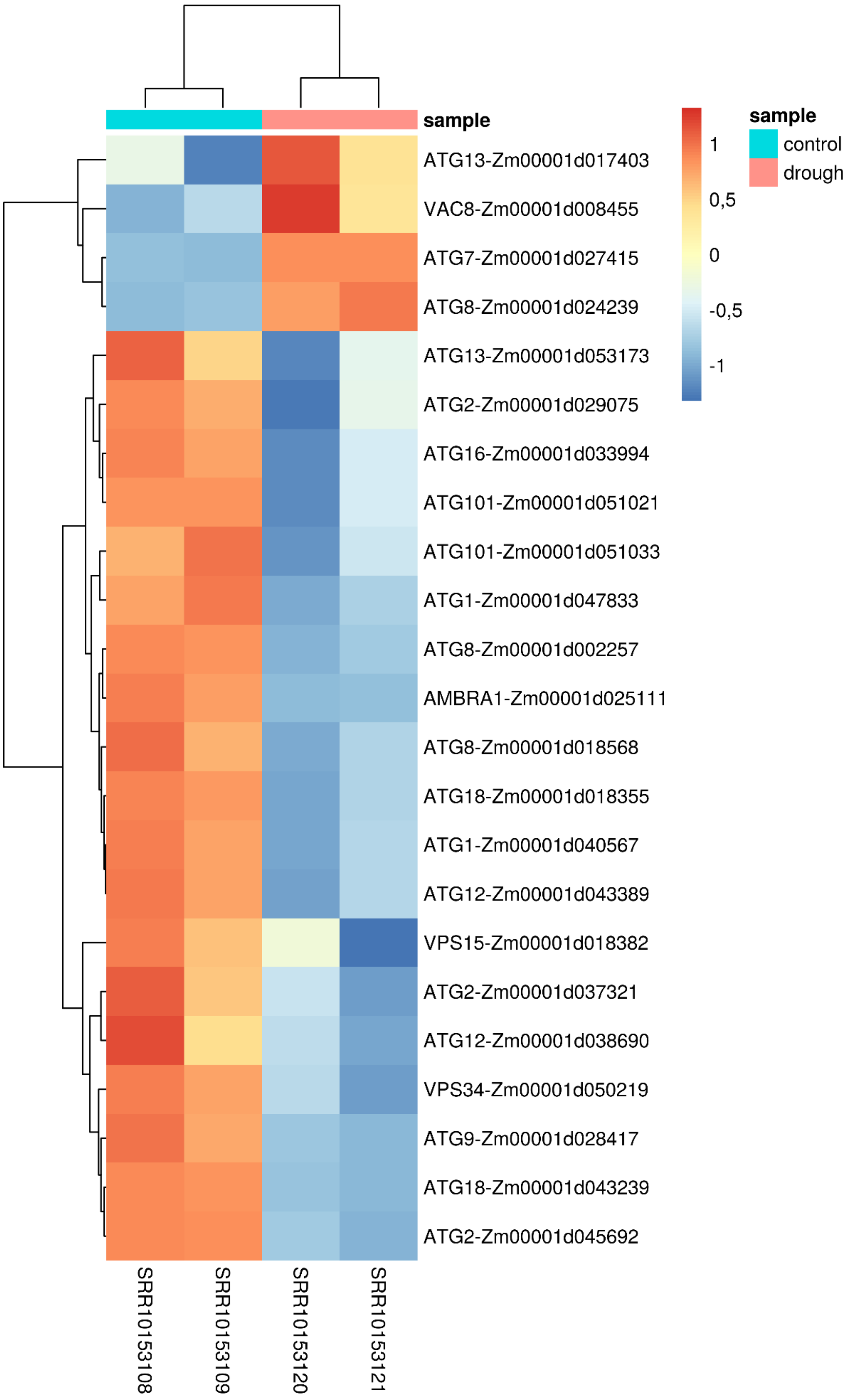

L. Expression patterns of autophagy DEGs in GSE80699_ IS20351

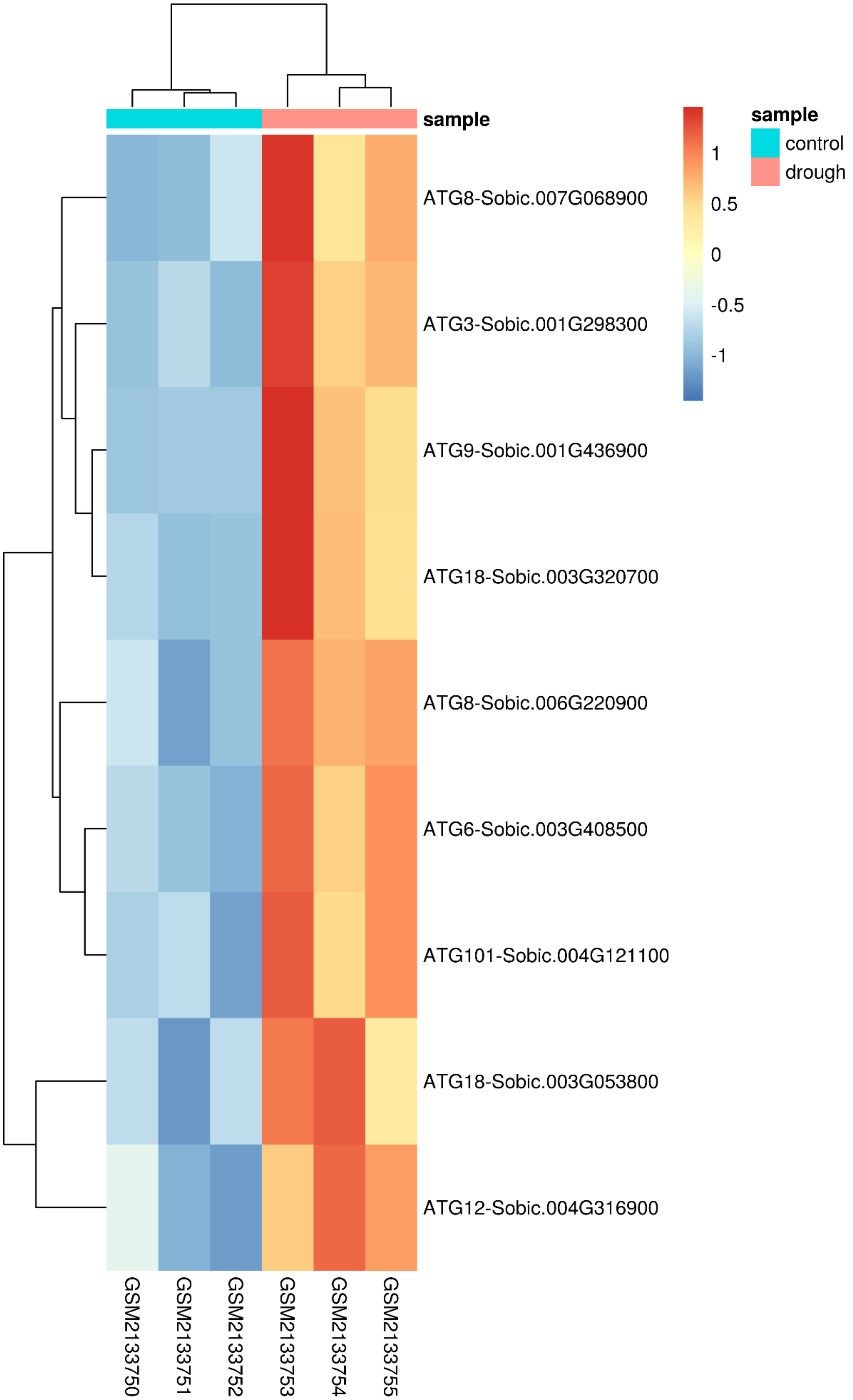

M. Expression patterns of autophagy DEGs in GSE80699_IS22330

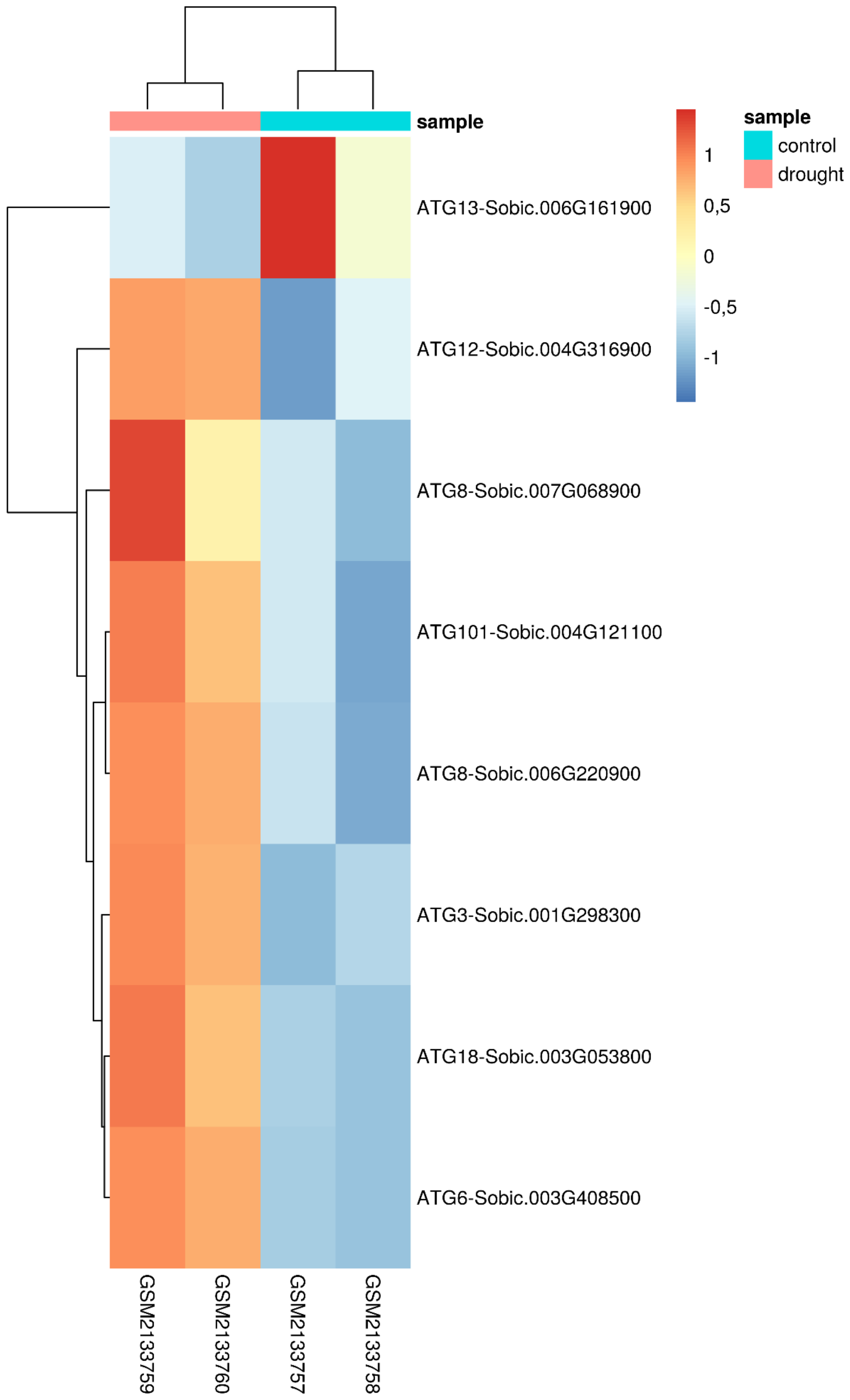

N. Expression patterns of autophagy DEGs in GSE97776_Gwiazda

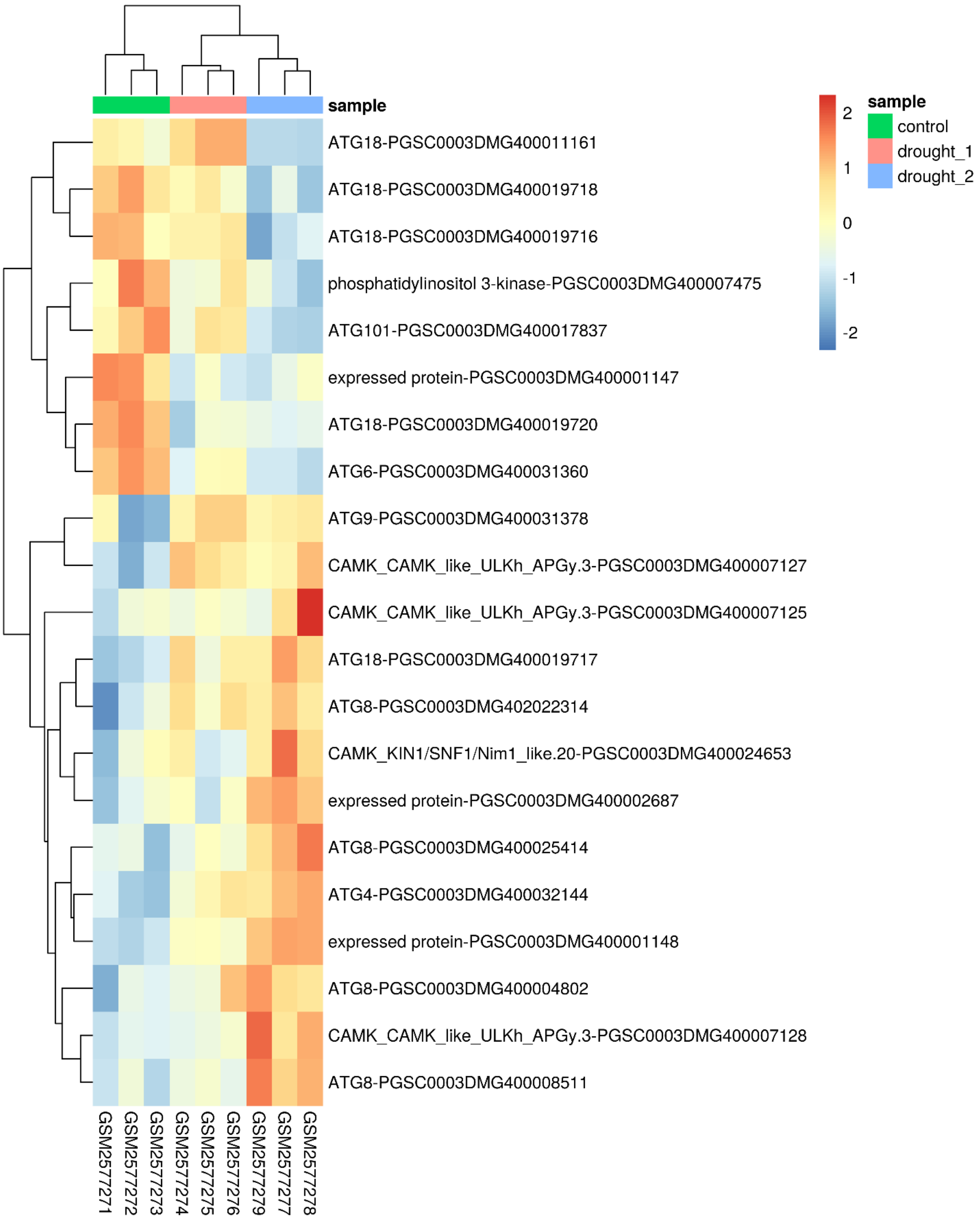

O. Expression patterns of autophagy DEGs in GSE97776_Oberon

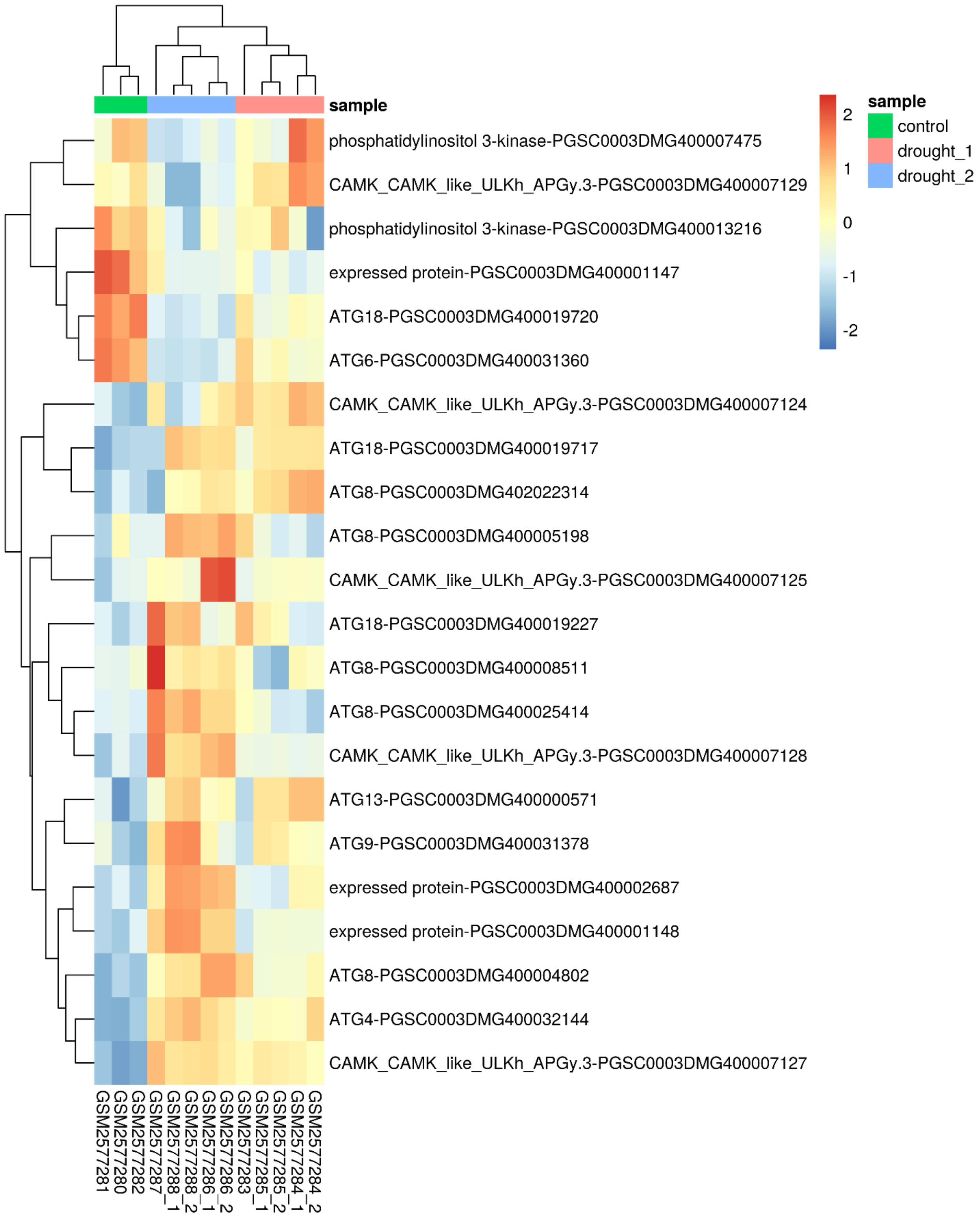

P. Expression patterns of autophagy DEGs in GSE97776_Owacja

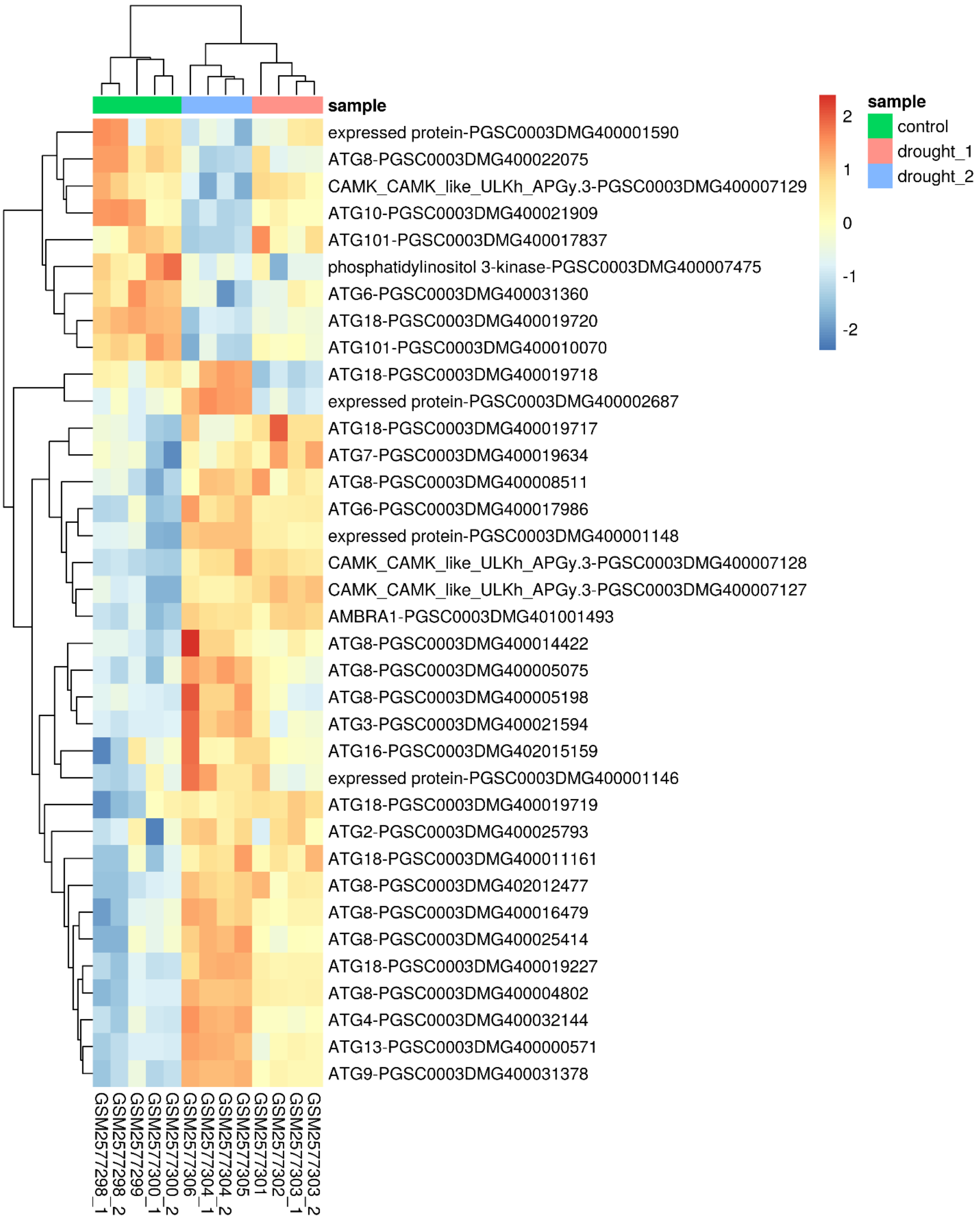

Q. Expression patterns of autophagy DEGs in GSE97776_Tajfun

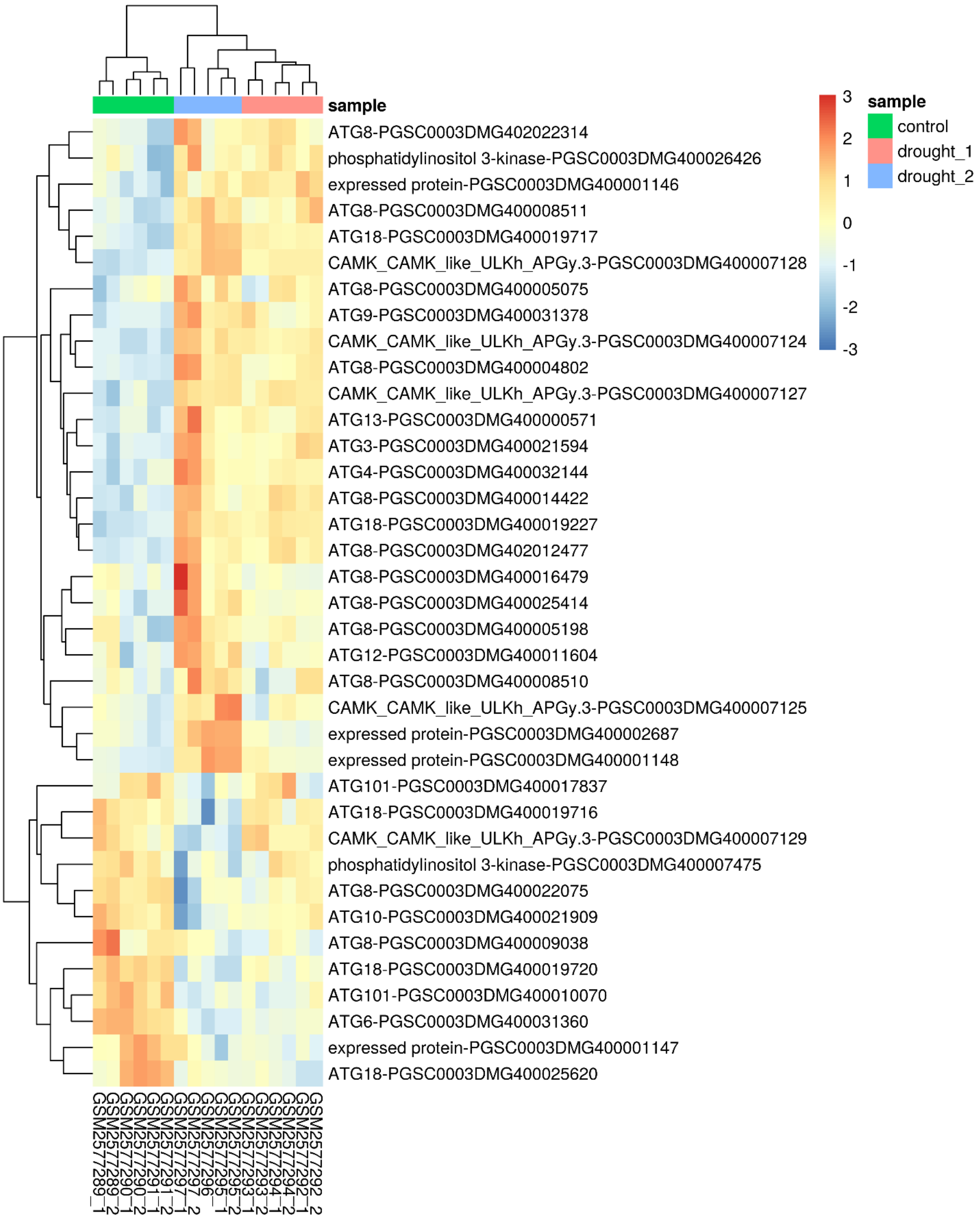

R. Expression patterns of autophagy DEGs in GSE151277_day1

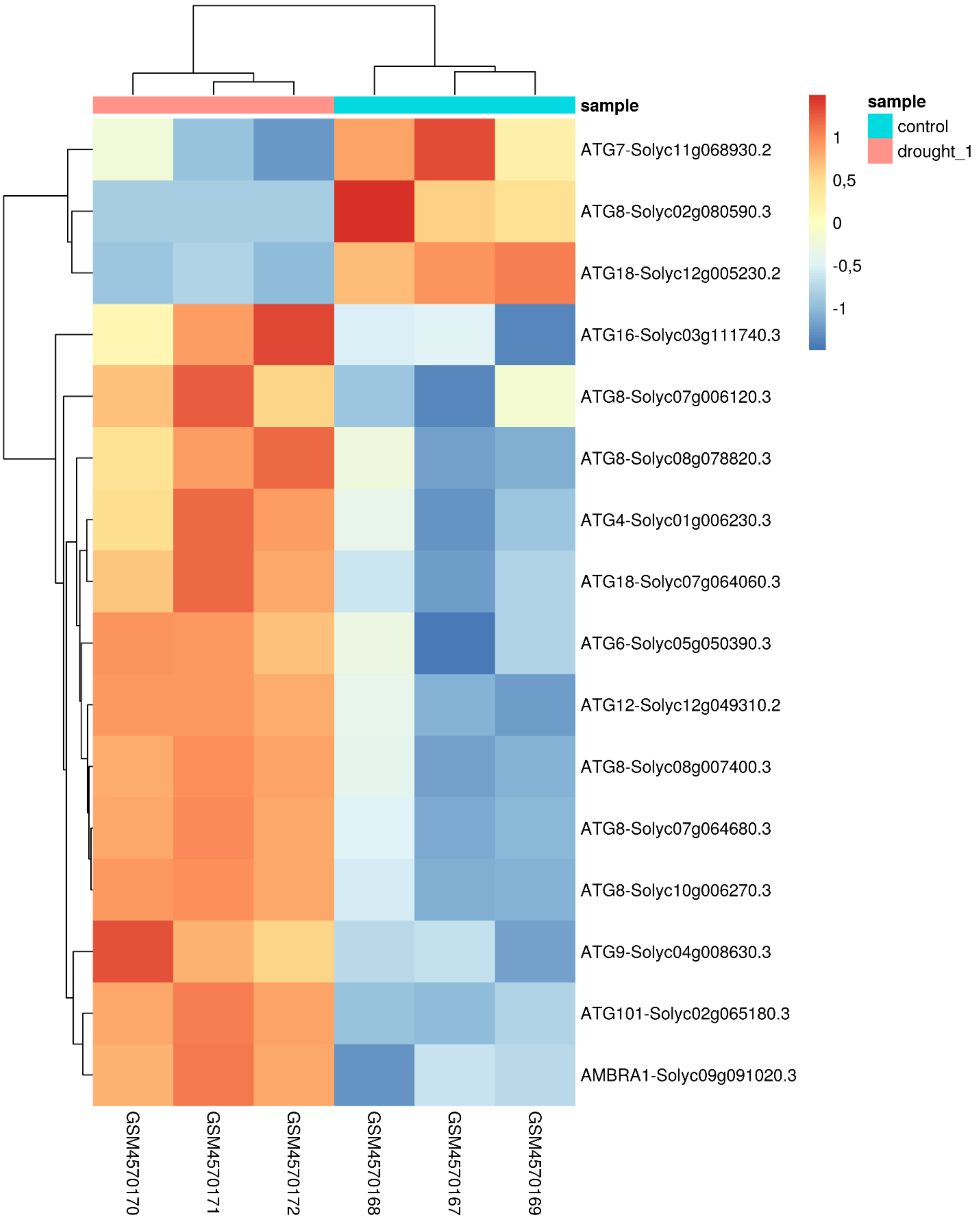

S. Expression patterns of autophagy DEGs in GSE151277_day2

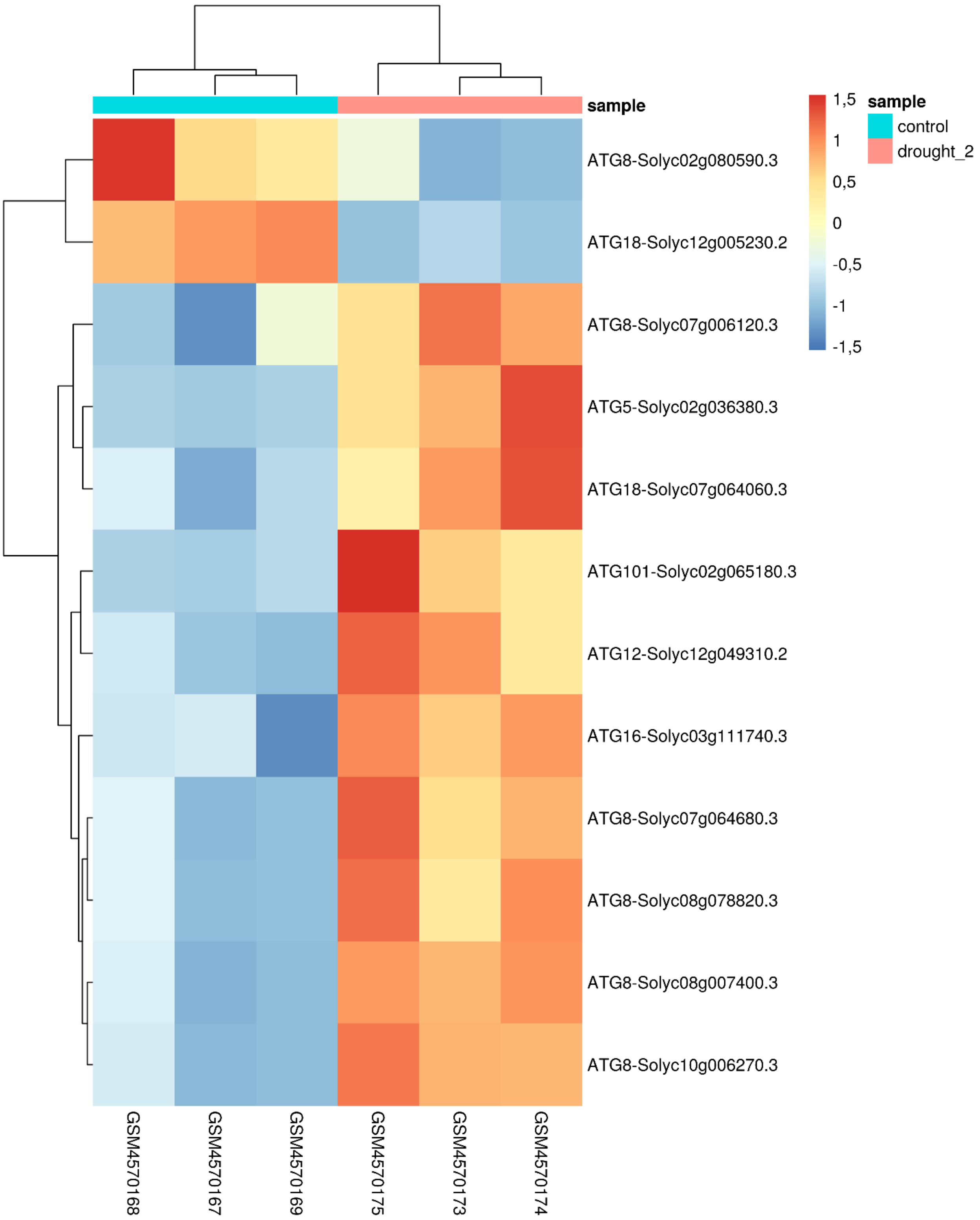

T. Expression patterns of autophagy DEGs in GSE151277_day3

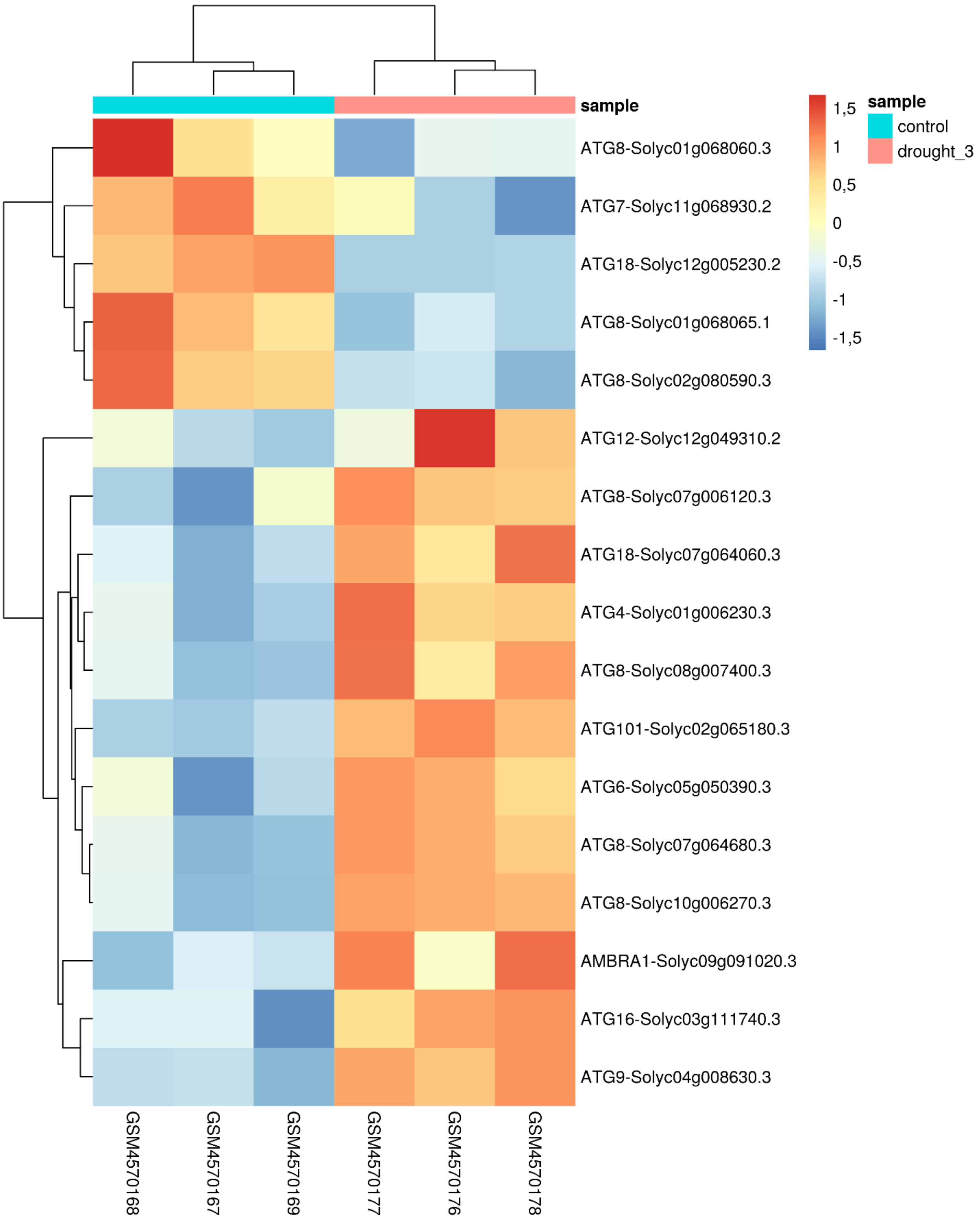

U. Expression patterns of autophagy DEGs in GSE151277_day4

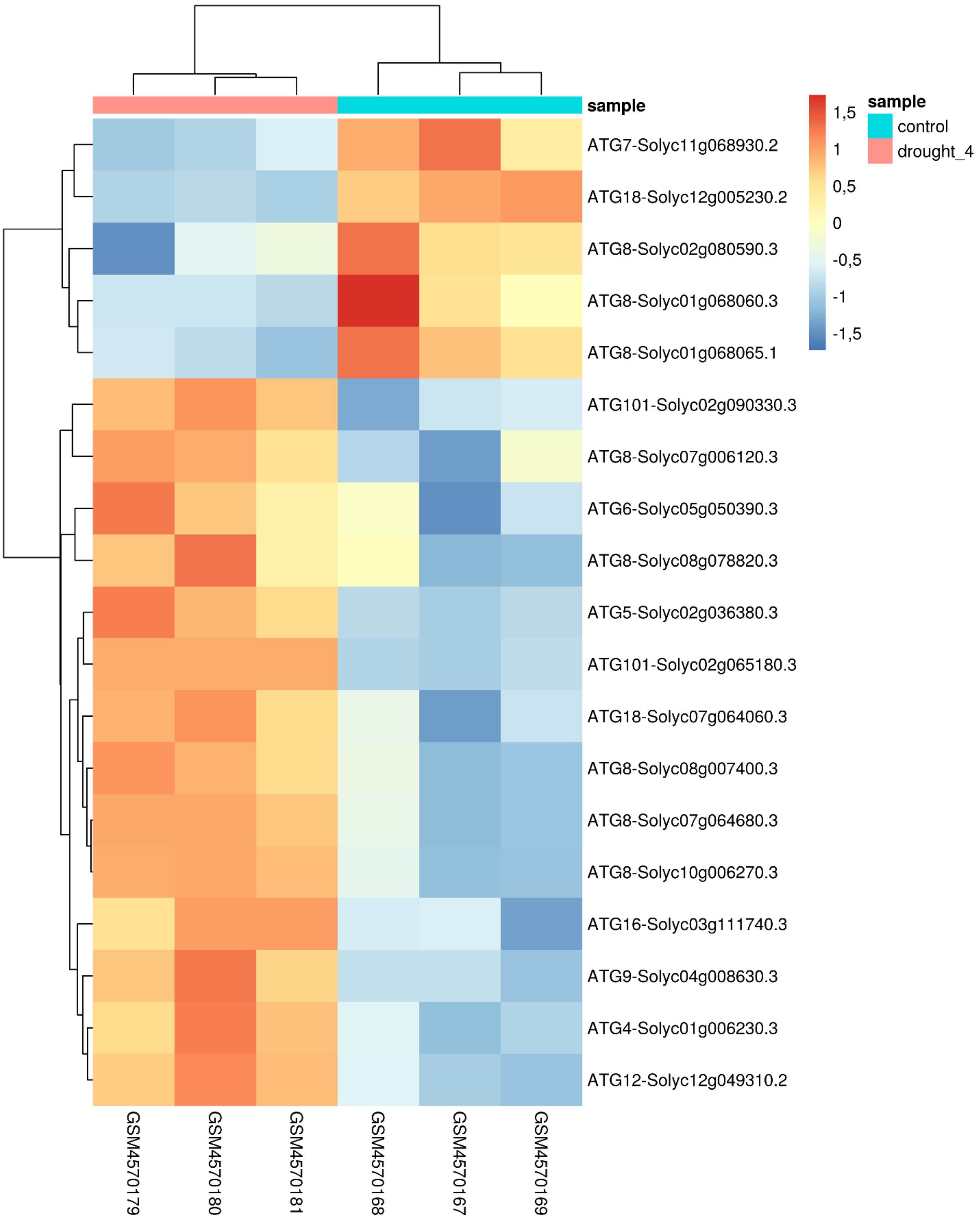

V. Expression patterns of autophagy DEGs in GSE151277_day5

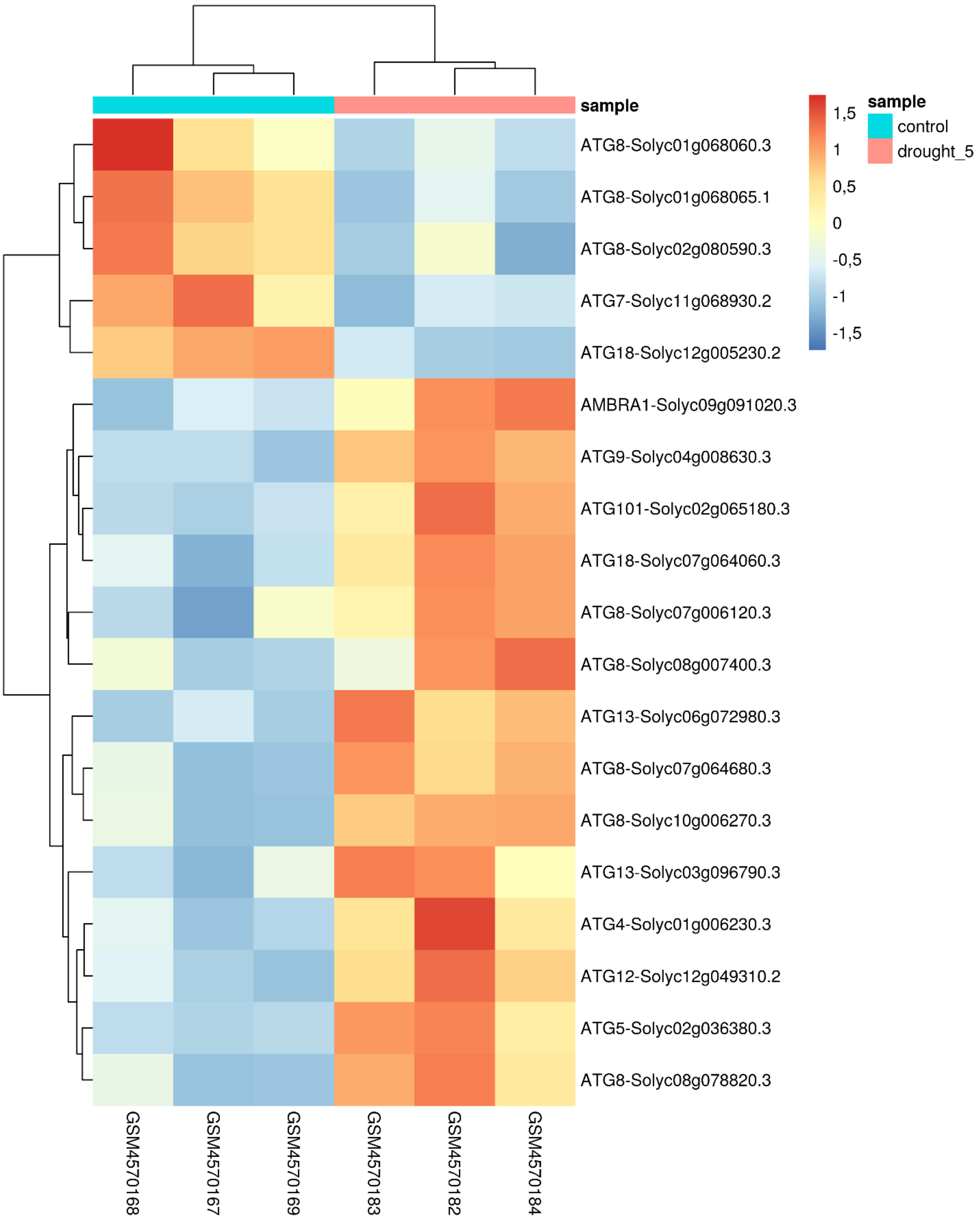

W. Expression patterns of autophagy DEGs in GSE134945

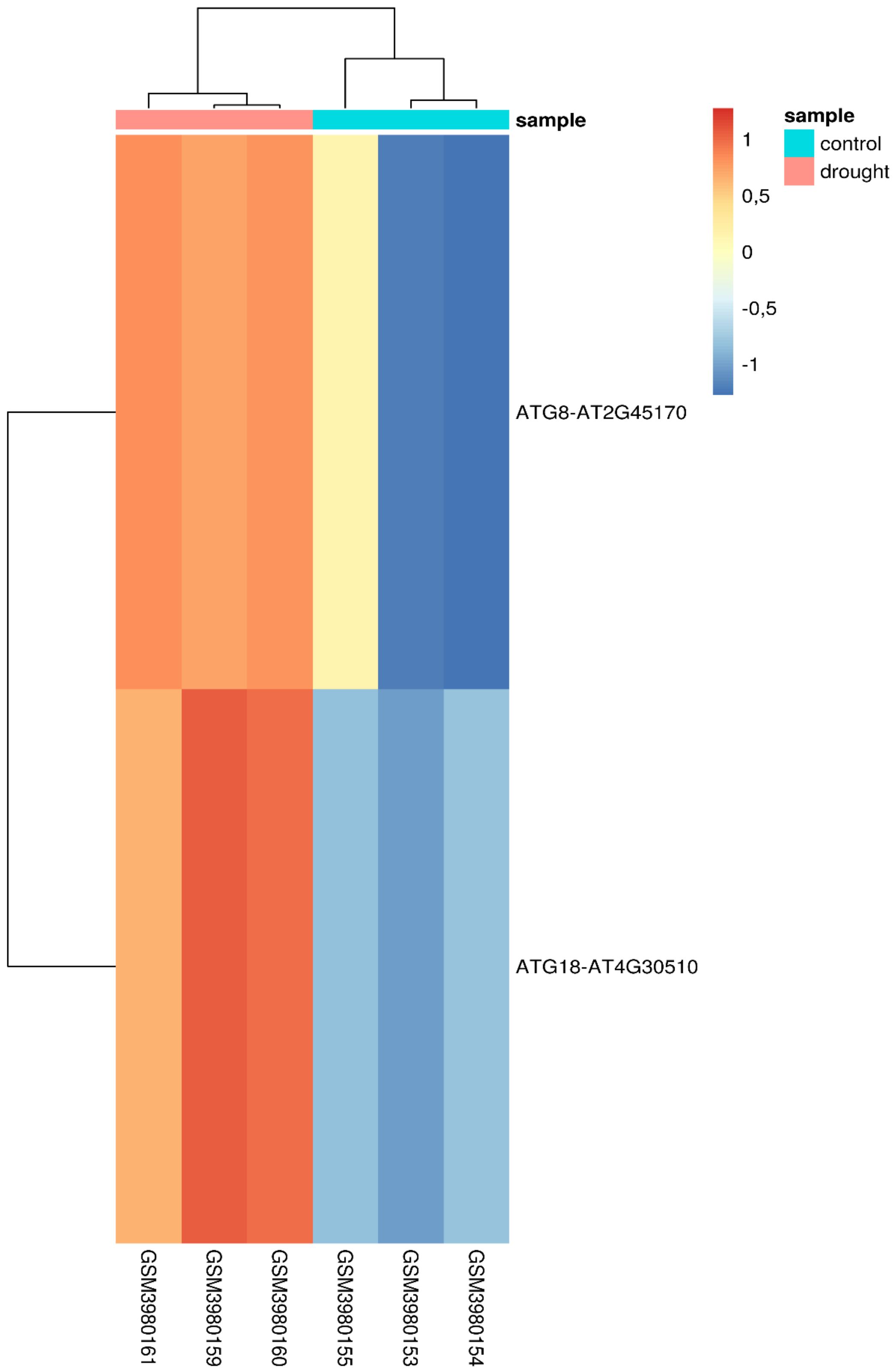

X. Expression patterns of autophagy DEGs in GSE93979

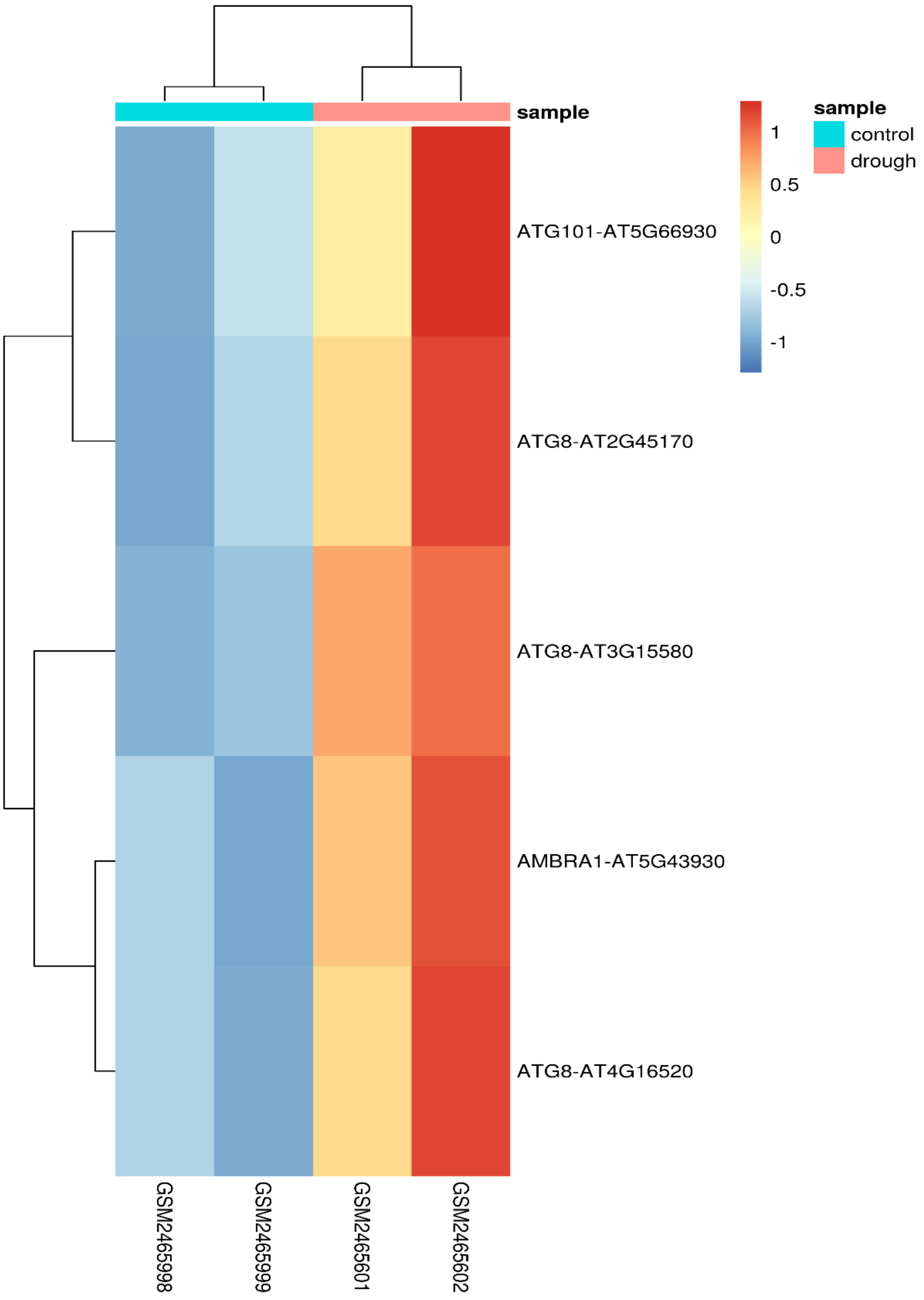

Supplement: Supplementary file 1 [file cells-13-01226-s001.zip › Supplemental Dataset 5.pdf]
