# Supplementary material for: Genotype-Specific Activation of Autophagy during Heat Wave in Wheat
Source: Cells. 2024 Jul 20;13(14):1226. doi: 10.3390/cells13141226 (PMC11274669; doi:10.3390/cells13141226)
Supplement: Supplementary file 1 [file cells-13-01226-s001.zip › Supplemental Figures.pdf]

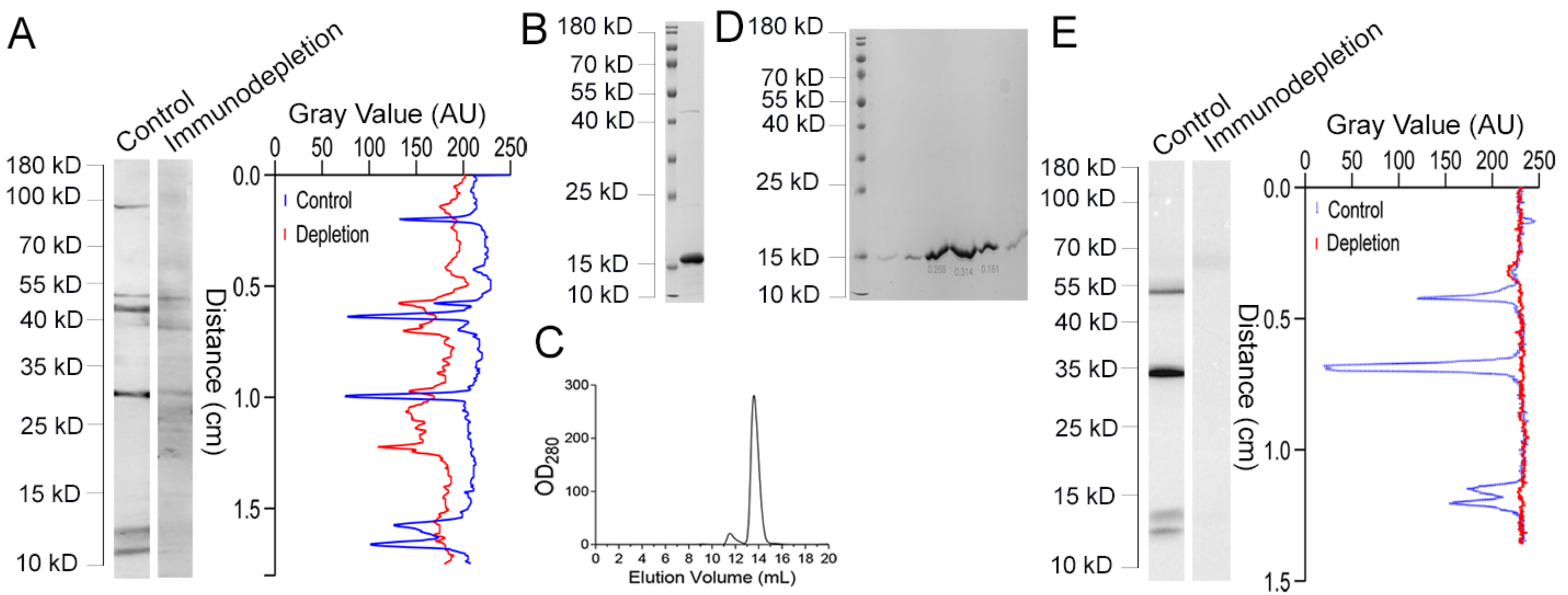

Supplemental Figure S1. Characterization of anti-ATG8.

A, Western blotting images and corresponding densitometric scans of the total leaf extract from Berkut with anti-ATG8 or immunodepleted anti-ATG8. Pre-incubation of the serum with ATG8 protein depletes only some bands. In this experiment bleed 5 was used.

B, SDS-PAGE gel of recombinant ATG8 protein expressed in *E. coli* after purification by Ni-affinity chromatography.

C, Elution profile of ATG8 from the size-exclusion column in 4M Guanidine-HCl buffer.

D, SDS-PAGE gel of fractions 11-16 corresponding to the peaks on the elution profile in panel **C**.

E, Western blotting images and corresponding densitometric scans of total leaf extract from Berkut probed with purified ATG8 antibody or with the immunodepletd an tibody. Immunodepleted with ATG8 abrogates the signal.

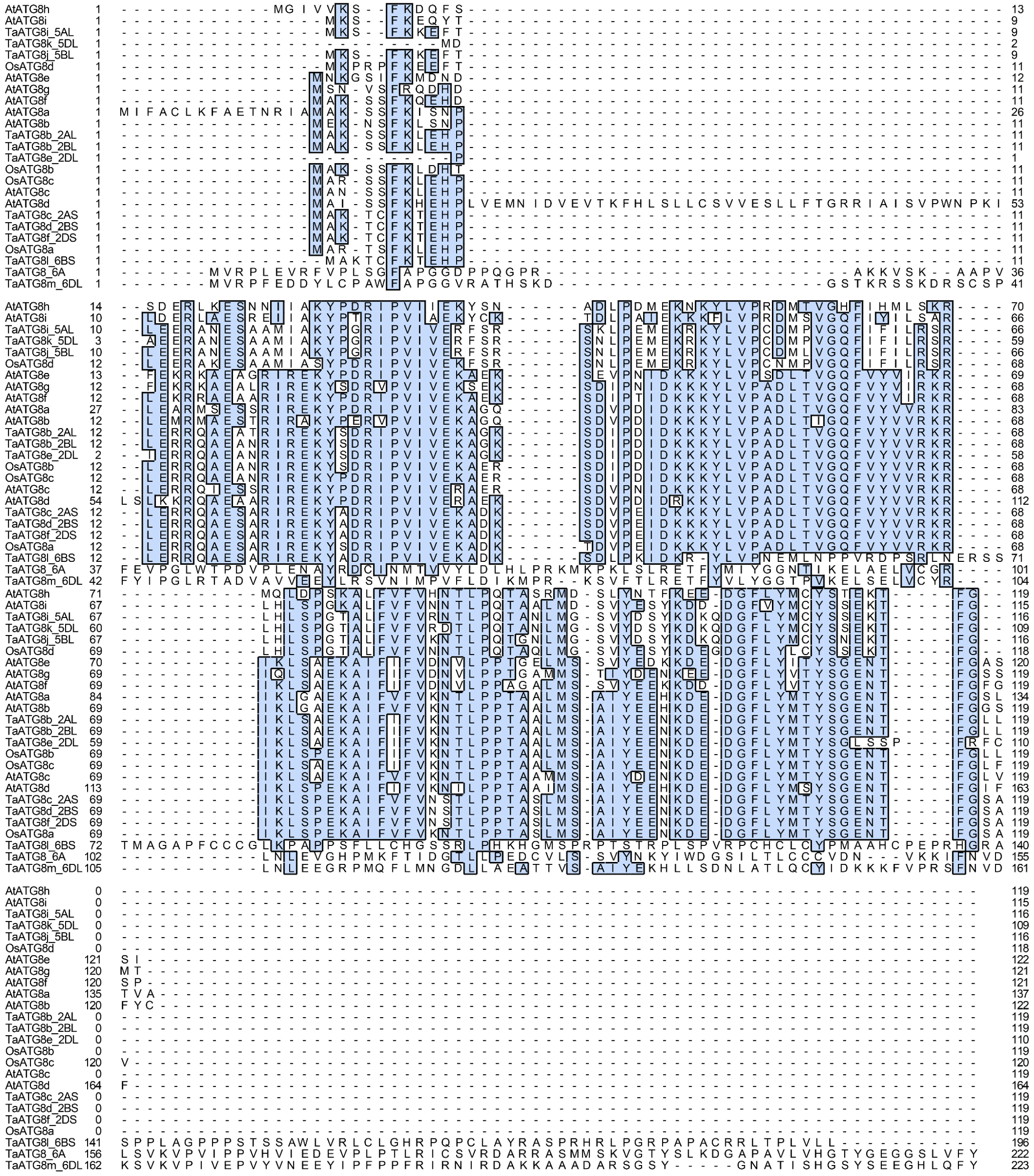

Supplemental Figure S2. Alignment of ATG8 sequences. Conserved regions are highlighted in blue.

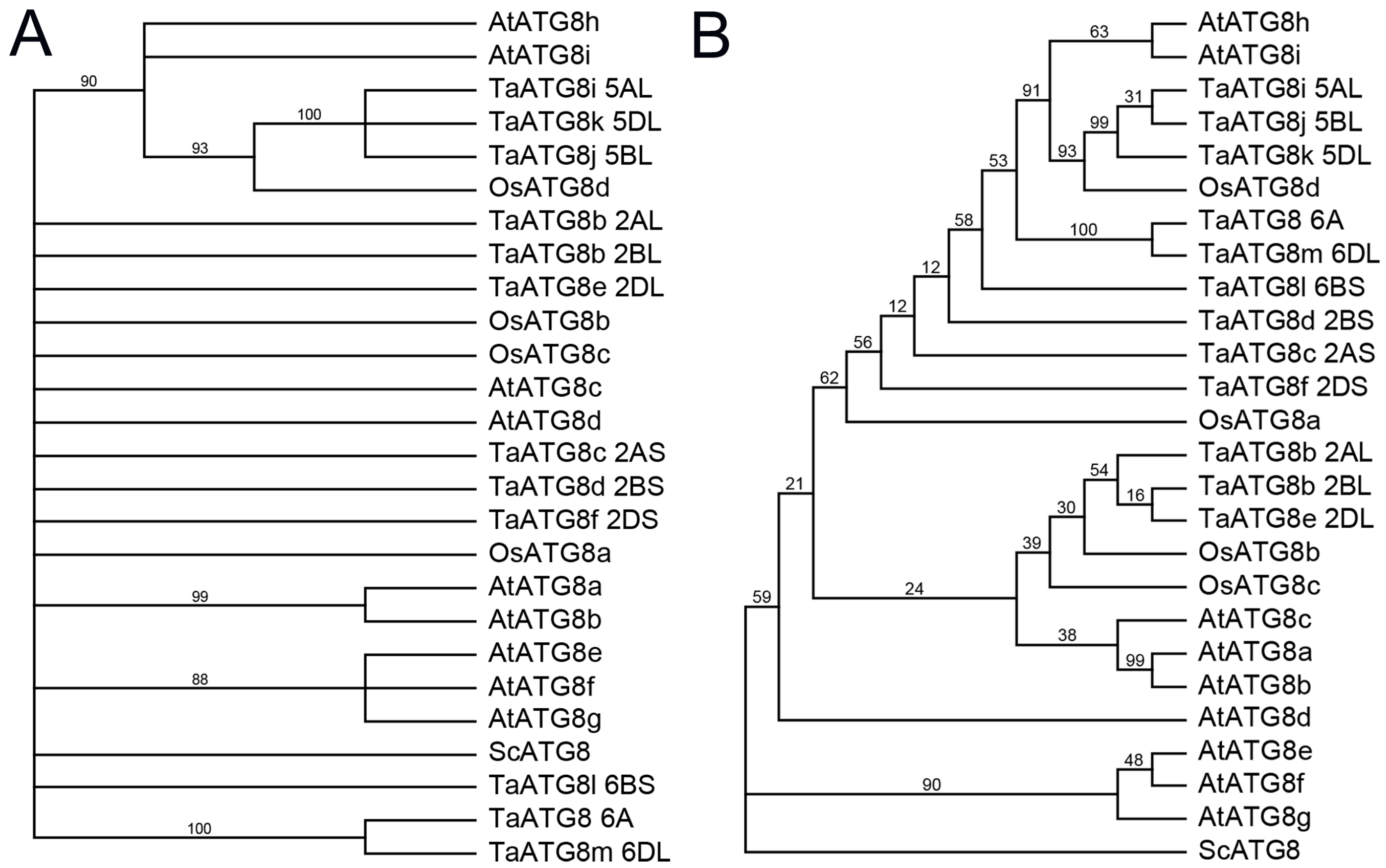

Supplemental Figure S3. Phylogenetic analysis of ATG8.

A,**B**, The phylodendrograms of ATG8 protein sequences showing clades with bootstrap value above 70% (**A**) or all significant clades (**B**). Ta, *Triticum aestivum*; At, *Arabidopsis thaliana*; Os, *Oryza sativa*. Sc, *Saccharomyces cerevisiae* ATG8 was used as the outgroup.

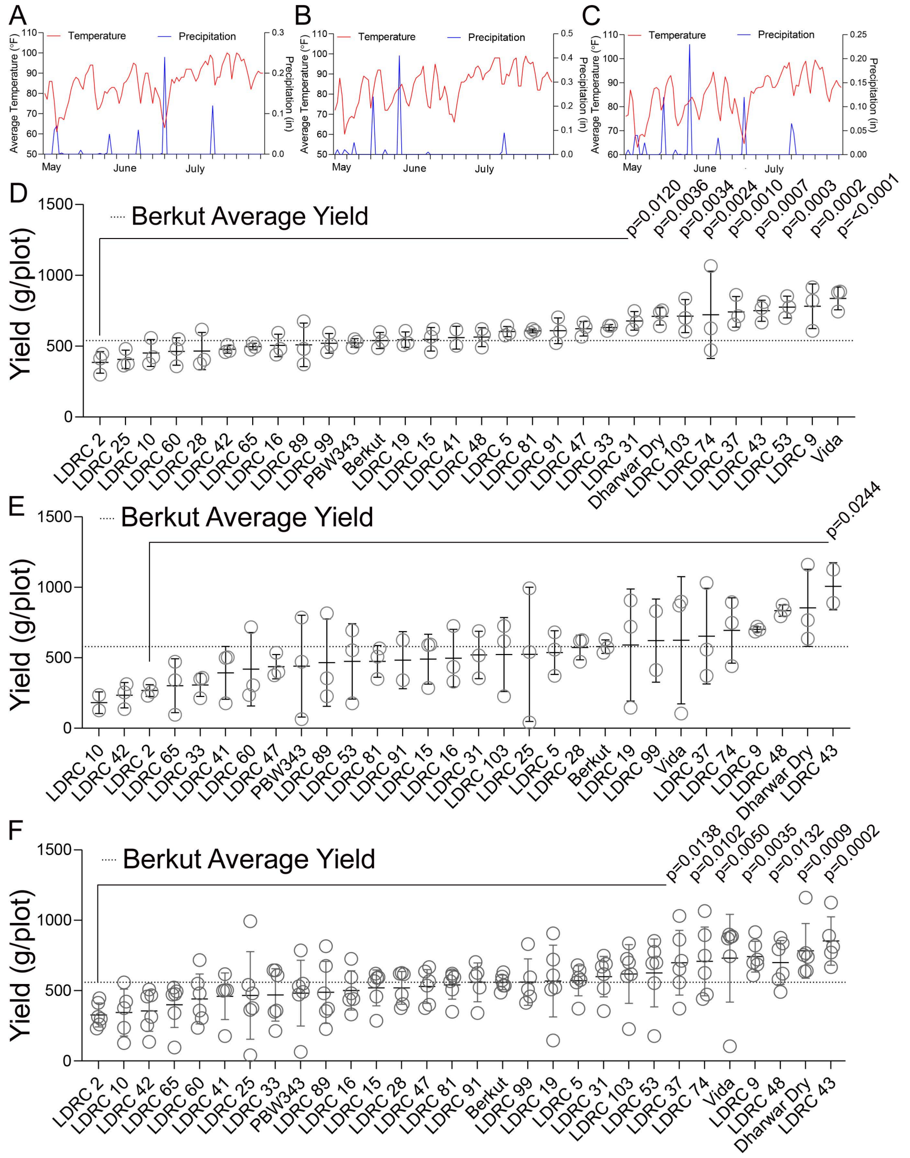

Supplemental Figure S4. Analysis of yield in the field trials. Temperature and precipitation accumulation in Lind, WA (**A**), Othello, WA (**B**) and the average of both sites (**C**) during May-July, 2023. Yield of lines from the spring wheat genetic diversity panel, grown in Lind, WA (**D**)and Othello, WA (**E**), and the average from both sites (**F**). Statistical significance of yield differences was calculated using one-way Anova. Each genotype was compared to LDRC2.

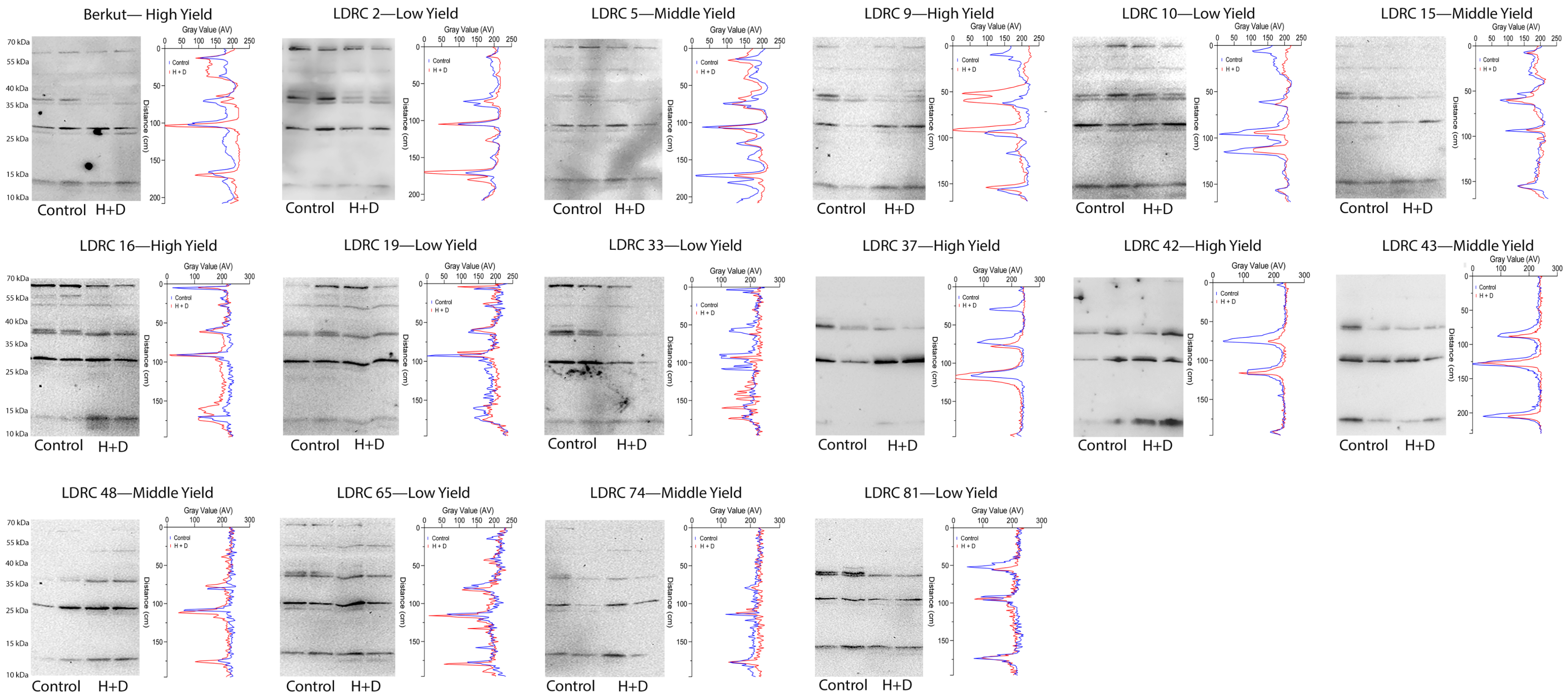

Supplemental Figure S5. Impact of heat and drought stress on ATG8 isoforms. Western blots of total protein extracts from control and heat+drought stressed plants with anti-ATG8. Each membrane contains two biological replicates (independent plants) for each control and heat + drought treatments. The extracts were collected from the first experiment.

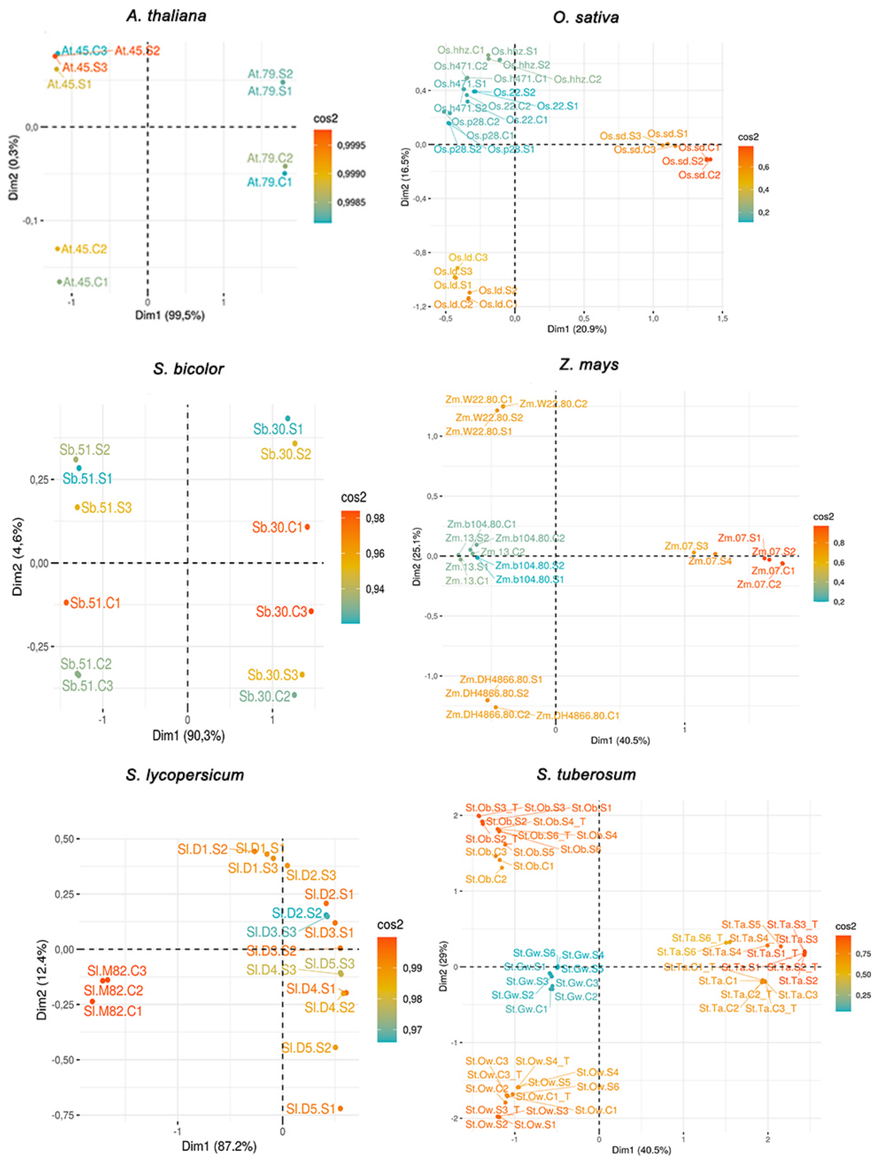

Supplemental Figure S6. Principal component analysis biplots of the RNA-Seq datasets. The analysis was performed using the variance stabilized transformed expression values of the first 2000 genes which show high variance in *A. thaliana, O. sativa, Z. mays, S. bicolor, S. tuberosum, S. lycopersicum* datasets. Squared cosine (cos2) indicates the contribution of each sample to variation.

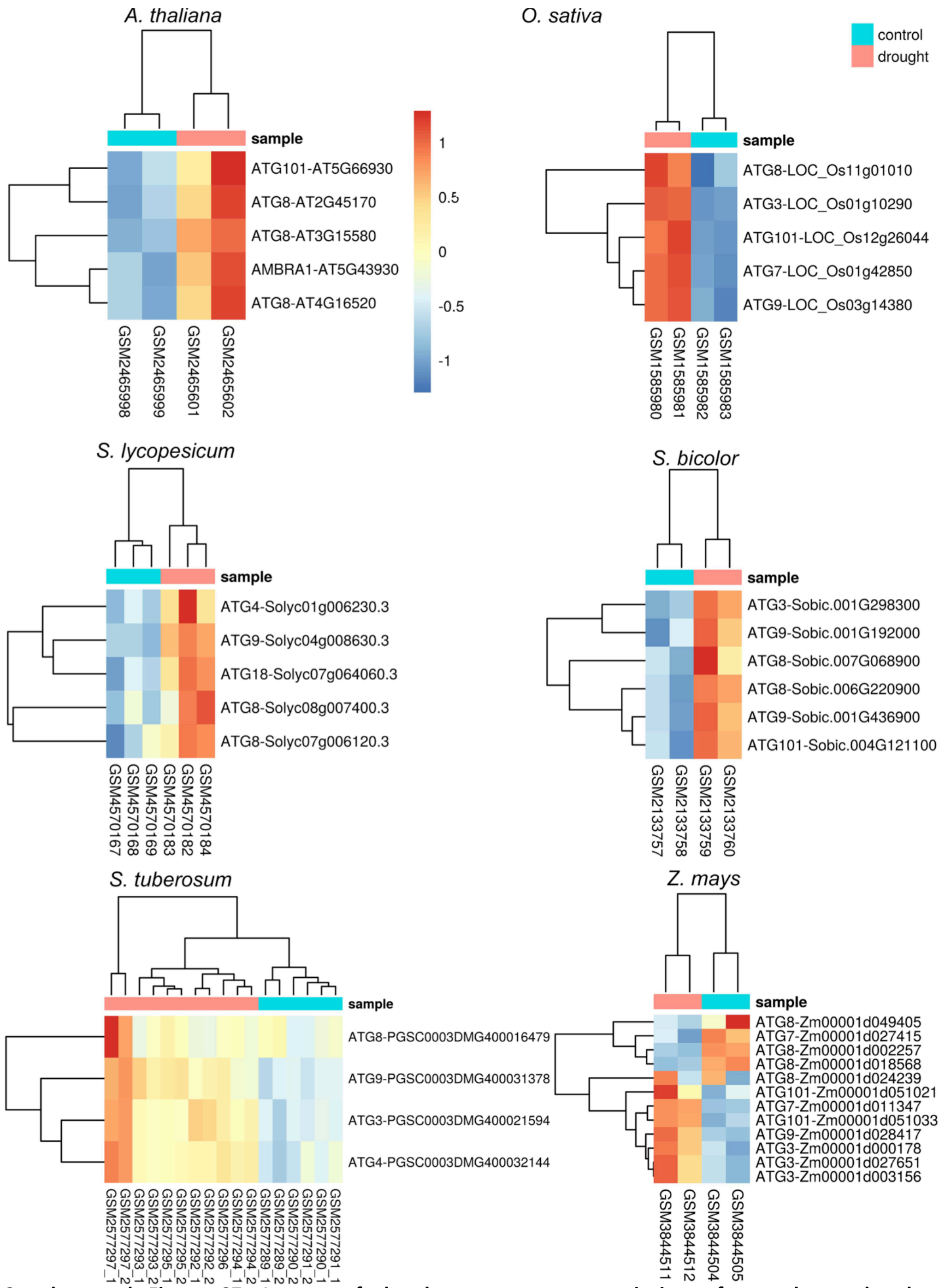

Supplemental Figure S7. Impact of dought stress on transcription of autophagy-related genes in different plant species. Heatmaps of autophagy-related genes that are differentially expressed in response to drought stress. The figures were generated with the pheatmap package using VarianceStabilizedTransformation(VST) -vst() function- built with the DESeq2 package. VST values were represented based on the z-score transformation. Loci name of orthologs that were mapped to autophagy-related genes are indicated beside gene names.

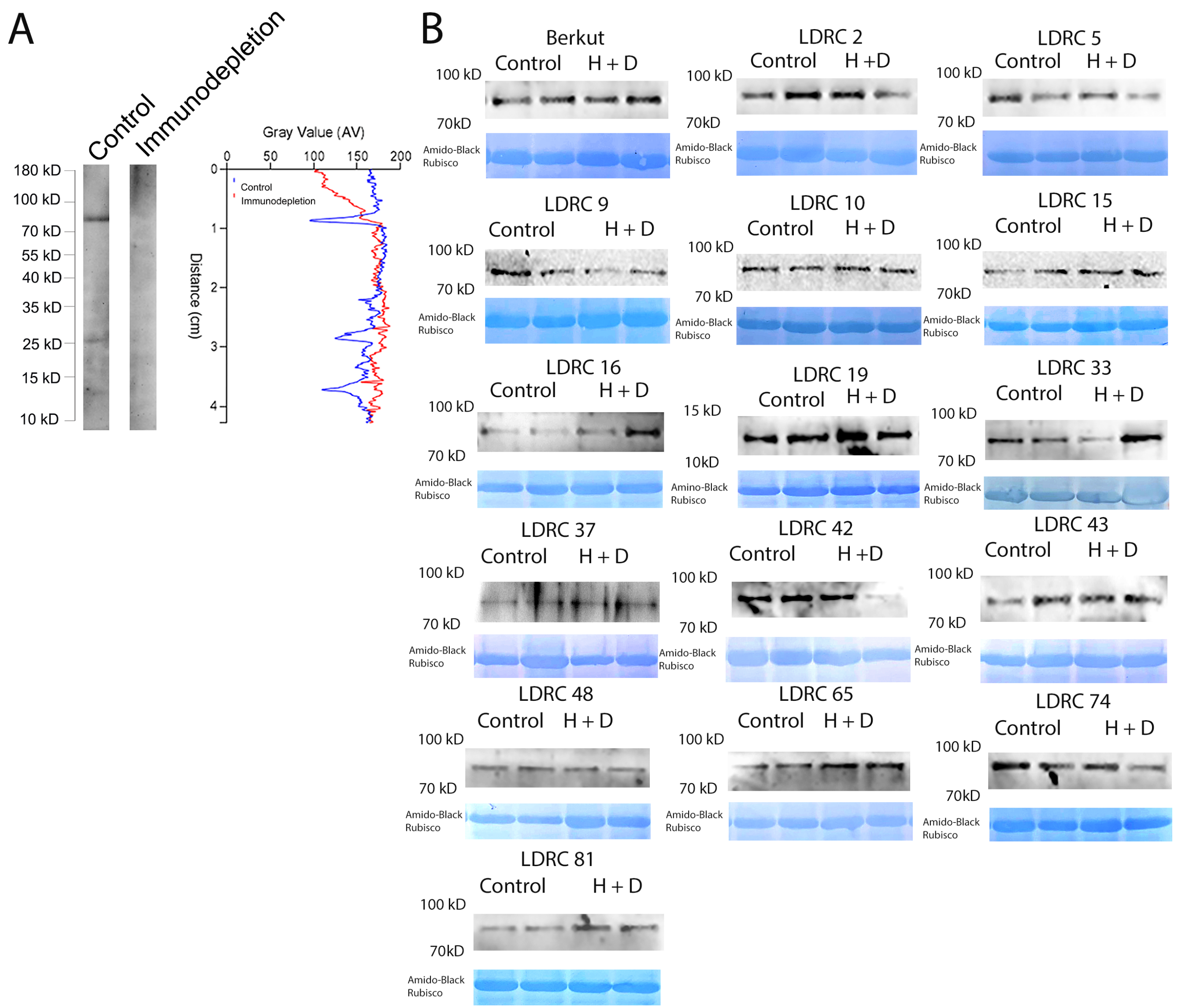

Supplemental Figure S8. The impact of heat and drought stress on ATG7.

A, Western blotting images and corresponding densitometric scans of the total leaf extract from Berkut probed with anti-ATG7 or immunodepleted anti-ATG7. Pre-incubation of the serum with ATG7 protein abrogates the signal.

B, Western blots of total protein extract from control and heat and drought stressed plants with anti-ATG7. Each membrane contains two biological replicates for well water control samples and two heat + drought samples. This material was collected during the first heat + drought treatment (set 2).

A

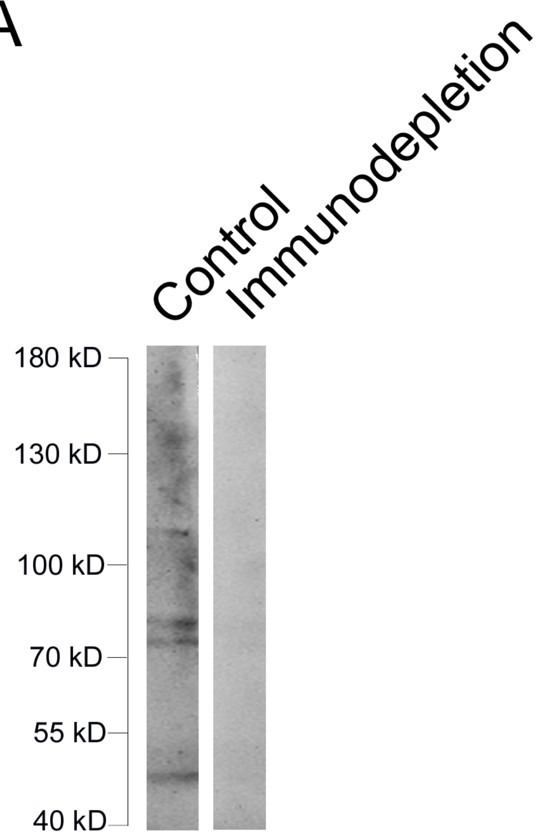

Supplemental Figure S9. Characterization of anti-NBR1.

A, Western blotting images and corresponding densitometric scans of the total leaf extract from Berkut probed with anti-NBR1 or immunodepleted anti-NBR1. Pre-incubation of the serum with NBR1 protein abrogates the signal.
